# Exact learning dynamics of deep linear networks with prior knowledge^*^

**DOI:** 10.1088/1742-5468/ad01b8

**Published:** 2023-11-15

**Authors:** Clémentine C J Dominé, Lukas Braun, James E Fitzgerald, Andrew M Saxe

**Affiliations:** 1 Gatsby Computational Neuroscience Unit, University College London, London, United Kingdom; 2 Department of Experimental Psychology, University of Oxford, Oxford, United Kingdom; 3 Howard Hughes Medical Institute, Janelia Research Campus, Ashburn, Virginia, VA, United States of America; 4 Sainsbury Wellcome Centre, University College London, London, United Kingdom; 5 CIFAR Azrieli Global Scholar, CIFAR, Toronto, Canada

**Keywords:** deep learning, learning theory, machine learning

## Abstract

Learning in deep neural networks is known to depend critically on the knowledge embedded in the initial network weights. However, few theoretical results have precisely linked prior knowledge to learning dynamics. Here we derive exact solutions to the dynamics of learning with rich prior knowledge in deep linear networks by generalising Fukumizu’s matrix Riccati solution (Fukumizu 1998 *Gen* **1** 1E–03). We obtain explicit expressions for the evolving network function, hidden representational similarity, and neural tangent kernel over training for a broad class of initialisations and tasks. The expressions reveal a class of task-independent initialisations that radically alter learning dynamics from slow non-linear dynamics to fast exponential trajectories while converging to a global optimum with identical representational similarity, dissociating learning trajectories from the structure of initial internal representations. We characterise how network weights dynamically align with task structure, rigorously justifying why previous solutions successfully described learning from small initial weights without incorporating their fine-scale structure. Finally, we discuss the implications of these findings for continual learning, reversal learning and learning of structured knowledge. Taken together, our results provide a mathematical toolkit for understanding the impact of prior knowledge on deep learning.

## 1. Introduction

A hallmark of human learning is our exquisite sensitivity to prior knowledge: what we already know affects how we subsequently learn (Carey 1985). For instance, having learned about the attributes of nine animals, we may learn about the tenth more quickly (McClelland *et al* 1995, Murphy 2004, McClelland 2013, Flesch *et al* 2018). In machine learning, the impact of prior knowledge on learning is evident in a range of paradigms including reversal learning (Erdeniz and Atalay 2010), transfer learning (Taylor and Stone 2009, Thrun and Pratt 2012, Lampinen and Ganguli 2018, Gerace *et al* 2022), continual learning (Kirkpatrick *et al* 2017, Zenke *et al* 2017, Parisi *et al* 2019), curriculum learning (Bengio *et al* 2009), and meta learning (Javed and White 2019). One form of prior knowledge in deep networks is the initial network state, which is known to strongly impact learning dynamics (Saxe *et al* 2014, Pennington *et al* 2017, Bahri *et al* 2020). Even random initial weights of different variance can yield qualitative shifts in network behaviour between the *lazy* and *rich* regimes (Chizat *et al* 2019), imparting distinct inductive biases on the learning process. More broadly, rich representations such as those obtained through pretraining provide empirically fertile inductive biases for subsequent fine-tuning (Raghu *et al* 2019). Yet while the importance of prior knowledge to learning is clear, our theoretical understanding remains limited, and fundamental questions remain about the implicit inductive biases of neural networks trained from structured initial weights. A better understanding of the impact of initialisation on gradient-based learning may lead to improved pretraining schemes and illuminate pathologies like catastrophic forgetting in continual learning (McCloskey and Cohen 1989).

**Figure 1.**
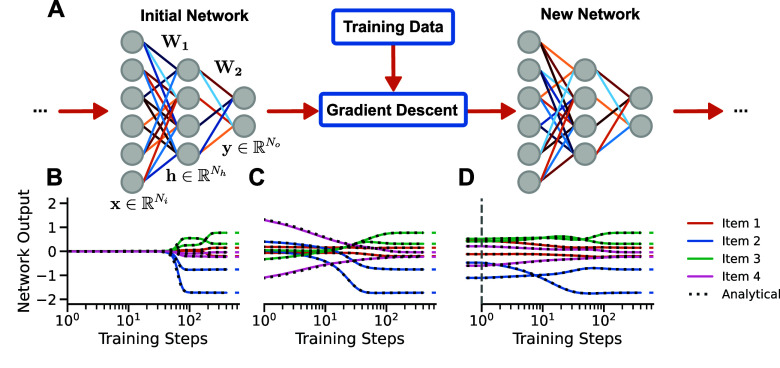
Learning with prior knowledge. (A) In our setting, a deep linear network with $N_\textrm i$ input, $N_\textrm h$ hidden and $N_\textrm o$ output neurons is trained from a particular initialisation using gradient descent. (B)–(D) Network output for an example task over training time when starting from (B) small random weights, (C) large random weights, and (D) the weights of a previously learned task. The dynamics depend in detail on the initialisation. Solid lines indicate simulations, dotted lines indicate the analytical solutions we derive in this work.

Here, we address this gap by deriving exact solutions to the dynamics of learning in deep linear networks as a function of network initialisation, revealing an intricate and systematic dependence. We consider the setting depicted in figure 1(A), where a network is trained with standard gradient descent from a potentially complex initialisation. When trained on the same task, different initialisations can radically change the network’s learning trajectory (figures 1(B)–(D)). Our approach, based on a matrix Riccati formalism (Fukumizu 1998), provides explicit analytical expressions for the network output over time (figures 1(B)–(D) dotted). While simple, deep linear networks have a non-convex loss landscape and have been shown to recapitulate several features of nonlinear deep networks while retaining mathematical tractability.

### 1.1. Contributions

• We derive an explicit solution for the gradient flow of the network function, internal representational similarity, and finite-width neural tangent kernel (NTK) of over- and under-complete two-layer deep linear networks for a rich class of initial conditions (section 3).
• We characterise a set of random initial network states that exhibit fast, exponential learning dynamics and yet converge to *rich* neural representations. Dissociating fast and slow learning dynamics from the *rich* and *lazy* learning regimes (section 4).
• We analyse how weights dynamically align to task-relevant structure over the course of learning, going beyond prior work that has assumed initial alignment (section 5).
• We provide exact solutions to continual learning dynamics, reversal learning dynamics and to the dynamics of learning and revising structured representations (section 6).

### 1.2. Related work

Our work builds on analyses of deep linear networks (Baldi and Hornik 1989, Fukumizu 1998, Saxe *et al* 2014, 2019, Lampinen and Ganguli 2018, Arora *et al* 2018a, Tarmoun *et al* 2021, Atanasov *et al* 2022), which have shown that this simple model nevertheless has intricate fixed point structure and nonlinear learning dynamics reminiscent of phenomena seen in nonlinear networks. A variety of works has analysed convergence (Arora *et al* 2018b, Du and Hu 2019), generalisation (Lampinen and Ganguli 2018, Poggio *et al* 2018, Huh 2020), and the implicit bias of gradient descent (Gunasekar *et al* 2018, Ji and Telgarsky 2018, Laurent and Brecht 2018, Arora *et al* 2019a). These works mostly considers the *tabula rasa* case of small initial random weights, for which exact solutions are known (Saxe *et al* 2014). By contrast our formalism describes dynamics from a much larger class of initial conditions and can describe alignment dynamics that do not occur in the *tabula rasa* setting. Most directly, our results build from the matrix Riccati formulation proposed by Fukumizu (1998). Connecting this formulation and matrix factorisation problems yields a better characterisation of the convergence rate (Tarmoun *et al* 2021). We extend and refine the matrix Riccati result to obtain the dynamics of over- and under-complete networks; to obtain numerically stable forms of the matrix equations; and to more explicitly reveal the impact of initialisation.

A line of theoretical research has considered online learning dynamics in teacher-student settings (Biehl and Schwarze 1995, Saad and Solla 1995, Goldt *et al* 2019), deriving ordinary differential equations for the average learning dynamics even in nonlinear networks. However, solving these equations requires numerical integration. By contrast, our approach provides explicit analytical solutions for the more restricted case of deep linear networks.

Other approaches for analysing deep network dynamics include the NTK (Jacot *et al* 2018, Lee *et al* 2019, Arora *et al* 2019b) and the mean field approach (Mei *et al* 2018, Rotskoff and Vanden-Eijnden 2018, Sirignano and Spiliopoulos 2020). While the former can describe nonlinear networks but not the learning dynamics of hidden representations, the later yields a description of representation learning dynamics in wide networks in terms of a partial differential equation. Our work is similar in seeking a subset of more tractable models that are amenable to analysis, but we focus on the impact of initialisation on representation learning dynamics and explicit solutions.

A large body of work has investigated the effect of different random initialisations on learning in deep networks. The role of initialisation in the vanishing gradient problem and proposals for better initialisation schemes have been illuminated by several works drawing on the central limit theorem (Glorot and Bengio 2010, Saxe *et al* 2014, He *et al* 2015, Pennington *et al* 2017, Xiao *et al* 2018), reviewed in Carleo *et al* (2019), Arora *et al* (2020), Bahri *et al* (2020). These approaches typically guarantee that gradients do not vanish at the start of learning, but do not analytically describe the resulting learning trajectories. Influential work has shown that network initialisation variance mediates a transition from *rich* representation learning to *lazy* NTK dynamics (Chizat *et al* 2019), which we analyse in our framework.

## 2. Preliminaries and setting

Consider a supervised learning task in which input vectors $\mathbf{x}_n \in \mathbb{R}^{N_\textrm i}$ from a set of *P* training pairs $\{(\mathbf{x}_n, \mathbf{y}_n)\}_{n = 1{\ldots}P}$ have to be associated with their target output vectors $\mathbf{y}_n \in \mathbb{R}^{N_\textrm o}$. We learn this task with a two-layer linear network model (figure 1(A)) that produces the output prediction

\begin{equation*} {\mathbf{\hat{y}}}_n = \mathbf{W}_2\mathbf{W}_1 \mathbf{x}_n\text{,} \end{equation*}

with weight matrices $\mathbf{W}_1 \in \mathbb{R}^{N_\textrm h\times N_\textrm i}$ and $\mathbf{W}_2 \in \mathbb{R}^{N_\textrm o\times N_\textrm h}$, where $N_\textrm h$ is the number of hidden units. The network’s weights are optimised using full batch gradient descent with learning rate *η* (or respectively time constant $\tau = \frac{1}{\eta}$) on the mean squared error loss

\begin{equation*} \mathcal{L}\left({\mathbf{\hat{y}}}, \mathbf{y}\right) = \frac{1}{2}\left\langle ||{\mathbf{\hat{y}}} - \mathbf{y}||^2\right\rangle, \end{equation*}

where $\langle\cdot \rangle$ denotes the average over the dataset. The input and input-output correlation matrices of the dataset are

\begin{equation*} \boldsymbol{\tilde{\Sigma}}^{xx} = \frac{1}{P}\sum_{n = 1}^P \mathbf{x}_n\mathbf{x}_n^{\textrm T}\ \in \mathbb{R}^{N_\textrm i\times N_\textrm i} \quad\text {and}\quad\, \boldsymbol{\tilde{\Sigma}}^{yx} = \frac{1}{P}\sum_{n = 1}^P\mathbf{y}_n\mathbf{x}_n^{\textrm T}\ \in \mathbb{R}^{N_\textrm o\times N_\textrm i}\text{.} \end{equation*}

Finally, the gradient optimisation starts from an initialisation $\mathbf{W}_2(0),\mathbf{W}_1(0)$. Our goal is to understand the full time trajectory of the network’s output and internal representations as a function of this initialisation and the task statistics.

Our starting point is the seminal work of Fukumizu (Fukumizu 1998), which showed that the gradient flow dynamics could be written as a matrix Riccati equation with known solution. In particular, defining

\begin{equation*} \mathbf{Q} = \begin{bmatrix} \mathbf{W}_1^{\textrm T} \\ \mathbf{W}_2 \end{bmatrix} \text{ and }\, \mathbf{F} = \begin{bmatrix} 0 &amp; \left(\boldsymbol{\tilde{\Sigma}}^{yx}\right)^{\textrm T}\\ \boldsymbol{\tilde{\Sigma}}^{yx} &amp; 0 \end{bmatrix} \text{,} \end{equation*}

the continuous time dynamics of the matrix $\mathbf{Q}\mathbf{Q}^{\textrm T}$ from initial state $\mathbf{Q}(0)$ is

\begin{equation*} \mathbf{Q}\mathbf{Q}^{\textrm T}\left(t\right) = e^{\mathbf{F}\frac{t}{\tau}}\mathbf{Q}\left(0\right)\left[\mathbf{I} + \frac{1}{2}\mathbf{Q}\left(0\right)^{\textrm T}\left(e^{\mathbf{F}\frac{t}{\tau}}\mathbf{F}^{-1}e^{\mathbf{F}\frac{t}{\tau}} - \mathbf{F}^{-1} \right) \mathbf{Q}\left(0\right)\right]^{-1} \mathbf{Q}\left(0\right)^{\textrm T}e^{\mathbf{F}\frac{t}{\tau}} \text{,} \end{equation*}

if the following four assumptions hold:

Assumption 2.1. The dimensions of the input and target vectors are identical, that is $N_\textrm i = N_\textrm o$.

Assumption 2.2. The input data is whitened, that is $\mathbf{\tilde{\Sigma}}^{xx} = \mathbf{I}$.

Assumption 2.3. The network’s weight matrices are zero-balanced at the beginning of training, that is $\mathbf{W}_1(0)\mathbf{W}_1(0)^{\textrm T} = \mathbf{W}_2(0)^{\textrm T}\mathbf{W}_2(0)$. If this condition holds at initialisation, it will persist throughout training (Saxe *et al* 2014, Arora *et al* 2018a).

Assumption 2.4. The input-output correlation of the task and the initial state of the network function have full rank, that is $\mathrm{rank}(\mathbf{\tilde{\Sigma}}^{xy}) = \,\mathrm{rank}(\mathbf{W}_2(0)\mathbf{W}_1(0)) = N_\textrm i = N_\textrm o$. This implies that the network is not bottlenecked, i.e. $N_\textrm h \unicode{x2A7E} \min(N_\textrm i, N_\textrm o)$.

For completeness, we include a derivation of this solution in appendix A.

Rather than tracking the weights’ dynamics directly, this approach tracks several key statistics collected in the matrix

**Figure:**
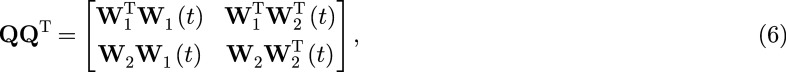


 which can be separated into four quadrants with intuitive meaning: the off-diagonal blocks contain the network function

**Figure:**
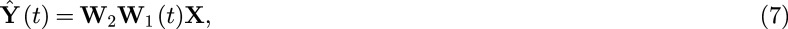


 while the on-diagonal blocks contain the correlation structure of the weight matrices. These permit calculation of the temporal evolution of the network’s internal representations including the task-relevant representational similarity matrices (RSMs) (Kriegeskorte *et al* 2008), i.e. the kernel matrix $\phi(x)^{\textrm T}\phi(x^{^{\prime}})$, of the neural representations in the hidden layer

**Figure:**
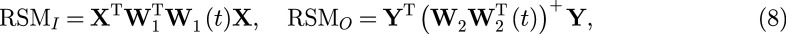


 where + denotes the pseudoinverse; and the network’s finite-width NTK (Jacot *et al* 2018, Lee *et al* 2019, Arora *et al* 2019b)

**Figure:**
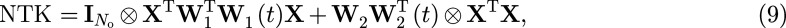


where **I** is the identity matrix and ⊗ is the Kronecker product. For a derivation of these quantities see appendix B. Hence, the solution in equation (5) describes important aspects of network behaviour.

However, in this form, the solution has several limitations. First, it relies on general matrix exponentials and inverses, which are a barrier to explicit understanding. Second, when evaluated numerically, it is often unstable. And third, the equation is only valid for equal input and output dimensions. In the following section we address these limitations.

Implementation and simulation. Simulation details are in appendix H. Code to replicate all simulations and plots are available online^6^ 6
https://github.com/saxelab/deep-linear-networks-with-prior-knowledge. under a *GPLv3* license and requires $\lt$6 h to execute on a single AMD Ryzen 5950x.

## 3. Exact learning dynamics with prior knowledge

In this section we derive an exact and numerically stable solution for $\mathbf{Q}\mathbf{Q}^{\textrm T}$ that better reveals the learning dynamics, convergence behaviour and generalisation properties of two-layer linear networks with prior knowledge. Further, we alter the equations to be applicable to equal and unequal input and output dimensions, overcoming assumption 2.1.

To place the solution in a more explicit form, we make use of the compact singular value decomposition. Let the compact singular value decomposition of the initial network function and the input-output correlation of the task be

\begin{equation*} \mathrm{SVD}\left(\mathbf{W}_2\left(0\right)\mathbf{W}_1\left(0\right)\right) = \mathbf{U}\mathbf{S}\mathbf{V}^{\textrm T} \quad\text{and} \quad \mathrm{SVD}\left(\boldsymbol{\tilde{\Sigma}}^{yx}\right) = {\tilde{\mathbf{U}}}{\tilde{\mathbf{S}}}{\tilde{\mathbf{V}}}^{\textrm T} \text{.} \end{equation*}

Here, **U** and ${\tilde{\mathbf{U}}} \in \mathbb{R}^{N_\mathrm o \times N_m}$ denote the left singular vectors, **S** and ${\tilde{\mathbf{S}}} \in \mathbb{R}^{N_m \times N_m}$ the square matrix with ordered, non-zero eigenvalues on its diagonal and **V** and $\mathbf{\tilde{V}} \in \mathbb{R}^{N_\textrm i \times N_m}$ the corresponding right singular vectors. For unequal input-output dimensions ($N_\textrm i \neq N_\textrm o$) the right and left singular vectors are therefore not generally square and orthonormal. Accordingly, for the case $N_\textrm i > N_\textrm o$, we define ${\tilde{\mathbf{U}}_\perp} \in \mathbb{R}^{N_\mathrm o \times (N_\mathrm o - N_\textrm i)}$ as a matrix containing orthogonal column vectors that complete the basis, i.e. make $\big[{\tilde{\mathbf{U}}} ~ {\tilde{\mathbf{U}}_\perp}\big]$ orthonormal. Conversely, we define ${\tilde{\mathbf{V}}_\perp} \in \mathbb{R}^{N_\textrm i \times (N_\textrm i - N_\mathrm o)}$ for the case of $N_\textrm i > N_\textrm o$.

Assumption 3.1. Define $\mathbf{B} = \mathbf{U}^\mathrm {T}{\tilde{\mathbf{U}}} +\mathbf{V}^\mathrm {T}\mathbf{\tilde{V}}$ and $\mathbf{C} = \mathbf{U}^\mathrm {T}{\tilde{\mathbf{U}}} -\mathbf{V}^\mathrm {T}\mathbf{\tilde{V}}$. **B** is non-singular.

Theorem 3.1. Under the assumptions of whitened inputs, 2.2, zero-balanced weights 2.3, full rank 2.4, and **B** non-singular 3.1, the temporal dynamics of $\mathbf{Q}\mathbf{Q}^{\textrm T}$ are

\begin{align*} \mathbf{Q}\mathbf{Q}^{\textrm T}\left(t\right) &amp;= \mathbf{Z} \left[ 4e^{-{\tilde{\mathbf{S}}} \frac{t}{\tau}}\mathbf{B}^{-1}{\mathbf{S}}^{-1}\left(\mathbf{B}^\mathrm {T}\right)^{-1}e^{-{\tilde{\mathbf{S}}} \frac{t}{\tau}} + \left( \mathbf{I} - e^{-2{\tilde{\mathbf{S}}} \frac{t}{\tau}} \right){\tilde{\mathbf{S}}}^{-1} \right. \nonumber\\ &amp;\quad-e^{-{\tilde{\mathbf{S}}} \frac{t}{\tau}}\mathbf{B}^{-1}\mathbf{C}\left(\ e^{-2{\tilde{\mathbf{S}}} \frac{t}{\tau}} - \mathbf{I} \right){\tilde{\mathbf{S}}}^{-1}\mathbf{C}^{\textrm T}\left(\mathbf{B}^{T}\right)^{-1}e^{-{\tilde{\mathbf{S}}} \frac{t}{\tau}} \nonumber\\ &amp;\quad \left. +4 \frac{t}{\tau} e^{-{\tilde{\mathbf{S}}} \frac{t}{\tau}}\mathbf{B}^{-1} \left(\mathbf{V}^{\textrm T}{\tilde{\mathbf{V}}_\perp}^{} {\tilde{\mathbf{V}}_\perp}^{\textrm T}\mathbf{V}+\mathbf{U}^{\textrm T}{\tilde{\mathbf{U}}_\perp}^{}{\tilde{\mathbf{U}}_\perp}^{\textrm T}\mathbf{U}\right)\left(\mathbf{B}^\mathrm {T}\right)^{-1} e^{-{\tilde{\mathbf{S}}} \frac{t}{\tau}} \right]^{-1}\mathbf{Z}^\mathrm {T} \end{align*}

with

\begin{align*} \mathbf{Z} = \begin{bmatrix} {\tilde{\mathbf{V}}} \left(\mathbf{I} - e^{-{\tilde{\mathbf{S}}} \frac{t}{\tau}}\mathbf{C}^{\textrm T}\left(\mathbf{B}^\mathrm {T}\right)^{-1}e^{-{\tilde{\mathbf{S}}} \frac{t}{\tau}}\right)+2 {\tilde{\mathbf{V}}_\perp}^{}{\tilde{\mathbf{V}}_\perp}^\mathrm {T}\mathbf{V} \left(\mathbf{B}^\mathrm {T}\right)^{-1} e^{-{\tilde{\mathbf{S}}}\frac{t}{\tau}}\\[4pt] {\tilde{\mathbf{U}}} \left(\mathbf{I} + e^{-{\tilde{\mathbf{S}}}\frac{t}{\tau}} \mathbf{C}^{\textrm T}\left(\mathbf{B}^\mathrm {T}\right)^{-1}e^{-{\tilde{\mathbf{S}}} \frac{t}{\tau}} \right) + 2{\tilde{\mathbf{U}}_\perp}^{}{\tilde{\mathbf{U}}_\perp}^\mathrm {T}\mathbf{U}\left(\mathbf{B}^\mathrm {T}\right)^{-1} e^{-{\tilde{\mathbf{S}}}\frac{t}{\tau}} \end{bmatrix} .\end{align*}

For a proof of theorem 3.1 please refer to appendix C.

**Figure 2.**
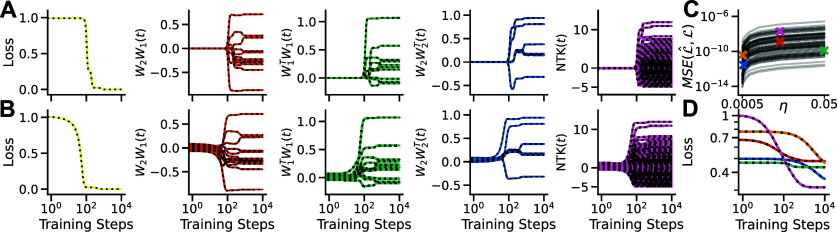
Exact learning dynamics (A) the temporal dynamics of the numerical simulation (coloured lines) of the loss, network function, correlation of input and output weights and the NTK (columns 1–5 respectively) are exactly matched by the analytical solution (black dotted lines) for small initial weight values and (B) large initial weight values. (C) Each line shows the deviation of the analytical loss $\hat{\mathcal{L}}$ from the numerical loss $\mathcal{L}$ for one of *n* = 50 networks with random architecture and training data (details in appendix H) across a range of learning rates $\eta \in [0.05, 0.0005]$. The deviation mutually decreases with the learning rate. (D) Numerical and analytical learning curves for five randomly sampled example networks (coloured x in (C)).

With this solution we can calculate the exact temporal dynamics of the loss, network function, RSMs and NTK (figures 2(A) and (B)). As the solution contains only negative exponentials, it is numerically stable and provides high precision across a wide range of learning rates and network architectures (figures 2(C) and (D)).

We note that a solution for the weights $\mathbf{W}_1(t)$ and $\mathbf{W}_2(t)$, i.e. $\mathbf{Q} (t)$, can be derived up to a time varying orthogonal transformation as demonstrated in appendix C. Further, as time-dependent variables only occur in matrix exponentials of diagonal matrices of negative sign, the network approaches a steady state solution.

Theorem 3.2. Under the assumptions of theorem 3.1, the network function converges to the global minimum ${\tilde{\mathbf{U}}}{\tilde{\mathbf{S}}}\mathbf{\tilde{V}}^{\textrm T}$ and acquires a rich task-specific internal representation, that is $\mathbf{W}_1^{\textrm T}\mathbf{W}_1 = \mathbf{\tilde{V}}{\tilde{\mathbf{S}}}\mathbf{\tilde{V}}^{\textrm T}$ and $\mathbf{W}_2\mathbf{W}_2^{\textrm T} = {\tilde{\mathbf{U}}}{\tilde{\mathbf{S}}}{\tilde{\mathbf{U}}}^{\textrm T}$.

The proof of theorem 3.2 is in appendix C. We now turn to several implications of these results.

## 4. Rich and lazy learning regimes and generalisation

Recent results have shown that large deep networks can operate in qualitatively distinct regimes that depend on their weight initialisations (Chizat *et al* 2019, Flesch *et al* 2022), the so called *rich* and *lazy* regimes. In the *rich* regime, learning dynamics can be highly nonlinear and lead to task-specific solutions thought to lead to favourable generalisation properties (Chizat *et al* 2019, Saxe *et al* 2019, Flesch *et al* 2022). By contrast, the *lazy* regime exhibits simple exponential learning dynamics and exploits high-dimensional nonlinear projections of the data produced by the initial random weights, leading to task-agnostic representations that attain zero training error but possibly lower generalisation performance (Jacot *et al* 2018, Lee *et al* 2019, Arora *et al* 2019b). Traditionally, the *rich* and *lazy* learning regimes have been respectively linked to low and high variance initial weights (relative to the network layer size).

**Figure 3.**
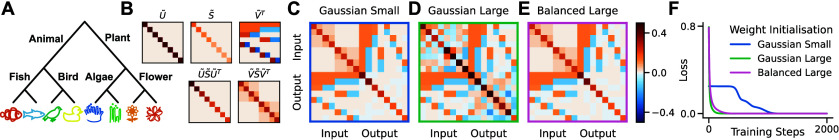
Rich and lazy learning. (A) Semantic learning task, (B) SVD of the input-output correlation of the task (top) and the respective RSMs (bottom). Rows and columns in the SVD and RSMs are identically ordered as the order of items in the hierarchical tree. (C) Final $\mathbf{Q}\mathbf{Q}^{\textrm T}$ matrices after training converged when initialised from random small weights, (D) random large weights (note how the upper left and lower right quadrant differ from the task’s RSMs) and (E) large zero-balanced weights. (F) Learning curves for the three different initialisations as in (C) (green), (D) (pink) and (E) (blue). While both large weight initialisations lead to fast exponential learning curves, the small weight initialisation leads to a slow step-like decay of the loss.

To illustrate these phenomena, we consider a semantic learning task in which a set of living things have to be linked to their position in a hierarchical structure (figure 3(A)) (Saxe *et al* 2014). The representational similarity of the input of the task ($\mathbf{\tilde{V}}{\tilde{\mathbf{S}}}\mathbf{\tilde{V}}^{\textrm T}$) reveals its inherent structure (figure 3(B)). For example, the representations of the two fishes are most similar to each other, less similar to birds and least similar to plants. Likewise, the representational similarity of the task’s target values (${\tilde{\mathbf{U}}}{\tilde{\mathbf{S}}}{\tilde{\mathbf{U}}}^{\textrm T}$) reveals the primary groups among which items are organised. As a consequence, one can for example predict from an object being a fish that it is an animal and from an object being a plant that it is not a bird. Reflecting these structural relationships in internal representations can allow the *rich* regime to generalise in ways the *lazy* regime cannot. Crucially, $\mathbf{Q}\mathbf{Q}^{\textrm T}(t)$ contains the temporal dynamics of the weights’ representational similarity and therefore can be used to study if a network finds a *rich* or *lazy* solution.

When training a two layer network from random small initial weights, the weights’ input and output RSM (figure 3(C), upper left and lower right quadrant) are identical to the task’s structure at convergence. However, when training from large initial weights, the RSM reveals that the network has converged to a *lazy* solution (figure 3(D)). We emphasise that the network function in both cases is identical (figures 3(C) and (D), lower left quadrant). And while their final loss is identical too, their learning dynamics evolve slow and step-wise in the case of small initial weights and fast and exponentially in the case of large initial weights (figure 3(F)), as predicted by previous work (Chizat *et al* 2019).

However, from theorem 3.2 it directly follows that our setup is guaranteed to find a *rich* solution in which the weights’ RSM is identical to the task’s RSM, i.e. $\mathbf{W}_1^{\textrm T}\mathbf{W}_1 = \mathbf{\tilde{V}}{\tilde{\mathbf{S}}}\mathbf{\tilde{V}}^{\textrm T}$ and $\mathbf{W}_2\mathbf{W}_2^{\textrm T} = {\tilde{\mathbf{U}}}{\tilde{\mathbf{S}}}{\tilde{\mathbf{U}}}^{\textrm T}$. Therefore, as zero-balanced weights may be large, there exist initial states that converge to *rich* solutions while evolving as rapid exponential learning curves (figures 3(E) and (F)). Crucially, these initialisations are task-agnostic, in the sense that they are independent of the task structure (see Mishkin and Matas 2015). This finding applies to any learning task with well defined input-output correlation. For additional simulations see appendix D. Hence our equation can describe the change in dynamics from step-like to exponential with increasing weight scale, and separate this dynamical phenomenon from the structure of internal representations.

## 5. Decoupling dynamics

The learning dynamics of deep linear networks depend on the exact initial values of the synaptic weights. Previous solutions studied learning dynamics under the assumption that initial network weights are ‘decoupled’, such that the initial state of the network and the task share the same singular vectors, i.e. that $\mathbf{U} = {\tilde{\mathbf{U}}}$ and $\mathbf{V} = \mathbf{\tilde{V}}$ (Saxe *et al* 2014). Intuitively, this assumption means that there is no cross-coupling between different singular modes, such that each evolves independently. However, this assumption is violated in most real-world scenarios. As a consequence, most prior work has relied on the empirical observation that learning from *tabula rasa* small initial weights occurs in two phases: First, the network’s input-output map rapidly decouples; then subsequently, independent singular modes are learned in this decoupled regime. Because this decoupling process is fast when training begins from small initial weights, the learning dynamics are still approximately described by the temporal learning dynamics of the singular values assuming decoupling from the start. This dynamic has been called a *silent alignment* process (Atanasov *et al* 2022). Here we leverage our matrix Riccati approach to analytically study the dynamics of this decoupling process. We begin by deriving an alternate form of the exact solution that eases the analysis.

Theorem 5.1. Let the weight matrices of a two layer linear network be initialised by $\mathbf{W}_1 = \mathbf{A}(0)\tilde{\mathbf{V}}^{\textrm T}$ and $\mathbf{W}_2 = {\tilde{\mathbf{U}}}\mathbf{A}(0)^{\textrm T}$, where $\mathbf{A}(0) \in \mathbb{R}^{N_\textrm h\times N_\textrm i}$ is an arbitrary, invertible matrix. Then, under the assumptions of equal input-output dimensions 2.1, whitened inputs 2.2, zero-balanced weights 2.3 and full rank 2.4, the temporal dynamics of $\mathbf{Q}\mathbf{Q}^{\textrm T}$ are fully determined by

\begin{equation*} \mathbf{A}^{\textrm T}\mathbf{A}\left(t\right) = \left[e^{-{\tilde{\mathbf{S}}}\frac{t}{\tau}}\left(\mathbf{A}\left(0\right)^{\textrm T}\mathbf{A}\left(0\right)\right)^{-1}e^{-{\tilde{\mathbf{S}}}\frac{t}{\tau}} + \left(\mathbf{I}-e^{-2{\tilde{\mathbf{S}}}\frac{t}{\tau}}\right){\tilde{\mathbf{S}}}^{-1}\right]^{-1}\text{.} \end{equation*}

For a proof of theorem 5.1, please refer to appendix E. We remark that this form is less general than that in theorem 3.1, and in particular implies $\mathbf{U}\mathbf{V} = {\tilde{\mathbf{U}}}\mathbf{\tilde{V}}$. Here the matrix $\mathbf{A}^{\textrm T}\mathbf{A}$ represents the dynamics directly in the SVD basis of the task. Off-diagonal elements represent counterproductive coupling between different singular modes (for instance, $[\mathbf{A}^{\textrm T}\mathbf{A}]_{21}$ is the strength of connection from input singular vector 1 to output singular vector 2, which must approach zero to perform the task perfectly), while on-diagonal elements represent the coupling within the same mode (for instance, $[\mathbf{A}^{\textrm T}\mathbf{A}]_{11}$ is the strength of connection from input singular vector 1 to output singular vector 1, which must approach the associated task singular value to perform the task perfectly). Hence the decoupling process can be studied by examining the dynamics by which $\mathbf{A}^{\textrm T}\mathbf{A}$ becomes approximately diagonal.

The outer inverse in equation (13) renders it difficult to study high dimensional networks analytically. Therefore, we focus on small networks with input and output dimension $N_\textrm i = 2$ and $N_\textrm o = 2$, for which a lengthy but explicit analytical solution is given in appendix E. In this setting, the structure of the weight initialisation and task are encoded in the matrices

\begin{equation*} \mathbf{A}\left(0\right)^{\textrm T}\mathbf{A}\left(0\right) = \begin{bmatrix} a_1\left(0\right) &amp; b\left(0\right) \\ b\left(0\right) &amp; a_2\left(0\right) \end{bmatrix} \quad \text{and}\quad {\tilde{\mathbf{S}}} = \begin{bmatrix} s_1 &amp; 0 \\ 0 &amp; s_2 \end{bmatrix}\text{,} \end{equation*}

where the parameters $a_1(0)$ and $a_2(0)$ represent the component of the initialisation that is aligned with the task, and *b*(0) represents cross-coupling, such that taking $b(0) = 0$ recovers previously known and more restricted solutions for the decoupled case (Saxe *et al* 2014). We use this setting to demonstrate two features of the learning dynamics.

**Figure 4.**
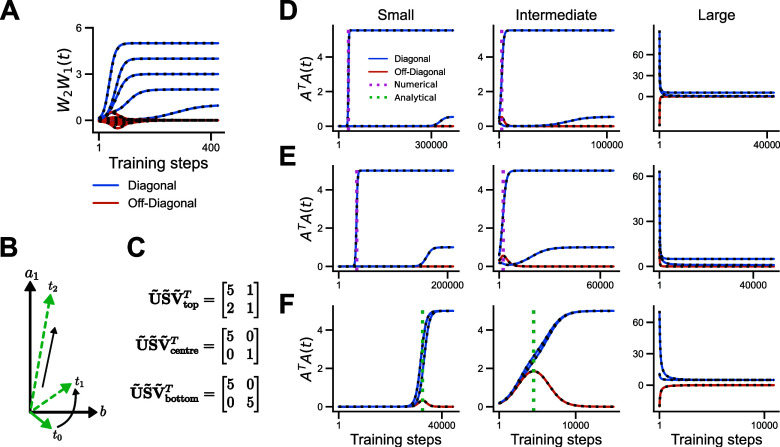
Decoupling dynamics. (A) Analytical (black dotted lines) and numerical (solid lines) of the temporal dynamics of the on- and off-diagonal elements of $\mathbf{A}^{\textrm T}\mathbf{A}$ in blue and red, respectively. (B) Schematic representation of the decoupling process. (C) Three target matrices with dense, unequal diagonal, and equal diagonal structure. (D)and (F) Decoupling dynamics for the top (D), middle (E), and bottom (F) tasks depicted in panel (C). Row F contains analytical predictions for the time of the peak of the off-diagonal (dashed green). The network is initialised as defined in appendix E with small, intermediate and large variance.

**Decoupling dynamics.** First, we track decoupling by considering the dynamics of the off-diagonal element *b*(*t*) (figures 4(D)–(F) red lines). At convergence, the off-diagonal element shrinks to zero as shown in appendix E. However, strikingly, *b*(*t*) can exhibit non-monotonic trajectories with transient peaks or valleys partway through the learning process. In particular, in appendix E we derive the time of the peak magnitude as $t_\textrm {peak} = \frac{\tau}{4s}\ln{\frac{s(s-a_1- a_2)}{a_1a_2-b(0)^2}}$ (figure 4(F) green dotted line), which coincides approximately with the time at which the on-diagonal element is half learned. If initialised from small random weights, the off-diagonal remains near-zero throughout learning, reminiscent of the silent alignment effect (Atanasov *et al* 2022). For large initialisations, no peak is observed and the dynamics are exponential. At intermediate initialisations, the maximum of the off-diagonal is reached before the singular mode is fully learned (appendix E). Intuitively, a particular input singular vector can initially project appreciably onto the wrong output singular vector, corresponding to initial misalignment. This is only revealed when this link is amplified, at which point corrective dynamics remove the counter-productive coupling, as schematised in figure 4(B). We report further measurements of decoupling in appendix E.

**Effect of initialisation variance**. Next, we revisit the impact of initialisation scale for the on-diagonal dynamics. As shown in figures 4(D)–(F), as the initialisation variance grows the learning dynamics change from sigmoidal to exponential, possibly displaying more complex behaviour at intermediate variance (appendix E). In this simple setting we can analyse this transition in detail. Taking $s_1 = s_2 = s$ as in figure 4(F) and $|a_1(0)|,|a_2(0)|,|b(0)|\ll 1$, we recover a sigmoidal trajectory,

\begin{equation*} a_1\left(t\right) = \frac{sa_1\left(0\right)}{e^{\frac{-2st}{\tau}}\left[s-a_1\left(0\right)-a_2\left(0\right)\right]+ a_1\left(0\right) +a_2\left(0\right)}, \end{equation*}

while for $|a_1(0)|,|a_2(0)|,|b(0)| \gg 0$ the dynamics of the on-diagonal element *a*_1_ is close to exponential (figures 4(D)–(F) left and right columns). We examine larger networks in appendix E.

## 6. Applications

The solutions derived in sections 3 and 5 provide tools to examine the impact of prior knowledge on dynamics in deep linear networks. So far we have traced general features of the behaviour of these solutions. In this section, we use this toolkit to develop accounts of several specific phenomena.

**Figure 5.**
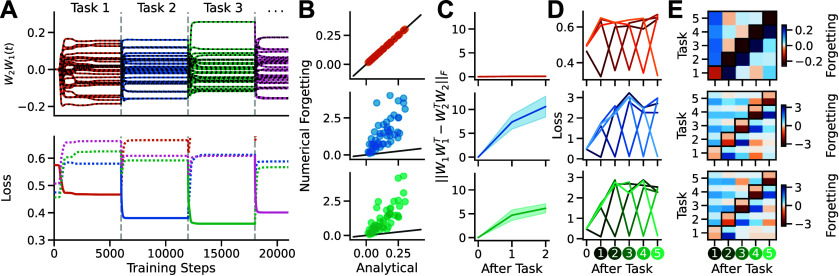
Continual learning. (A) Top: network training from small zero-balanced weights on a sequence of tasks (coloured lines show simulation and black dotted lines analytical results). Bottom: evaluation loss for tasks of the sequence (dotted) while training on the current task (solid). As the network function is optimised on the current task, the loss of other tasks increases. (B) Comparison of the numerical and analytical amount of catastrophic forgetting on a first task after training on a second task for *n* = 50 linear (red), tanh (blue) and ReLU (green) networks. (C) Weight alignment before and after training on a sequence of two tasks for *n* = 50 networks in linear (red), tanh (blue) and ReLU (green) networks. Shaded area shows $\pm \mathrm{std}$. (D) Evaluation loss for each of 5 tasks during training a linear (red), tanh (blue) and ReLU (green) network. (E) Same data es in (D) but evaluated as relative change (i.e. amount of catastrophic forgetting). The top half of each square shows the pre-computed analytical amount of forgetting and the bottom half the numerical value.

**Continual Learning**. Continual learning (see Parisi *et al* 2019 for a review) and the pathology of catastrophic forgetting have long been a challenge for neural network models (McCloskey and Cohen 1989, Ratcliff 1990, French 1999). A variety of theoretical work has investigated aspects of continual learning (Tripuraneni *et al* 2020, Asanuma *et al* 2021, Doan *et al* 2021, Lee *et al* 2021, Shachaf *et al* 2021). In this setting, starting from an initial set of weights, a network is trained on a sequence of tasks with respective input-output correlations $\mathcal{T}_1 = \mathbf{\tilde{\Sigma}}^{yx}_1, \mathcal{T}_2 = \mathbf{\tilde{\Sigma}}^{yx}_2, \mathcal{T}_3 = \mathbf{\tilde{\Sigma}}^{yx}_3, {\ldots}$. As shown in figure 5(A), our dynamics immediately enable exact solutions for the full continual learning process, whereby the final state after training on one task becomes the initial network state for the next task. These solutions thus reveal the exact time course of forgetting for arbitrary sequences of tasks.

Training on later tasks can overwrite previously learned knowledge, a phenomenon known as catastrophic forgetting (McCloskey and Cohen 1989, Ratcliff 1990, French 1999). From theorem 3.2 it follows that from any arbitrary zero-balanced initialisation 2.3, the network converges to the global optimum such that the initialisation is completely overwritten and forgetting is truly catastrophic. In particular, the loss of any other task $\mathcal{T}_ i$ after training to convergence on task $\mathcal{T}_j$ is $\mathcal{L}_ i(\mathcal{T}_j) = 1/2||\mathbf{\tilde{\Sigma}}^{yx}_j-\mathbf{\tilde{\Sigma}}^{yx}_\textrm i||_F^2 +c$, where *c* is a constant that only depends on training data of task $\mathcal{T}_ i$ (appendix F). As a consequence, the amount of forgetting, i.e. the relative change of loss, is fully determined by the similarity structure of the tasks and thus can be fully determined for a sequence of tasks before the onset of training (figures 5(B) and (E), appendix F). For example, the amount of catastrophic forgetting in task $\mathcal{T}_a$, when training on task $\mathcal{T}_c$ after having trained the network on task $\mathcal{T}_b$ is $\mathcal{L}_a(\mathcal{T}_c) - \mathcal{L}_a(\mathcal{T}_b)$. As expected, our results depend on our linear setting and tanh or ReLU nonlinearities can show different behaviour, typically increasing the amount of forgetting (figures 5(B), (D) and (E)). Further, in nonlinear networks, weights become rapidly unbalanced and forgetting values that are calculated before the onset of training do not predict the actual outcome (figures 5(B)–(E)). In summary, our results link exact learning dynamics with catastrophic forgetting and thus provide an analytical tool to study the mechanisms and potential counter measures underlying catastrophic forgetting.

**Figure 6.**
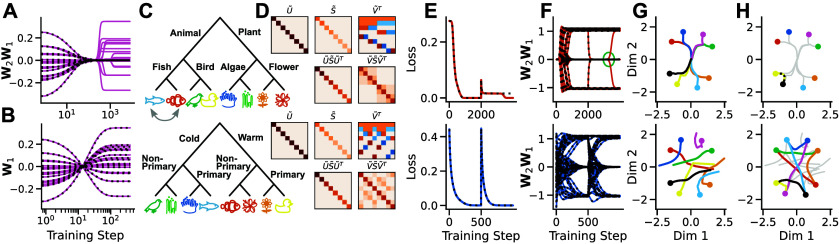
Reversal learning and revising structured knowledge. Scale of *x*-axis varies in top and bottom rows. (A) Analytical (black dotted) and numerical (solid) learning dynamics of a reversal learning task. The analytical solution gets stuck on a saddle point, whereas the numerical simulation escapes the saddle point and converges to the target. (B) In a shallow network, training on the same task as in A converges analytically (black dotted) and numerically (solid). (C) Semantic learning tasks. Revised living kingdom (top) and colour hierarchy (bottom). (D) SVD of the input-output correlation of the tasks and respective RSMs. (E) Analytical (black dotted) and simulation (solid) loss and (F) learning dynamics of first training on the living kingdom (figure 3(A)) and subsequently on the respective task in (C). The analytical solution fails for the revised animal kingdom as it gets stuck in a saddle point, while the simulation escapes the saddle (top, green circle). Initial training on the living kingdom task from large initial weights and subsequent training on the colour hierarchy have similar convergence times (bottom) (G) multidimensional scaling (MDS) of the network function for initial training on the living kingdom task from small (top) and large initial weights (bottom). Note how despite the seemingly chaotic learning dynamics when starting form large initial weights, both simulations learn the same representation. (H) MDS of subsequent training on the respective task in (C).

**Reversal learning**. During reversal learning, pre-existing knowledge has to be relearned, overcoming a previously learned relationship between inputs and outputs. For example, reversal learning occurs when items of a class are mislabelled and later corrected. We show analytically, that reversal learning in fact does not succeed in deep linear networks (appendix G). The pre-existing knowledge lies exactly on the separatrix of a saddle point causing the learning dynamics to converge to zero (figure 6(A)). In contrast, the learning still succeeds numerically, as any noise will perturb the dynamics off the saddle point, allowing learning to proceed (figure 6(A)). However, the dynamics still slow in the vicinity of the saddle point, providing a theoretical explanation for catastrophic slowing in deep linear networks (Lee *et al* 2022). We note that the analytical solution requires an adaptation of theorem 3.1, as **B** is generally not invertible in the case of reversal learning (appendix G). Further, as is revealed by the exact learning dynamics (appendix G), shallow networks do succeed without exhibiting catastrophic slowing during reversal learning (figure 6(B)).

**Revising structured knowledge**. Knowledge is often organised within an underlying, shared structure, of which many can be learned and represented in deep linear networks (Saxe *et al* 2019). For example, spatial locations can be related to each other using the same cardinal directions, or varying semantic knowledge can be organised using the same hierarchical tree. Here, we investigate if deep linear networks benefit from shared underlying structure. To this end, a network is first trained on the three-level hierarchical tree of section 4 (eight items of the living kingdom, each with a set of eight associated features), and subsequently trained on a revised version of the hierarchy. The revised task varies the relation of inputs and outputs while keeping the same underlying tree structure. If the revision involves swapping two neighbouring nodes on any level of the hierarchy, e.g. the identity of the two fish on the lowest level of the hierarchy (figure 6(C), top), the task is identical to reversal learning, leading to catastrophically slowed dynamics (figures 6(E) and (F), top). When training the network on a new hierarchical tree with identical items but a new set of features, like a colour hierarchy (figure 6(C), bottom), there is no speed advantage in comparison to a random initialisation with similar initial variance (figures 6(E) and (F), bottom). Importantly, from theorem 3.2 it follows, that the learning process can be sped up significantly by initialising from large zero-balanced weights, while converging to a global minimum with identical generalisation properties as when training from small weights (figures 6(G) and (H). In summary, having incorporated structured knowledge before revision does not speed up or even slows down learning in comparison to learning from random zero-balanced weights. Notably, that is despite the tasks’ structure being almost identical (figures 3(B) and 6(D).

## 7. Discussion

We derive exact solutions to the dynamics of learning with rich prior knowledge in a tractable model class: deep linear networks. While our results broaden the class of two-layer linear network problems that can be described analytically, they remain limited and rely on a set of assumptions (2.1)–(2.4). In particular, weakening the requirement that the input covariance be white and the weights be zero-balanced would enable analysis of the impact of initialisation on internal representations. Nevertheless, these solutions reveal several insights into network behaviour. We show that there exists a large set of initial values, namely zero-balanced weights 2.3, which lead to task-specific representations; and that large initialisations lead to exponential rather than sigmoidal learning curves. We hope our results provide a mathematical toolkit that illuminates the complex impact of prior knowledge on deep learning dynamics.

## Figures and Tables

**Figure 7.**
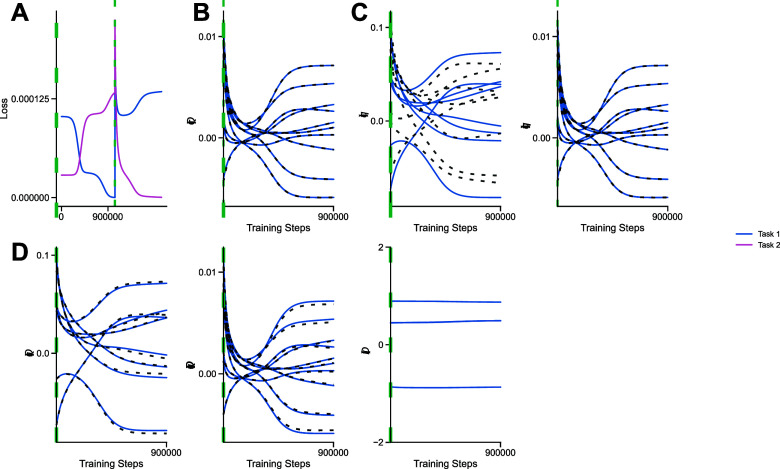
(A) Loss under gradient descent learning two random input-output correlation task with learning rate $\eta = 0,001$ up to precision $1 \times 10^{-7}$. The green dotted line marks the time at which the target is switched from task 1 to task 2. (B) Numerical (coloured line) and analytical (black dotted line) temporal dynamics of $\mathbf{Q}\mathbf{Q}^{\textrm T}(t)$ as given by equation (159). (C) Numerical (coloured line) and analytical (black dotted line) temporal dynamics of $\mathbf{q}(t)$ and $\mathbf{q}(t)\mathbf{q}(t)^{\textrm T}$ (158) (D) Temporal dynamics of $\mathbf{D}(t)$. Numerical (coloured line) and analytical (black dotted line) temporal dynamics of $\mathbf{Q}_d (t)\mathbf{Q}_d (t)^{\textrm T}$ and $\mathbf{Q}_d (t)$ as given by equation (157) where (D) was computed numerically.

**Figure 8.**
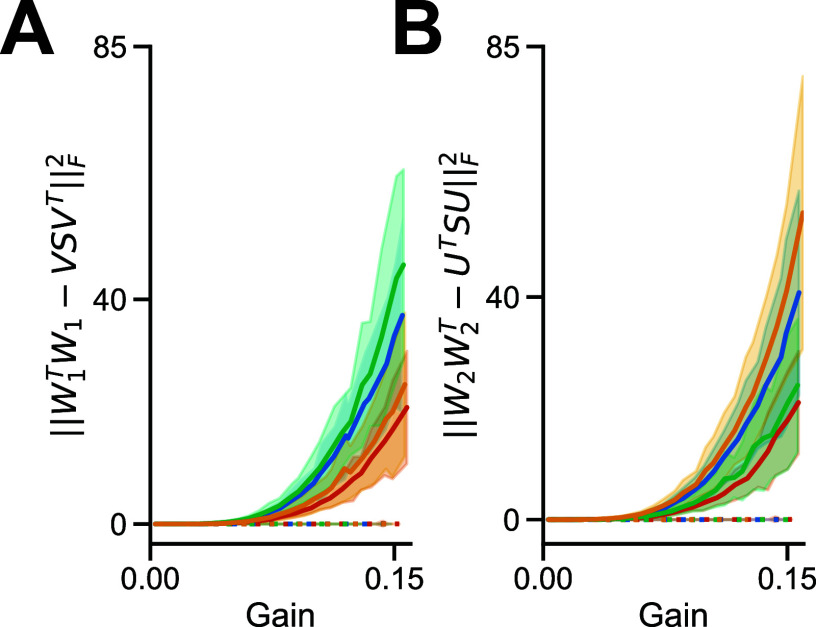
(A) and (B) Mean and standard deviation on the error on the internal representation error defined as in section D for the learning the living kingdom task (figure 6(A)), a random $7\times7$ matrix (blue), a random $5\times7$ matrix (yellow), a $7\times 5$ matrix (green), a $8\times 8$ matrix (red). All the task ran were ran with learning rate *η* = 0.001 enforcing initial zero-balanced weights 2.3 (dotted line) and breaking the assumption of zero-balanced initial weights 2.3 (line). $N_\textrm h = 10$ for all networks.

**Figure 9.**
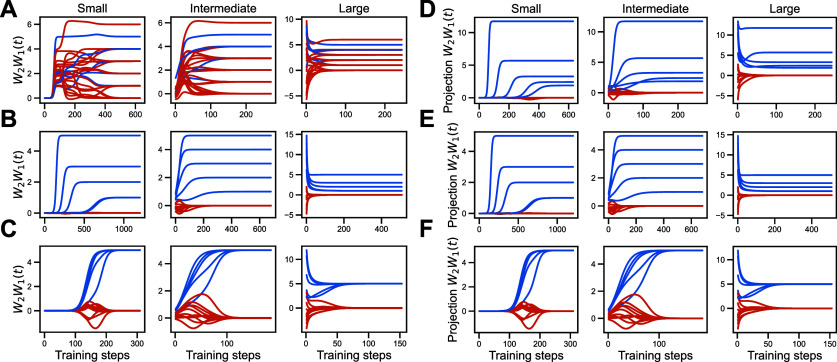
(A)–(C) Network function dynamics (Diagonal elements: blue, Off-diagonal elements: red) learning with learning rate *η* = 0.01 on the target $5\times5$ diagonal matrices shown in equation (198). The network was initialised as defined in section E with Small ($\sigma = 1 \times 10^{-6}$), Intermediate (*σ* = 0.1) and Large (*σ* = 2) variance, and hidden layer size $N_\textrm h = 10$. (A), Dense. (B), Diagonal. (C), Equal diagonal. (D)–(F). Corresponding numerical temporal dynamics of the projection of the network function on- and off-diagonal elements into the singular-basis of the initialisation. Equivalently, the temporal dynamics of the elements of $\mathbf{A}\mathbf{A}^{\textrm T}$ bottom left quadrant. (D), Dense. (E), Diagonal. (F), Equal diagonal.

## Appendix A Fukumizu approach

For completeness, we reproduce the derivation from Fukumizu (1998) of equation (5). We consider the learning setting describe in section 2. Under the assumptions of equal input-output dimensions 2.1, whitened inputs 2.2 and zero-balanced weights 2.3, the weights dynamics yield

\begin{align*} &amp;\tau {\frac{\textrm{d}}{\textrm dt}}\mathbf{W}_1 = \mathbf{W}_2^{\textrm T}\left(\boldsymbol{\tilde{\Sigma}}^{yx}-\mathbf{W}_2\mathbf{W}_1\boldsymbol{\tilde{\Sigma}}^{xx}\right), \end{align*}

\begin{align*} &amp;\tau {\frac{\textrm{d}}{\textrm dt}}\mathbf{W}_2 = \left(\boldsymbol{\tilde{\Sigma}}^{yx}-\mathbf{W}_2\mathbf{W}_1\boldsymbol{\tilde{\Sigma}}^{xx}\right)\mathbf{W}_1^{\textrm T}. \end{align*}

Under the assumption of whitened inputs 2.2, the dynamics simplify to

\begin{align*} \tau {\frac{\textrm{d}}{\textrm dt}}\mathbf{W}_1 = \mathbf{W}_2^{\textrm T}\left(\boldsymbol{\tilde{\Sigma}}^{yx}-\mathbf{W}_2\mathbf{W}_1\right), \end{align*}

\begin{align*} \tau {\frac{\textrm{d}}{\textrm dt}}\mathbf{W}_2 = \left(\boldsymbol{\tilde{\Sigma}}^{yx}-\mathbf{W}_2\mathbf{W}_1\right)\mathbf{W}_1^{\textrm T}. \end{align*}

We introduce the variables

\begin{equation*} \mathbf{Q} = \begin{bmatrix} \mathbf{W}_1^{\textrm T} \\ \mathbf{W}_2 \end{bmatrix} \ \text{and} \ \mathbf{Q}\mathbf{Q}^{\textrm T} = \begin{bmatrix} \mathbf{W}_1^{\textrm T}\mathbf{W}_1 &amp; \mathbf{W}_1^{\textrm T}\mathbf{W}_2^{\textrm T} \\[3pt] \mathbf{W}_2\mathbf{W}_1 &amp; \mathbf{W}_2\mathbf{W}_2^{\textrm T} \end{bmatrix} \text{.} \end{equation*}

We compute the time derivative

\begin{equation*} \tau\frac{\textrm{d}}{\textrm dt}\left(\mathbf{Q}\mathbf{Q}^{\textrm T}\right) = \tau \begin{bmatrix} \frac{\textrm d\mathbf{W}_1^{\textrm T}}{\textrm dt}\mathbf{W}_1+ \mathbf{W}_1^{\textrm T} \frac{\textrm d\mathbf{W}_1}{d t} &amp; \frac{\textrm d\mathbf{W}_1^{\textrm T}}{\textrm dt}\mathbf{W}_2^{\textrm T}+ \mathbf{W}_1^{\textrm T} \frac{\textrm d\mathbf{W}_2^{\textrm T}}{\textrm dt} \\[4pt] \frac{\textrm d\mathbf{W}_2}{\textrm dt}\mathbf{W}_1+ \mathbf{W}_2 \frac{\textrm d\mathbf{W}_1}{\textrm dt} &amp; \frac{\textrm d\mathbf{W}_2}{\textrm dt}\mathbf{W}_2^{\textrm T}+ \mathbf{W}_2 \frac{\textrm d\mathbf{W}_2^{\textrm T}}{\textrm dt} \end{bmatrix} \text{.} \end{equation*}

Using equations (18) and (19) we compute the four quadrant separately giving

\begin{align*} &amp;\tau\left( \frac{\textrm d\mathbf{W}_1^{\textrm T}}{\textrm dt}\mathbf{W}_1+ \mathbf{W}_1^{\textrm T} \frac{\textrm d\mathbf{W}_1}{\textrm dt}\right) \end{align*}

\begin{align*} &amp; \quad = \left(\boldsymbol{\tilde{\Sigma}}^{yx}-\mathbf{W}_2\mathbf{W}_1\right)^{\textrm T} \mathbf{W}_2\mathbf{W}_1 + \mathbf{W}_1^{\textrm T} \mathbf{W}_2^{\textrm T}\left(\boldsymbol{\tilde{\Sigma}}^{yx}-\mathbf{W}_2\mathbf{W}_1\right) \end{align*}

\begin{align*} &amp; \quad = \left(\boldsymbol{\tilde{\Sigma}}^{yx}\right)^{\textrm T} \mathbf{W}_2\mathbf{W}_1 + \mathbf{W}_1^{\textrm T}\mathbf{W}_2^{\textrm T}\boldsymbol{\tilde{\Sigma}}^{yx}- \mathbf{W}_1^{\textrm T}\mathbf{W}_2^{\textrm T}\mathbf{W}_2\mathbf{W}_1 -\left(\mathbf{W}_2\mathbf{W}_1\right)^{\textrm T}\mathbf{W}_2\mathbf{W}_1 \end{align*}

\begin{align*} &amp; \quad = \left(\boldsymbol{\tilde{\Sigma}}^{yx}\right)^{\textrm T} \mathbf{W}_2\mathbf{W}_1 + \mathbf{W}_1^{\textrm T}\mathbf{W}_2^{\textrm T}\boldsymbol{\tilde{\Sigma}}^{yx}- \mathbf{W}_1^{\textrm T}\mathbf{W}_2^{\textrm T}\mathbf{W}_2\mathbf{W}_1- \mathbf{W}_1^{\textrm T}\mathbf{W}_1\mathbf{W}_1^{\textrm T}\mathbf{W}_1, \end{align*}

\begin{align*} &amp; \tau \left(\frac{\textrm d\mathbf{W}_1^{\textrm T}}{\textrm dt}\mathbf{W}_2^{\textrm T}+ \mathbf{W}_1^{\textrm T} \frac{\textrm d\mathbf{W}_2^{\textrm T}}{\textrm dt} \right) \end{align*}

\begin{align*} &amp; \quad = \left(\boldsymbol{\tilde{\Sigma}}^{yx}-\mathbf{W}_2\mathbf{W}_1\right)^{\textrm T} \mathbf{W}_2\mathbf{W}_2^{\textrm T}+\mathbf{W}_1^{\textrm T}\mathbf{W}_1 \left(\boldsymbol{\tilde{\Sigma}}^{yx}-\mathbf{W}_2\mathbf{W}_1\right)^{\textrm T} \end{align*}

\begin{align*} &amp; \quad = \left(\boldsymbol{\tilde{\Sigma}}^{yx}\right)^{\textrm T}\mathbf{W}_2\mathbf{W}_2^{\textrm T}+\mathbf{W}_1^{\textrm T}\mathbf{W}_1\left(\boldsymbol{\tilde{\Sigma}}^{yx}\right)^{\textrm T}-\mathbf{W}_1^{\textrm T}\mathbf{W}_1\left(\mathbf{W}_2\mathbf{W}_1\right)^{\textrm T}-\left(\mathbf{W}_2\mathbf{W}_1\right)^{\textrm T} \mathbf{W}_2\mathbf{W}_2^{\textrm T}, \end{align*}

\begin{align*} &amp; \quad = \left(\boldsymbol{\tilde{\Sigma}}^{yx}\right)^{\textrm T}\mathbf{W}_2\mathbf{W}_2^{\textrm T}+\mathbf{W}_1^{\textrm T}\mathbf{W}_1\left(\boldsymbol{\tilde{\Sigma}}^{yx}\right)^{\textrm T}-\mathbf{W}_1^{\textrm T}\mathbf{W}_1\mathbf{W}_1^{\textrm T}\mathbf{W}_2^{\textrm T}-\mathbf{W}_1^{\textrm T}\mathbf{W}_2^{\textrm T} \mathbf{W}_2\mathbf{W}_2^{\textrm T}, \end{align*}

\begin{align*} &amp; \tau \left( \frac{\textrm d\mathbf{W}_2}{\textrm dt}\mathbf{W}_1+ \mathbf{W}_2 \frac{\textrm d\mathbf{W}_1}{\textrm dt} \right) \end{align*}

\begin{align*} &amp; \quad = \left(\boldsymbol{\tilde{\Sigma}}^{yx}-\mathbf{W}_2\mathbf{W}_1\right)\mathbf{W}_1^{\textrm T}\mathbf{W}_1+ \mathbf{W}_2\mathbf{W}_2^{\textrm T}\left(\boldsymbol{\tilde{\Sigma}}^{yx}-\mathbf{W}_2\mathbf{W}_1\right) \end{align*}

\begin{align*} &amp; \quad = \boldsymbol{\tilde{\Sigma}}^{yx}\mathbf{W}_1^{\textrm T}\mathbf{W}_1+ \mathbf{W}_2\mathbf{W}_2^{\textrm T}\boldsymbol{\tilde{\Sigma}}^{yx}-\mathbf{W}_2\mathbf{W}_2^{\textrm T}\mathbf{W}_2\mathbf{W}_1-\mathbf{W}_2\mathbf{W}_1\mathbf{W}_1^{\textrm T}\mathbf{W}_1, \end{align*}

\begin{align*} &amp;\tau \left(\frac{\textrm d\mathbf{W}_2}{\textrm dt}\mathbf{W}_2^{\textrm T}+ \mathbf{W}_2 \frac{\textrm d\mathbf{W}_2^{\textrm T}}{\textrm dt}\right) \end{align*}

\begin{align*} &amp; \quad = \left(\boldsymbol{\tilde{\Sigma}}^{yx}-\mathbf{W}_2\mathbf{W}_1\right)\mathbf{W}_1^{\textrm T}\mathbf{W}_2^{\textrm T}+ \mathbf{W}_2 \mathbf{W}_1 \left(\boldsymbol{\tilde{\Sigma}}^{yx}-\mathbf{W}_2\mathbf{W}_1\right)^{\textrm T} \end{align*}

\begin{align*} &amp; \quad = \boldsymbol{\tilde{\Sigma}}^{yx}\mathbf{W}_1^{\textrm T}\mathbf{W}_2^{\textrm T}+ \mathbf{W}_2\mathbf{W}_1\left(\boldsymbol{\tilde{\Sigma}}^{yx}\right)^{\textrm T} -\mathbf{W}_2\mathbf{W}_1\mathbf{W}_1^{\textrm T}\mathbf{W}_2^{\textrm T} -\mathbf{W}_2\mathbf{W}_1\left(\mathbf{W}_2\mathbf{W}_1\right)^{\textrm T} \end{align*}

\begin{align*} &amp; \quad = \boldsymbol{\tilde{\Sigma}}^{yx}\mathbf{W}_1^{\textrm T}\mathbf{W}_2^{\textrm T}+ \mathbf{W}_2\mathbf{W}_1\left(\boldsymbol{\tilde{\Sigma}}^{yx}\right)^{\textrm T} -\mathbf{W}_2\mathbf{W}_1\mathbf{W}_1^{\textrm T}\mathbf{W}_2^{\textrm T} -\mathbf{W}_2\mathbf{W}_1\mathbf{W}_1^{\textrm T}\mathbf{W}_2^{\textrm T} \end{align*}

\begin{align*} &amp; \quad = \boldsymbol{\tilde{\Sigma}}^{yx}\mathbf{W}_1^{\textrm T}\mathbf{W}_2^{\textrm T}+ \mathbf{W}_2\mathbf{W}_1\left(\boldsymbol{\tilde{\Sigma}}^{yx}\right)^{\textrm T} -\mathbf{W}_2\mathbf{W}_1\mathbf{W}_1^{\textrm T}\mathbf{W}_2^{\textrm T} -\mathbf{W}_2\mathbf{W}_2^{\textrm T}\mathbf{W}_2\mathbf{W}_2^{\textrm T}, \end{align*}

where we have used the assumption of zero-balanced weights 2.3 to simplify equations (25) and (37).

Defining

\begin{equation*} \mathbf{F} = \begin{bmatrix} 0 &amp; \left(\boldsymbol{\tilde{\Sigma}}^{yx}\right)^{\textrm T}\\ \boldsymbol{\tilde{\Sigma}}^{yx} &amp; 0 \end{bmatrix}, \end{equation*}

the gradient flow dynamics of $\mathbf{Q}\mathbf{Q}^{\textrm T}$(t) can be written as a differential matrix Riccati equation

\begin{align*} \tau\frac{\textrm{d}}{\textrm dt}\left(\mathbf{Q}\mathbf{Q}^{\textrm T}\right) = \mathbf{F}\mathbf{Q}\mathbf{Q}^{\textrm T}+\mathbf{Q}\mathbf{Q}^{\textrm T}\mathbf{F} -\left(\mathbf{Q}\mathbf{Q}^{\textrm T}\right)^2. \end{align*}

We write $\tau\frac{\textrm{d}}{\textrm dt}(\mathbf{Q}\mathbf{Q}^{\textrm T})$ for completeness

\begin{align*} &amp;\tau\frac{\textrm{d}}{\textrm dt}\left(\mathbf{Q}\mathbf{Q}^{\textrm T}\right) \nonumber\\ &amp;\quad = \begin{bmatrix} 0 &amp; \left(\boldsymbol{\tilde{\Sigma}}^{yx}\right)^{\textrm T}\\ \boldsymbol{\tilde{\Sigma}}^{yx} &amp; 0 \end{bmatrix}\begin{bmatrix} \mathbf{W}_1^{\textrm T}\mathbf{W}_1 &amp; \mathbf{W}_1^{\textrm T}\mathbf{W}_2^{\textrm T} \\[4pt] \mathbf{W}_2\mathbf{W}_1 &amp; \mathbf{W}_2\mathbf{W}_2^{\textrm T} \end{bmatrix} \nonumber\\ &amp; \qquad + \begin{bmatrix} \mathbf{W}_1^{\textrm T}\mathbf{W}_1 &amp; \mathbf{W}_1^{\textrm T}\mathbf{W}_2^{\textrm T} \\[4pt] \mathbf{W}_2\mathbf{W}_1 &amp; \mathbf{W}_2\mathbf{W}_2^{\textrm T} \end{bmatrix}^{\textrm T} \begin{bmatrix} 0 &amp; \left(\boldsymbol{\tilde{\Sigma}}^{yx}\right)^{\textrm T}\\ \boldsymbol{\tilde{\Sigma}}^{yx} &amp; 0 \end{bmatrix} -\begin{bmatrix} \mathbf{W}_1^{\textrm T}\mathbf{W}_1 &amp; \mathbf{W}_1^{\textrm T}\mathbf{W}_2^{\textrm T} \\[4pt] \mathbf{W}_2\mathbf{W}_1 &amp; \mathbf{W}_2\mathbf{W}_2^{\textrm T} \end{bmatrix} ^2 \end{align*}

\begin{align*} &amp;\quad = \begin{bmatrix} 0 &amp; \left(\boldsymbol{\tilde{\Sigma}}^{yx}\right)^{\textrm T}\\ \boldsymbol{\tilde{\Sigma}}^{yx} &amp; 0 \end{bmatrix}\begin{bmatrix} \mathbf{W}_1^{\textrm T}\mathbf{W}_1 &amp; \mathbf{W}_1^{\textrm T}\mathbf{W}_2^{\textrm T} \\[4pt] \mathbf{W}_2\mathbf{W}_1 &amp; \mathbf{W}_2\mathbf{W}_2^{\textrm T} \end{bmatrix} \nonumber\\ &amp; \qquad + \begin{bmatrix} \mathbf{W}_1^{\textrm T}\mathbf{W}_1 &amp; \mathbf{W}_1^{\textrm T}\mathbf{W}_2^{\textrm T} \\[4pt] \mathbf{W}_2\mathbf{W}_1 &amp; \mathbf{W}_2\mathbf{W}_2^{\textrm T} \end{bmatrix} \begin{bmatrix} 0 &amp; \left(\boldsymbol{\tilde{\Sigma}}^{yx}\right)^{\textrm T}\\[4pt] \boldsymbol{\tilde{\Sigma}}^{yx} &amp; 0 \end{bmatrix} \nonumber\\ &amp; \qquad -\begin{bmatrix} \mathbf{W}_1^{\textrm T}\mathbf{W}_1 &amp; \mathbf{W}_1^{\textrm T}\mathbf{W}_2^{\textrm T} \\[4pt] \mathbf{W}_2\mathbf{W}_1 &amp; \mathbf{W}_2\mathbf{W}_2^{\textrm T} \end{bmatrix} \begin{bmatrix} \mathbf{W}_1^{\textrm T}\mathbf{W}_1 &amp; \mathbf{W}_1^{\textrm T}\mathbf{W}_2^{\textrm T} \\[4pt] \mathbf{W}_2\mathbf{W}_1 &amp; \mathbf{W}_2\mathbf{W}_2^{\textrm T} \end{bmatrix} \end{align*}

\begin{align*} &amp;\quad = \begin{bmatrix} \left(\boldsymbol{\tilde{\Sigma}}^{yx}\right)^{\textrm T}\mathbf{W}_2\mathbf{W}_1 &amp; \left(\boldsymbol{\tilde{\Sigma}}^{yx}\right)^{\textrm T} \mathbf{W}_2\mathbf{W}_2^{\textrm T}\\[4pt] \boldsymbol{\tilde{\Sigma}}^{yx} \mathbf{W}_1^{\textrm T}\mathbf{W}_1 &amp; \boldsymbol{\tilde{\Sigma}}^{yx}\mathbf{W}_1^{\textrm T}\mathbf{W}_2^{\textrm T} \end{bmatrix} \nonumber\\ &amp; \qquad + \begin{bmatrix} \mathbf{W}_1^{\textrm T}\mathbf{W}_2^{\textrm T} \boldsymbol{\tilde{\Sigma}}^{yx} &amp;\mathbf{W}_1^{\textrm T}\mathbf{W}_1 \left(\boldsymbol{\tilde{\Sigma}}^{yx} \right) ^{\textrm T}\\[4pt] \mathbf{W}_2\mathbf{W}_2^{\textrm T} \boldsymbol{\tilde{\Sigma}}^{yx} &amp; \mathbf{W}_2\mathbf{W}_1 \left(\boldsymbol{\tilde{\Sigma}}^{yx}\right)^{\textrm T} \end{bmatrix} \nonumber \nonumber\\ &amp; \qquad -\begin{bmatrix} \mathbf{W}_1^{\textrm T}\mathbf{W}_1 &amp; \mathbf{W}_1^{\textrm T}\mathbf{W}_2^{\textrm T} \\[4pt] \mathbf{W}_2\mathbf{W}_1 &amp; \mathbf{W}_2\mathbf{W}_2^{\textrm T} \end{bmatrix} \begin{bmatrix} \mathbf{W}_1^{\textrm T}\mathbf{W}_1 &amp; \mathbf{W}_1^{\textrm T}\mathbf{W}_2^{\textrm T} \\[4pt] \mathbf{W}_2\mathbf{W}_1 &amp; \mathbf{W}_2\mathbf{W}_2^{\textrm T} \end{bmatrix} \end{align*}

\begin{align*} &amp;\quad = \begin{bmatrix} \left(\boldsymbol{\tilde{\Sigma}}^{yx}\right)^{\textrm T}\mathbf{W}_2\mathbf{W}_1 &amp; \left(\boldsymbol{\tilde{\Sigma}}^{yx}\right)^{\textrm T} \mathbf{W}_2\mathbf{W}_2^{\textrm T}\\[4pt] \boldsymbol{\tilde{\Sigma}}^{yx} \mathbf{W}_1^{\textrm T}\mathbf{W}_1 &amp; \boldsymbol{\tilde{\Sigma}}^{yx}\mathbf{W}_1^{\textrm T}\mathbf{W}_2^{\textrm T} \end{bmatrix} \nonumber\\ &amp; \qquad + \begin{bmatrix} \mathbf{W}_1^{\textrm T}\mathbf{W}_2^{\textrm T} \boldsymbol{\tilde{\Sigma}}^{yx} &amp;\mathbf{W}_1^{\textrm T}\mathbf{W}_1 \left(\boldsymbol{\tilde{\Sigma}}^{yx} \right) ^{\textrm T}\\[4pt] \mathbf{W}_2\mathbf{W}_2^{\textrm T} \boldsymbol{\tilde{\Sigma}}^{yx} &amp; \mathbf{W}_2\mathbf{W}_1 \left(\boldsymbol{\tilde{\Sigma}}^{yx}\right)^{\textrm T} \end{bmatrix} \nonumber \\[4pt] &amp;\qquad -\begin{bmatrix} \mathbf{W}_1^{\textrm T}\mathbf{W}_1\mathbf{W}_1^{\textrm T}\mathbf{W}_1+\mathbf{W}_1^{\textrm T}\mathbf{W}_2^{\textrm T} \mathbf{W}_2\mathbf{W}_1 &amp; \mathbf{W}_1^{\textrm T}\mathbf{W}_1 \mathbf{W}_1^{\textrm T}\mathbf{W}_2^{\textrm T} + \mathbf{W}_1^{\textrm T}\mathbf{W}_2^{\textrm T} \mathbf{W}_2\mathbf{W}_2^{\textrm T}\\[6pt] \mathbf{W}_2\mathbf{W}_1 \mathbf{W}_1^{\textrm T}\mathbf{W}_1+ \mathbf{W}_2\mathbf{W}_2^{\textrm T} \mathbf{W}_2\mathbf{W}_1 &amp; \mathbf{W}_2\mathbf{W}_1 \mathbf{W}_1^{\textrm T}\mathbf{W}_2^{\textrm T} + \mathbf{W}_2\mathbf{W}_2^{\textrm T} \mathbf{W}_2\mathbf{W}_2^{\textrm T} \end{bmatrix} \end{align*}

\begin{align*} &amp;\qquad = \begin{bmatrix} \left(\boldsymbol{\tilde{\Sigma}}^{yx}\right)^{\textrm T} \mathbf{W}_2\mathbf{W}_1 + \mathbf{W}_1^{\textrm T}\mathbf{W}_2^{\textrm T}\boldsymbol{\tilde{\Sigma}}^{yx}\\- \mathbf{W}_1^{\textrm T}\mathbf{W}_2^{\textrm T}\mathbf{W}_2\mathbf{W}_1 -\mathbf{W}_1^{\textrm T}\mathbf{W}_1\mathbf{W}_1^{\textrm T}\mathbf{W}_1 &amp; \left(\boldsymbol{\tilde{\Sigma}}^{yx}\right)^{\textrm T}\mathbf{W}_2\mathbf{W}_2^{\textrm T}+\mathbf{W}_1^{\textrm T}\mathbf{W}_1 \left(\boldsymbol{\tilde{\Sigma}}^{yx}\right)^{\textrm T}\\ &amp;-\mathbf{W}_1^{\textrm T}\mathbf{W}_1\mathbf{W}_1^{\textrm T}\mathbf{W}_2^{\textrm T}-\mathbf{W}_1^{\textrm T}\mathbf{W}_2^{\textrm T} \mathbf{W}_2\mathbf{W}_2^{\textrm T}\\ \boldsymbol{\tilde{\Sigma}}^{yx}\mathbf{W}_1^{\textrm T}\mathbf{W}_1+ \mathbf{W}_2\mathbf{W}_2^{\textrm T}\boldsymbol{\tilde{\Sigma}}^{yx}\\-\mathbf{W}_2\mathbf{W}_2^{\textrm T}\mathbf{W}_2\mathbf{W}_1-\mathbf{W}_2\mathbf{W}_1\mathbf{W}_1^{\textrm T}\mathbf{W}_1 &amp; \boldsymbol{\tilde{\Sigma}}^{yx}\mathbf{W}_1^{\textrm T}\mathbf{W}_2^{\textrm T}+ \mathbf{W}_2\mathbf{W}_1\left(\boldsymbol{\tilde{\Sigma}}^{yx}\right)^{\textrm T}\\ &amp;-\mathbf{W}_2\mathbf{W}_1\mathbf{W}_1^{\textrm T}\mathbf{W}_2^{\textrm T} -\mathbf{W}_2\mathbf{W}_2^{\textrm T}\mathbf{W}_2\mathbf{W}_2^{\textrm T} \\ \end{bmatrix} \end{align*}

$\square$

The four quadrant of (44) are equivalent to equations (25), (29), (32) and (37) respectively.

Assuming that $\mathbf{Q}(0)$ is full rank, the continuous differential equation (39) has a unique solution for all $t\unicode{x2A7E}0$

\begin{align*} \mathbf{Q}\mathbf{Q}^{\textrm T}\left(t\right) = e^{\mathbf{F}\frac{t}{\tau}}\mathbf{Q}\left(0\right)\left[\mathbf{I} + \frac{1}{2}\mathbf{Q}\left(0\right)^{\textrm T}\left(e^{\mathbf{F}\frac{t}{\tau}}\mathbf{F}^{-1}e^{\mathbf{F}\frac{t}{\tau}} - \mathbf{F}^{-1} \right) \mathbf{Q}\left(0\right)\right]^{-1} \mathbf{Q}\left(0\right)^Te^{\mathbf{F}\frac{t}{\tau}} \text{.} \end{align*}

## Appendix B Network’s internal representations

### B.1. Representational similarity analysis

The task-relevant representational similarity matrix (Kriegeskorte *et al* 2008) of the hidden layer, calculated from the inputs $\mathbf{H} = \mathbf{W}_1\mathbf{X}$ is

\begin{align*} \mathrm{RSM}_I\left(t\right) &amp; = \mathbf{H}^{\textrm T}\left(t\right)\mathbf{H}\left(t\right) \end{align*}

\begin{align*} &amp; = \left(\mathbf{W}_1\left(t\right)\mathbf{X}\right)^{\textrm T}\mathbf{W}_1\left(t\right)\mathbf{X} \end{align*}

\begin{align*} &amp; = \mathbf{X}^{\textrm T}\left(\mathbf{W}_1^{\textrm T}\mathbf{W}_1\right)\left(t\right)\mathbf{X}. \end{align*}

Similarly, the representational similarity matrix of the hidden layer, calculated from the outputs ${\tilde{\mathbf{H}}} = \mathbf{W}_2^+ Y$, where + denotes the pseudoinverse, is

\begin{align*} \mathrm{RSM}_O\left(t\right) &amp; = {\tilde{\mathbf{H}}}^{\textrm T}\left(t\right){\tilde{\mathbf{H}}}\left(t\right) \end{align*}

\begin{align*} &amp; = \left(\mathbf{W}_2^+\left(t\right)Y\right)^{\textrm T}\mathbf{W}_2^+\left(t\right) Y \end{align*}

\begin{align*} &amp; = Y^{\textrm T}\left(\mathbf{W}_2\mathbf{W}_2^{\textrm T}\left(t\right)\right)^+ Y. \end{align*}

### B.2. Finite-width neural tangent kernel

In the following, we derive the finite-width neural tangent kernel (Jacot *et al* 2018) for a two-layer linear network. Starting with the network function at time *t*

\begin{equation*} F_t\left(\mathbf{X}\right) = \mathbf{W}_2\mathbf{W}_1\mathbf{X}, \end{equation*}

the discrete time gradient descent dynamics of the next time step yields

\begin{align*} F_{t+1}\left(\mathbf{X}\right) &amp; = \left(\mathbf{W}_2-\eta\frac{\partial\mathcal{L}}{\partial\mathbf{W}_2}\right) \left(\mathbf{W}_1 - \eta\frac{\partial\mathcal{L}}{\partial\mathbf{W}_1}\right)\mathbf{X} \end{align*}

\begin{align*} &amp; = \mathbf{W}_2\mathbf{W}_1\mathbf{X} - \eta \left(\mathbf{W}_2 \frac{\partial\mathcal{L}}{\partial\mathbf{W}_1} + \frac{\partial\mathcal{L}}{\partial\mathbf{W}_2}\mathbf{W}_1 - \eta \frac{\partial\mathcal{L}}{\partial\mathbf{W}_2} \frac{\partial\mathcal{L}}{\partial\mathbf{W}_1} \right) \mathbf{X}. \end{align*}

The network function’s gradient flow can then be derived as

\begin{align*} \frac{F_{t+1}\left(\mathbf{X}\right) - F_{t}\left(\mathbf{X}\right)}{\eta} &amp; = - \left(\mathbf{W}_2 \frac{\partial\mathcal{L}}{\partial\mathbf{W}_1} + \frac{\partial\mathcal{L}}{\partial\mathbf{W}_2}\mathbf{W}_1 - \eta \frac{\partial\mathcal{L}}{\partial\mathbf{W}_2} \frac{\partial\mathcal{L}}{\partial\mathbf{W}_1} \right) \mathbf{X} \end{align*}

\begin{align*} \left[\eta \to 0\right]{} \frac{\textrm{d}}{\textrm dt} F\left(\mathbf{X}\right) &amp; = - \left(\mathbf{W}_2 \frac{\partial\mathcal{L}}{\partial\mathbf{W}_1} + \frac{\partial\mathcal{L}}{\partial\mathbf{W}_2}\mathbf{W}_1 \right)\mathbf{X}. \end{align*}

Substituting the partial derivatives

\begin{align*} \frac{\partial\mathcal{L}}{\partial\mathbf{W}_1} &amp; = \frac{1}{2} \frac{\partial}{\partial\mathbf{W}_1} ||\mathbf{W}_2\mathbf{W}_1\mathbf{X} - \mathbf{Y}||^2_F \end{align*}

\begin{align*} &amp; = \mathbf{W}_2^{\textrm T}\left(\mathbf{W}_2\mathbf{W}_1\mathbf{X} - \mathbf{Y}\right)\mathbf{X}^{\textrm T} \end{align*}

and

\begin{align*} \frac{\partial\mathcal{L}}{\partial\mathbf{W}_2} &amp; = \frac{1}{2} \frac{\partial}{\partial\mathbf{W}_2} ||\mathbf{W}_2\mathbf{W}_1\mathbf{X} - \mathbf{Y}||^2_F \end{align*}

\begin{align*} &amp; = \left(\mathbf{W}_2\mathbf{W}_1\mathbf{X} - \mathbf{Y}\right)\mathbf{X}^{\textrm T}\mathbf{W}_1^{\textrm T} \end{align*}

then yields

\begin{equation*} \frac{\textrm{d}}{\textrm dt} F\left(X\right) = -\mathbf{W}_2\mathbf{W}_2^{\textrm T}\left(\mathbf{W}_2\mathbf{W}_1\mathbf{X} - \mathbf{Y}\right)\mathbf{X}^{\textrm T}\mathbf{X} - \left(\mathbf{W}_2\mathbf{W}_1\mathbf{X} - \mathbf{Y}\right)\mathbf{X}^{\textrm T}\mathbf{W}_1^{\textrm T}\mathbf{W}_1\mathbf{X}\text{.} \end{equation*}

Finally, we introduce the identity matrix $\mathbf{I}_{N_\textrm o}$ of size $N_\textrm o$ and apply row-wise vectoriasation $\mathrm{vec}_{\mathrm{r}}(F(\mathbf{X})) : = f(\mathbf{X})$ and the identity $\mathrm{vec}_{\mathrm{r}}(ABC) = (A \otimes C^{\textrm T})\mathrm{vec}_{\mathrm{r}}(B)$ to derive the neural tangent kernel

\begin{align*} \frac{\textrm{d}}{\textrm dt} F\left(X\right) &amp; = -\mathbf{W}_2\mathbf{W}_2^{\textrm T}\left(\mathbf{W}_2\mathbf{W}_1\mathbf{X} - \mathbf{Y}\right)\mathbf{X}^{\textrm T}\mathbf{X} - \mathbf{I}_{N_\textrm o}\left(\mathbf{W}_2\mathbf{W}_1\mathbf{X} - \mathbf{Y}\right)\mathbf{X}^{\textrm T}\mathbf{W}_1^{\textrm T}\mathbf{W}_1\mathbf{X} \end{align*}

\begin{align*} \Leftrightarrow\frac{\textrm{d}}{\textrm dt} f\left(\mathbf{X}\right) &amp; = - \left(\underbrace{\mathbf{W}_2\mathbf{W}_2^{\textrm T} \otimes \mathbf{X}^{\textrm T}\mathbf{X} + \mathbf{I} \otimes \mathbf{X}^{\textrm T}\mathbf{W}_1^{\textrm T}\mathbf{W}_1\mathbf{X}}_{\mathrm{NTK}}\right) \mathrm{vec}_{\mathrm{r}}\left(\mathbf{W}_2\mathbf{W}_1\mathbf{X} - \mathbf{Y}\right) \end{align*}

\begin{align*} &amp; = - \left(\left[\mathbf{W}_2 \otimes \mathbf{X}^{\textrm T}, \mathbf{I} \otimes \mathbf{X}^{\textrm T}\mathbf{W}_1^{\textrm T}\right]\left[\mathbf{W}_2 \otimes \mathbf{X}^{\textrm T}, \mathbf{I} \otimes \mathbf{X}^{\textrm T}\mathbf{W}_1^{\textrm T}\right]^{\textrm T}\right) \mathrm{vec}_{\mathrm{r}}\left(\frac{\partial\mathcal{L}}{\partial F}\right) \end{align*}

\begin{align*} &amp; = - \left(\left[\nabla_{\mathbf{W}_1} f, \nabla_{\mathbf{W}_2} f\right]\left[\nabla_{\mathbf{W}_1} f, \nabla_{\mathbf{W}_2} f\right]^{\textrm T}\right) \frac{\partial\mathcal{L}}{\partial f} \end{align*}

\begin{align*} &amp; = - \left(\nabla_\theta f \nabla_\theta f^{\textrm T}\right) \frac{\partial\mathcal{L}}{\partial f}, \end{align*}

where $[A, B]$ denotes concatenation.

## Appendix C Exact learning dynamics with prior knowledge

### C.1. Proof of theorem 3.1

In the following, we prove that equation (11) is in fact a solution to the matrix Riccati equation arising from gradient flow (equation (39)). We prove the theorem by directly substituting our solution for $\mathbf{Q}\mathbf{Q}^{\textrm T}(t)$ into the matrix Riccati equation.

#### C.1.1. Unequal input-output dimension.

We start with the following equation

\begin{align*} \mathbf{Q}\mathbf{Q}^{\textrm T}\left(t\right) = &amp;\underbrace{\left[\mathbf{O}e^{\mathbf{\Lambda}\frac{t}{\tau}}\mathbf{O}^{\textrm T} + 2\mathbf{M}\mathbf{M}^{\textrm T}\right] \mathbf{Q}\left(0\right)}_{\mathbf{L}} \nonumber \\ &amp; \times\underbrace{\left[\mathbf{I} + \frac{1}{2}\mathbf{Q}\left(0\right)^{\textrm T} \left(\mathbf{O} \left(e^{2\mathbf{\Lambda}\frac{t}{\tau}} - \mathbf{I}\right) \mathbf{\Lambda}^{-1} \mathbf{O}^{\textrm T} + 4\frac{t}{\tau} \mathbf{M}\mathbf{M}^{\textrm T}\right) \mathbf{Q}\left(0\right)\right]^{-1}}_{\mathbf{C}^{-1}} \end{align*}

\begin{align*} &amp; \times\underbrace{\mathbf{Q}\left(0\right)^{\textrm T} \left[\mathbf{O}e^{\mathbf{\Lambda}\frac{t}{\tau}}\mathbf{O}^{\textrm T} + 2\mathbf{M}\mathbf{M}^{\textrm T}\right]}_{\mathbf{R}} \nonumber\\ = &amp;\, \mathbf{L}\mathbf{C}^{-1}\mathbf{R}, \end{align*}

which is identical to equation (11) in the main text, as we verify in section C.2 (by reversing the derivation from equation (152) to equation (128)). Substituting our solution into the matrix Riccati equation then yields

\begin{align*} \tau\frac{\textrm{d}}{\textrm dt}\mathbf{Q}\mathbf{Q}^{\textrm T} &amp; = \mathbf{F}\mathbf{Q}\mathbf{Q}^{\textrm T}+\mathbf{Q}\mathbf{Q}^{\textrm T}\mathbf{F} -\left(\mathbf{Q}\mathbf{Q}^{\textrm T}\right)^2 \end{align*}

\begin{align*} \Rightarrow \tau\frac{\textrm{d}}{\textrm dt} \mathbf{L}\mathbf{C}^{-1}\mathbf{R} &amp;\stackrel{?}{ = } \mathbf{F}\mathbf{L}\mathbf{C}^{-1}\mathbf{R} + \mathbf{L}\mathbf{C}^{-1}\mathbf{R}\mathbf{F} - \mathbf{L}\mathbf{C}^{-1}\mathbf{R}\mathbf{L}\mathbf{C}^{-1}\mathbf{R}\text{.} \end{align*}

Next, we note that

\begin{equation*} \mathbf{O}^{\textrm T}\mathbf{O} = \frac{1}{\sqrt{2}} \begin{bmatrix} {\tilde{\mathbf{V}}} &amp; {\tilde{\mathbf{V}}}\\ {\tilde{\mathbf{U}}} &amp; -{\tilde{\mathbf{U}}} \end{bmatrix}^{\textrm T} \frac{1}{\sqrt{2}} \begin{bmatrix} {\tilde{\mathbf{V}}} &amp; {\tilde{\mathbf{V}}}\\ {\tilde{\mathbf{U}}} &amp; -{\tilde{\mathbf{U}}} \end{bmatrix} = \mathbf{I}, \end{equation*}

\begin{align*} \mathbf{O}^{\textrm T}\mathbf{M} &amp; = \frac{1}{\sqrt{2}} \begin{bmatrix} {\tilde{\mathbf{V}}}^{\textrm T} &amp; {\tilde{\mathbf{U}}}^{\textrm T}\\ {\tilde{\mathbf{V}}}^{\textrm T} &amp; -{\tilde{\mathbf{U}}}^{\textrm T} \end{bmatrix} \frac{1}{\sqrt{2}} \begin{bmatrix} {\tilde{\mathbf{V}}_\perp}\\ {\tilde{\mathbf{U}}_\perp} \end{bmatrix} \end{align*}

\begin{align*} &amp; = \frac{1}{2} \begin{bmatrix} {\tilde{\mathbf{V}}}^{\textrm T}{\tilde{\mathbf{V}}_\perp} + {\tilde{\mathbf{U}}}^{\textrm T}{\tilde{\mathbf{U}}_\perp}\\ {\tilde{\mathbf{V}}}^{\textrm T}{\tilde{\mathbf{V}}_\perp} -{\tilde{\mathbf{U}}}^{\textrm T}{\tilde{\mathbf{U}}_\perp} \end{bmatrix} \end{align*}

\begin{align*} &amp; = \mathbf{0} \end{align*}

and

\begin{align*} \mathbf{M}^{\textrm T}\mathbf{O} &amp; = \frac{1}{\sqrt{2}} \begin{bmatrix} {\tilde{\mathbf{V}}_\perp}^{\textrm T} &amp; {\tilde{\mathbf{U}}_\perp}^{\textrm T} \end{bmatrix} \frac{1}{\sqrt{2}} \begin{bmatrix} {\tilde{\mathbf{V}}} &amp; {\tilde{\mathbf{V}}}\\ {\tilde{\mathbf{U}}} &amp; -{\tilde{\mathbf{U}}} \end{bmatrix} \end{align*}

\begin{align*} &amp; = \frac{1}{2} \begin{bmatrix} {\tilde{\mathbf{V}}_\perp}^{\textrm T}{\tilde{\mathbf{V}}} + {\tilde{\mathbf{U}}_\perp}^{\textrm T}{\tilde{\mathbf{U}}}\\ {\tilde{\mathbf{V}}_\perp}^{\textrm T}{\tilde{\mathbf{V}}} -{\tilde{\mathbf{U}}_\perp}^{\textrm T}{\tilde{\mathbf{U}}} \end{bmatrix} \end{align*}

\begin{align*} &amp; = \mathbf{0}. \end{align*}

Then, using the chain rule $\partial(\mathbf{A}\mathbf{B}) = (\partial\mathbf{A})\mathbf{B} + \mathbf{A}(\partial\mathbf{B})$ and the identities

\begin{equation*} \frac{\textrm{d}}{d t}\left(\mathbf{A}^{-1}\right) = \mathbf{A}^{-1}\left(\frac{\textrm{d}}{d t}\mathbf{A}\right)\mathbf{A}^{-1} \qquad\text{and}\qquad\frac{\textrm{d}}{\textrm dt}\left(e^{t\mathbf{A}}\right) = \mathbf{A} e^{t\mathbf{A}} = e^{t\mathbf{A}}\mathbf{A} \end{equation*}

we get

\begin{align*} \tau\frac{\textrm{d}}{\textrm dt}\mathbf{Q}\mathbf{Q}^{\textrm T} &amp; = \tau\frac{\textrm{d}}{\textrm dt} \left(\mathbf{L}\mathbf{C}^{-1}\mathbf{R}\right) \end{align*}

\begin{align*} &amp; = \tau\left(\frac{\textrm{d}}{\textrm dt}\mathbf{L}\right)\mathbf{C}^{-1}\mathbf{R} + \tau\mathbf{L}\left(\frac{\textrm{d}}{\textrm dt} C^{-1}\mathbf{R}\right) \end{align*}

\begin{align*} &amp; = \tau\left(\frac{\textrm{d}}{\textrm dt}\mathbf{L}\right)\mathbf{C}^{-1}\mathbf{R} + \tau\mathbf{L}\mathbf{C}^{-1}\left(\frac{\textrm{d}}{\textrm dt} \mathbf{R}\right) + \tau\mathbf{L} \left(\frac{\textrm{d}}{\textrm dt} \mathbf{C}^{-1}\right)\mathbf{R} \text{,} \end{align*}

with

\begin{align*} \tau\left(\frac{\textrm{d}}{\textrm dt}\mathbf{L}\right)\mathbf{C}^{-1}\mathbf{R} &amp; = \tau\mathbf{O}\frac{1}{\tau}\boldsymbol{\Lambda}e^{{\boldsymbol{\Lambda}\frac{t}{\tau}}}\mathbf{O}^{\textrm T}\mathbf{Q}\left(0\right) \mathbf{C}^{-1}\mathbf{R} \end{align*}

\begin{align*} \quad &amp; = \mathbf{O}\boldsymbol{\Lambda}e^{{\boldsymbol{\Lambda}\frac{t}{\tau}}}\mathbf{O}^{\textrm T}\mathbf{Q}\left(0\right) \mathbf{C}^{-1}\mathbf{R} \end{align*}

\begin{align*} \quad &amp; = \left[\mathbf{O}\boldsymbol{\Lambda}\mathbf{O}^{\textrm T} \mathbf{O}e^{{\boldsymbol{\Lambda}\frac{t}{\tau}}}\mathbf{O}^{\textrm T}\mathbf{Q}\left(0\right) + 2\mathbf{O}\boldsymbol{\Lambda} \underbrace{\mathbf{O}^{\textrm T}\mathbf{M}}_\mathbf{0}\mathbf{M}^{\textrm T}\mathbf{Q}\left(0\right)\right] \mathbf{C}^{-1}\mathbf{R} \end{align*}

\begin{align*} \quad &amp; = \mathbf{F}\mathbf{L}\mathbf{C}^{-1}\mathbf{R}, \end{align*}

\begin{align*} \tau\mathbf{L}\mathbf{C}^{-1}\left(\frac{\textrm{d}}{\textrm dt} \mathbf{R}\right) &amp; = \tau\mathbf{L}\mathbf{C}^{-1}\mathbf{Q}\left(0\right)^{\textrm T}\mathbf{O}\frac{1}{\tau}e^{{\boldsymbol{\Lambda}\frac{t}{\tau}}}\boldsymbol{\Lambda}\mathbf{O}^{\textrm T} \end{align*}

\begin{align*} \quad &amp; = \mathbf{L}\mathbf{C}^{-1}\mathbf{Q}\left(0\right)^{\textrm T}\mathbf{O}e^{{\boldsymbol{\Lambda}\frac{t}{\tau}}}\boldsymbol{\Lambda}\mathbf{O}^{\textrm T} \end{align*}

\begin{align*} \quad &amp; = \mathbf{L}\mathbf{C}^{-1}\left[\mathbf{Q}\left(0\right)^{\textrm T}\mathbf{O}e^{{\boldsymbol{\Lambda}\frac{t}{\tau}}}\mathbf{O}^{\textrm T}\mathbf{O}\boldsymbol{\Lambda}\mathbf{O}^{\textrm T} + 2\mathbf{Q}\left(0\right)^{\textrm T}\mathbf{M} \underbrace{\mathbf{M}^{\textrm T}\mathbf{O}}_\mathbf{0 }\boldsymbol{\Lambda}\mathbf{O}^{\textrm T}\right] \end{align*}

\begin{align*} \quad &amp; = \mathbf{L}\mathbf{C}^{-1}\mathbf{R}\mathbf{F} \end{align*}

and

\begin{align*} \tau\mathbf{L} \left(\frac{\textrm{d}}{\textrm dt} \mathbf{C}^{-1}\right)\mathbf{R} &amp; = -\tau\mathbf{L} \mathbf{C}^{-1}\left(\frac{\textrm{d}}{\textrm dt} \mathbf{C}\right)\mathbf{C}^{-1}\mathbf{R} \end{align*}

\begin{align*} &amp; = -\mathbf{L} \mathbf{C}^{-1}\bigg[\tau\frac{1}{2}\mathbf{Q}\left(0\right)^{\textrm T}\mathbf{O} 2\frac{1}{\tau}e^{2\boldsymbol{\Lambda}\frac{t}{\tau}}\boldsymbol{\Lambda}\boldsymbol{\Lambda}^{-1}\mathbf{O}^{\textrm T}\mathbf{Q}\left(0\right) \end{align*}

\begin{align*} &amp;\quad + \tau\frac{1}{2}\mathbf{Q}\left(0\right)^{\textrm T} 4\frac{1}{\tau}\mathbf{M}\mathbf{M}^{\textrm T}\mathbf{Q}\left(0\right)\bigg]\mathbf{C}^{-1}\mathbf{R} \nonumber\\ &amp; = -\mathbf{L}\mathbf{C}^{-1} \bigg[\mathbf{Q}\left(0\right)^{\textrm T}\mathbf{O}e^{2\boldsymbol{\Lambda}\frac{t}{\tau}}\mathbf{O}^{\textrm T}\mathbf{Q}\left(0\right) + 2\mathbf{Q}\left(0\right)^{\textrm T}\mathbf{M}\mathbf{M}^{\textrm T}\mathbf{Q}\left(0\right)\bigg] \mathbf{C}^{-1}\mathbf{R} \end{align*}

\begin{align*} &amp; = -\mathbf{L}\mathbf{C}^{-1} \bigg[\mathbf{Q}\left(0\right)^{\textrm T}\mathbf{O}e^{{\boldsymbol{\Lambda}\frac{t}{\tau}}}\mathbf{O}^{\textrm T}\mathbf{O}e^{{\boldsymbol{\Lambda}\frac{t}{\tau}}}\mathbf{O}^{\textrm T}\mathbf{Q}\left(0\right) \nonumber\\ &amp;\quad + 2\mathbf{Q}\left(0\right)^{\textrm T}\mathbf{O}e^{{\boldsymbol{\Lambda}\frac{t}{\tau}}}\underbrace{\mathbf{O}^{\textrm T}\mathbf{M}}_\mathbf{0}\mathbf{M}^{\textrm T}\mathbf{Q}\left(0\right) \end{align*}

\begin{align*} &amp;\quad + 2\mathbf{Q}\left(0\right)^{\textrm T}\mathbf{M}\underbrace{\mathbf{M}^{\textrm T}\mathbf{O}}_\mathbf{0}e^{{\boldsymbol{\Lambda}\frac{t}{\tau}}}\mathbf{O}^{\textrm T}\mathbf{Q}\left(0\right) \nonumber\\ &amp;\quad + 4\mathbf{Q}\left(0\right)^{\textrm T}\mathbf{M}\mathbf{M}^{\textrm T}\mathbf{M}\mathbf{M}^{\textrm T}\mathbf{Q}\left(0\right)\bigg] \mathbf{C}^{-1}\mathbf{R} \nonumber\\ &amp; = -\mathbf{L}\mathbf{C}^{-1} \mathbf{R}\mathbf{L} \mathbf{C}^{-1}\mathbf{R}. \end{align*}

Finally, substituting equations (82), (86) and (90) into the left hand side of equation (70) proves equality.

$\square$

#### C.1.2. Equal input-output dimension.

In the case of equal input-output dimensions ${\tilde{\mathbf{U}}_\perp} = {\tilde{\mathbf{V}}_\perp} = 0$ equation (67) reduces to

\begin{align*} \mathbf{Q}\mathbf{Q}^{\textrm T}\left(t\right) &amp; = \underbrace{\mathbf{O}e^{{\boldsymbol{\Lambda}\frac{t}{\tau}}}\mathbf{O}^{\textrm T}\mathbf{Q}\left(0\right)}_{\mathbf{L}} \nonumber\\ &amp; \quad\ \,\,\times \underbrace{\left[\mathbf{I} + \frac{1}{2}\mathbf{Q}\left(0\right)^{\textrm T}\mathbf{O}e^{2\boldsymbol{\Lambda}\frac{t}{\tau}}\boldsymbol{\Lambda}^{-1} \mathbf{O}^{\textrm T}\mathbf{Q}\left(0\right) - \frac{1}{2}\mathbf{Q}\left(0\right)^{\textrm T}\mathbf{O}\boldsymbol{\Lambda}^{-1}\mathbf{O}^{\textrm T}\mathbf{Q}\left(0\right)\right]^{-1}}_{\mathbf{C}^{-1}} \end{align*}

\begin{align*} &amp; \times\underbrace{\mathbf{Q}\left(0\right)^{\textrm T} \mathbf{O}e^{{\boldsymbol{\Lambda}\frac{t}{\tau}}}\mathbf{O}^{\textrm T}}_{\mathbf{R}} = \mathbf{L}\mathbf{C}^{-1}\mathbf{R}\text{.} \end{align*}

Therefore, analogously to the proof for unequal input-output dimensions, it follows that

\begin{align*} \tau\frac{\textrm{d}}{\textrm dt}\mathbf{Q}\mathbf{Q}^{\textrm T} &amp; = \tau\frac{\textrm{d}}{\textrm dt} \mathbf{L}\mathbf{C}^{-1}\mathbf{R} \end{align*}

\begin{align*} &amp; = \tau\left(\frac{\textrm{d}}{\textrm dt}\mathbf{L}\right)\mathbf{C}^{-1}\mathbf{R} + \tau\mathbf{L}\left(\frac{\textrm{d}}{\textrm dt} C^{-1}\mathbf{R}\right) \end{align*}

\begin{align*} &amp; = \tau\left(\frac{\textrm{d}}{\textrm dt}\mathbf{L}\right)\mathbf{C}^{-1}\mathbf{R} + \tau\mathbf{L}\mathbf{C}^{-1}\left(\frac{\textrm{d}}{\textrm dt} \mathbf{R}\right) + \tau\mathbf{L} \left(\frac{\textrm{d}}{\textrm dt} \mathbf{C}^{-1}\right)\mathbf{R} \text{,} \end{align*}

with

\begin{align*} \tau\left(\frac{\textrm{d}}{\textrm dt}\mathbf{L}\right)\mathbf{C}^{-1}\mathbf{R} &amp; = \tau\mathbf{O}\boldsymbol{\Lambda}\frac{1}{\tau}e^{{\boldsymbol{\Lambda}\frac{t}{\tau}}}\mathbf{O}^{\textrm T}\mathbf{Q}\left(0\right)\mathbf{C}^{-1}\mathbf{R} \end{align*}

\begin{align*} &amp; = \mathbf{O}\boldsymbol{\Lambda}\mathbf{O}^{\textrm T}\mathbf{O}e^{{\boldsymbol{\Lambda}\frac{t}{\tau}}}\mathbf{O}^{\textrm T}\mathbf{Q}\left(0\right)\mathbf{C}^{-1}\mathbf{R} \end{align*}

\begin{align*} &amp; = \mathbf{F}\mathbf{L}\mathbf{C}^{-1}\mathbf{R}\text{,} \end{align*}

\begin{align*} \tau\mathbf{L}\mathbf{C}^{-1}\left(\frac{\textrm{d}}{\textrm dt}\mathbf{R}\right) &amp; = \tau\mathbf{L}\mathbf{C}^{-1}\mathbf{Q}\left(0\right)^{\textrm T}\mathbf{O}\frac{1}{\tau}e^{{\boldsymbol{\Lambda}\frac{t}{\tau}}}\boldsymbol{\Lambda}\mathbf{O}^{\textrm T} \end{align*}

\begin{align*} &amp; = \mathbf{L}\mathbf{C}^{-1}\mathbf{Q}\left(0\right)^{\textrm T}\mathbf{O}e^{{\boldsymbol{\Lambda}\frac{t}{\tau}}}\mathbf{O}^{\textrm T}\mathbf{O}\boldsymbol{\Lambda}\mathbf{O}^{\textrm T} \end{align*}

\begin{align*} &amp; = \mathbf{L}\mathbf{C}^{-1}\mathbf{R}\mathbf{F}\text{,} \end{align*}

and

\begin{align*} \tau\mathbf{L}\left(\frac{\textrm{d}}{\textrm dt}\mathbf{C}^{-1}\mathbf{R}\right) &amp; = -\tau\mathbf{L}\mathbf{C}^{-1}\left(\frac{\textrm{d}}{\textrm dt}\mathbf{C}\right)\mathbf{C}^{-1}\mathbf{R} \end{align*}

\begin{align*} &amp; = -\tau\mathbf{L}\mathbf{C}^{-1}\left(\frac{1}{2}\mathbf{Q}\left(0\right)^{\textrm T}\mathbf{O}e^{2\boldsymbol{\Lambda}\frac{t}{\tau}}\frac{2}{\tau}\boldsymbol{\Lambda}\boldsymbol{\Lambda}^{-1}\mathbf{O}^{\textrm T}\mathbf{Q}\left(0\right)\right)\mathbf{C}^{-1}\mathbf{R} \end{align*}

\begin{align*} &amp; = -\tau\mathbf{L}\mathbf{C}^{-1}\mathbf{Q}\left(0\right)^{\textrm T}\mathbf{O}e^{{\boldsymbol{\Lambda}\frac{t}{\tau}}}\mathbf{O}^{\textrm T}\mathbf{O}e^{{\boldsymbol{\Lambda}\frac{t}{\tau}}}\mathbf{Q}\left(0\right)\mathbf{C}^{-1}\mathbf{R} \end{align*}

\begin{align*} &amp; = -\mathbf{L}\mathbf{C}^{-1}\mathbf{R}\mathbf{L}\mathbf{C}^{-1}\mathbf{R}. \end{align*}

Finally, substituting equations (100), (103) and (106) into the left hand side of equation (70) proves equality.

$\square$

### C.2. Derivation of the exact learning dynamics

In the following, we outline how the solution to the matrix Ricatti equation can be acquired. Let the input and output dimension of a two-layer linear network (equation (1)) be denoted by $N_\textrm i$ and $N_\textrm o$ respectively. Further, let $N_m = \min(N_\textrm i, N_\textrm o)$ denote the smaller one of the two. The compact singular value decomposition of the initial network function and the input-output correlation of the task is then

\begin{equation*} \mathrm{SVD}\left(\mathbf{W}_2\left(0\right)\mathbf{W}_1\left(0\right)\right) = \mathbf{U}\mathbf{S}\mathbf{V}^{\textrm T} \,\text{and} \,\mathrm{SVD}\left(\boldsymbol{\tilde{\Sigma}}^{yx}\right) = {\tilde{\mathbf{U}}}{\tilde{\mathbf{S}}}{\tilde{\mathbf{V}}}^{\textrm T}. \end{equation*}

Here, **U** and ${\tilde{\mathbf{U}}} \in \mathbb{R}^{N_\textrm o \times N_m}$ denote the left singular vectors, **S** and ${\tilde{\mathbf{S}}} \in \mathbb{R}^{N_m \times N_m}$ the square matrix with ordered, non-zero eigenvalues on its diagonal and **V** and $\mathbf{\tilde{V}} \in \mathbb{R}^{N_\textrm i \times N_m}$ the corresponding right singular vectors. Please note that when using compact singular value decomposition, in the case of unequal input-output dimensions ($N_\textrm i \neq N_\textrm o$) the right and left singular vectors are not generally square and orthonormal.

More specifically, in the case of $N_\textrm i < N_\textrm o$, ${\tilde{\mathbf{U}}}^{\textrm T}{\tilde{\mathbf{U}}} = \mathbf{\tilde{V}}^{\textrm T}\mathbf{\tilde{V}} = \mathbf{\tilde{V}}\mathbf{\tilde{V}}^{\textrm T} = \mathbf{I} \in \mathbb{R}^{N_\textrm i \times N_\textrm i}$ but ${\tilde{\mathbf{U}}}{\tilde{\mathbf{U}}}^{\textrm T} \neq \mathbf{I} \in \mathbb{R}^{N_\textrm o \times N_\textrm o}$. In this case, we use ${\tilde{\mathbf{U}}_\perp} \in \mathbb{R}^{N_\textrm o \times (N_\textrm o - N_\textrm i)}$ to denote the matrix that contains orthogonal column vectors such that the concatenation $\big[{\tilde{\mathbf{U}}} ~ {\tilde{\mathbf{U}}_\perp}\big]$ is orthonormal and ${\tilde{\mathbf{V}}_\perp} \in \mathbb{R}^{N_\textrm i \times (N_\textrm o - N_\textrm i)}$ to denote a matrix of zeros.

Conversely, in the case of $N_\textrm i > N_\textrm o$, ${\tilde{\mathbf{U}}}{\tilde{\mathbf{U}}}^{\textrm T} = {\tilde{\mathbf{U}}}^{\textrm T}{\tilde{\mathbf{U}}} = \mathbf{\tilde{V}}^{\textrm T}\mathbf{\tilde{V}} = \mathbf{I} \in \mathbb{R}^{N_\textrm o \times N_\textrm o}$ but $\mathbf{\tilde{V}}^{\textrm T}\mathbf{\tilde{V}} \neq \mathbf{I} \in \mathbb{R}^{N_\textrm i \times N_\textrm i}$ and we define ${\tilde{\mathbf{V}}_\perp} \in \mathbb{R}^{N_\textrm i \times (N_\textrm i - N_\textrm o)}$ such that $\big[\mathbf{\tilde{V}} ~ {\tilde{\mathbf{V}}_\perp}\big]$ is orthonormal and ${\tilde{\mathbf{U}}_\perp} \in \mathbb{R}^{N_\textrm o \times (N_\textrm o - N_\textrm i)}$ to denote a matrix of zeros.

#### C.2.1. Inverse and matrix exponential of **F**.

The solution to the matrix Riccati equation as provided by Fukumizu (1998) requires calculation of the inverse **F** ^−1^ and the matrix exponential $e^{\mathbf{F}\frac{t}{\tau}}$. To this end, we diagonalise **F** by completing its basis by incorporating zero eigenvalues as illustrated below

\begin{align*} \mathbf{F} &amp; = \begin{bmatrix} 0 &amp; {\tilde{\mathbf{V}}}{\tilde{\mathbf{S}}}{\tilde{\mathbf{U}}}^{\textrm T}\\ {\tilde{\mathbf{U}}}{\tilde{\mathbf{S}}}{\tilde{\mathbf{V}}}^{\textrm T} &amp; 0 \end{bmatrix} \end{align*}

\begin{align*} &amp; = \frac{1}{\sqrt{2}} \begin{bmatrix} {\tilde{\mathbf{V}}} &amp; {\tilde{\mathbf{V}}} &amp; \sqrt{2}{\tilde{\mathbf{V}}_\perp} \\ {\tilde{\mathbf{U}}} &amp; -{\tilde{\mathbf{U}}} &amp; \sqrt{2}{\tilde{\mathbf{U}}_\perp} \end{bmatrix} \begin{bmatrix} {\tilde{\mathbf{S}}} &amp; 0 &amp; 0 \\ 0 &amp; -{\tilde{\mathbf{S}}} &amp; 0\\ 0 &amp; 0 &amp; 0\\ \end{bmatrix} \frac{1}{\sqrt{2}} \begin{bmatrix} {\tilde{\mathbf{V}}} &amp; {\tilde{\mathbf{V}}} &amp; \sqrt{2}{\tilde{\mathbf{V}}_\perp} \\ {\tilde{\mathbf{U}}} &amp; -{\tilde{\mathbf{U}}} &amp; \sqrt{2}{\tilde{\mathbf{U}}_\perp} \end{bmatrix}^{\textrm T} \end{align*}

\begin{align*} &amp; = \mathbf{P}\boldsymbol{\Gamma}\mathbf{P}^{\textrm T}. \end{align*}

Note that $\mathbf{P}^{\textrm T}\mathbf{P} = \mathbf{P}\mathbf{P}^{\textrm T} = \mathbf{I}$ and therefore $\mathbf{P}^{\textrm T} = \mathbf{P}^{-1}$. We then use the diagonalisation of **F** to rewrite the matrix exponential

\begin{align*} e^{\mathbf{F}\frac{t}{\tau}} &amp; = \mathbf{P} e^{\boldsymbol{\Gamma}} \mathbf{P}^{\textrm T} \end{align*}

\begin{align*} &amp; = \frac{1}{\sqrt{2}} \begin{bmatrix} {\tilde{\mathbf{V}}} &amp; {\tilde{\mathbf{V}}} &amp; \sqrt{2}\mathbf{V_\perp} \\ {\tilde{\mathbf{U}}} &amp; -{\tilde{\mathbf{U}}} &amp; \sqrt{2}\mathbf{U_\perp} \end{bmatrix} \begin{bmatrix} e^{{\tilde{\mathbf{S}}}\frac{t}{\tau}} &amp; 0 &amp; 0\\ 0 &amp; e^{-{\tilde{\mathbf{S}}}\frac{t}{\tau}} &amp; 0\\ 0 &amp; 0 &amp; e^0\\ \end{bmatrix} \frac{1}{\sqrt{2}} \begin{bmatrix} {\tilde{\mathbf{V}}} &amp; {\tilde{\mathbf{V}}} &amp; \sqrt{2}\mathbf{V_\perp} \\ {\tilde{\mathbf{U}}} &amp; -{\tilde{\mathbf{U}}} &amp; \sqrt{2}\mathbf{U_\perp} \end{bmatrix}^{\textrm T} \end{align*}

\begin{align*} &amp; = \frac{1}{2} \begin{bmatrix} {\tilde{\mathbf{V}}} e^{{\tilde{\mathbf{S}}}\frac{t}{\tau}}{\tilde{\mathbf{V}}}^{\textrm T} + {\tilde{\mathbf{V}}} e^{-{\tilde{\mathbf{S}}}\frac{t}{\tau}}{\tilde{\mathbf{V}}}^{\textrm T} + 2{\tilde{\mathbf{V}}_\perp}{\tilde{\mathbf{V}}_\perp}^{\textrm T} &amp; {\tilde{\mathbf{V}}} e^{{\tilde{\mathbf{S}}}\frac{t}{\tau}}{\tilde{\mathbf{U}}}^{\textrm T} - {\tilde{\mathbf{V}}} e^{-{\tilde{\mathbf{S}}}\frac{t}{\tau}}{\tilde{\mathbf{U}}}^{\textrm T} + 2{\tilde{\mathbf{V}}_\perp}{\tilde{\mathbf{U}}_\perp}^{\textrm T} \\ {\tilde{\mathbf{U}}} e^{{\tilde{\mathbf{S}}}\frac{t}{\tau}}{\tilde{\mathbf{V}}}^{\textrm T} - {\tilde{\mathbf{U}}} e^{-{\tilde{\mathbf{S}}}\frac{t}{\tau}}{\tilde{\mathbf{V}}}^{\textrm T} + 2{\tilde{\mathbf{U}}_\perp}{\tilde{\mathbf{V}}_\perp}^{\textrm T} &amp; {\tilde{\mathbf{U}}} e^{{\tilde{\mathbf{S}}}\frac{t}{\tau}}{\tilde{\mathbf{U}}}^{\textrm T} - {\tilde{\mathbf{U}}} e^{-{\tilde{\mathbf{S}}}\frac{t}{\tau}}{\tilde{\mathbf{U}}}^{\textrm T} + 2{\tilde{\mathbf{U}}_\perp}{\tilde{\mathbf{U}}_\perp}^{\textrm T} \end{bmatrix} \end{align*}

\begin{align*} &amp; = \frac{1}{\sqrt{2}} \begin{bmatrix} {\tilde{\mathbf{V}}} &amp; {\tilde{\mathbf{V}}} \\ {\tilde{\mathbf{U}}} &amp; -{\tilde{\mathbf{U}}} \end{bmatrix} \begin{bmatrix} e^{{\tilde{\mathbf{S}}}\frac{t}{\tau}} &amp; 0\\ 0 &amp; e^{-{\tilde{\mathbf{S}}}\frac{t}{\tau}} \end{bmatrix}\frac{1}{\sqrt{2}} \begin{bmatrix} {\tilde{\mathbf{V}}} &amp; {\tilde{\mathbf{V}}} \\ {\tilde{\mathbf{U}}} &amp; -{\tilde{\mathbf{U}}} \end{bmatrix}^{\textrm T} + 2\frac{1}{\sqrt{2}} \begin{bmatrix} {\tilde{\mathbf{V}}_\perp}\\ {\tilde{\mathbf{U}}_\perp} \end{bmatrix} \frac{1}{\sqrt{2}} \begin{bmatrix} {\tilde{\mathbf{V}}_\perp}\\ {\tilde{\mathbf{U}}_\perp} \end{bmatrix}^{\textrm T} \end{align*}

\begin{align*} &amp; = \mathbf{O} e^{{\boldsymbol{\Lambda}\frac{t}{\tau}}} \mathbf{O} + 2 \mathbf{M} \mathbf{M}^{\textrm T}\text{.} \end{align*}

As the inverse $\mathbf{F}^{-1} = \mathbf{P}\boldsymbol{\Gamma}^{-1}\mathbf{P}^{\textrm T}$ is not well defined for a **Γ** with zero eigenvalues. We study eigenvalues of value zero by analysing the limiting behaviour of

\begin{equation*} e^{\mathbf{F}\frac{t}{\tau}}\mathbf{F}^{-1}e^{\mathbf{F}\frac{t}{\tau}} - \mathbf{F}^{-1} \end{equation*}

for a single mode

\begin{align*} \lim_{\epsilon \to 0} \left[e^{\frac{\epsilon t}{\tau}} \frac{1}{\epsilon} e^{\frac{\epsilon t}{\tau}} - \frac{1}{\epsilon}\right] = &amp;\lim_{\epsilon \to 0} \left[\frac{e^{\frac{2\epsilon t}{\tau}} - 1}{\epsilon}\right] \end{align*}

\begin{align*} \xrightarrow{{{{\text{L}}^\prime }{\text{Hospital}}}}\mathop {\lim }\limits_{ \to 0} \left[ {\frac{{\frac{\partial }{{\partial }}\left( {{e^{\frac{{2t}}{\tau }}} - 1} \right)}}{{\frac{\partial }{{\partial }}}}} \right] \end{align*}

\begin{align*} = &amp;\lim_{\epsilon \to 0} 2\frac{t}{\tau} e^{\frac{2\epsilon t}{\tau}} \end{align*}

\begin{align*} = &amp;\ 2 \frac{t}{\tau}\text{.} \end{align*}

which reveals the time dependent contribution of zero eigenvalues. Thus

\begin{align*} e^{\mathbf{F}\frac{t}{\tau}}\mathbf{F}^{-1}e^{\mathbf{F}\frac{t}{\tau}} - \mathbf{F}^{-1} &amp; = \mathbf{O}e^{{\boldsymbol{\Lambda}\frac{t}{\tau}}}\mathbf{O}^{\textrm T}\mathbf{O}\boldsymbol{\Lambda}^{-1}\mathbf{O}^{\textrm T}\mathbf{O}e^{{\boldsymbol{\Lambda}\frac{t}{\tau}}}\mathbf{O}^{\textrm T} - \mathbf{O}\boldsymbol{\Lambda}^{-1}\mathbf{O}^{\textrm T} + 4\frac{t}{\tau}\mathbf{M}\mathbf{M}^{\textrm T}. \end{align*}

We continue by substituting the above results into Fukumizu’s equation

\begin{align*} \mathbf{Q}\mathbf{Q}^{\textrm T}\left(t\right) &amp; = \left[\mathbf{O}e^{{\boldsymbol{\Lambda}\frac{t}{\tau}}}\mathbf{O}^{\textrm T} + 2\mathbf{M}\mathbf{M}^{\textrm T}\right] \mathbf{Q}\left(0\right) \end{align*}

\begin{align*} &amp; \quad \times \left[\mathbf{I} + \frac{1}{2}\mathbf{Q}\left(0\right)^{\textrm T}\left(\mathbf{O}e^{{\boldsymbol{\Lambda}\frac{t}{\tau}}}\mathbf{O}^{\textrm T} \mathbf{O} \boldsymbol{\Lambda}^{-1} \mathbf{O}^{\textrm T} \mathbf{O}e^{{\boldsymbol{\Lambda}\frac{t}{\tau}}}\mathbf{O}^{\textrm T} - \mathbf{O} \boldsymbol{\Lambda}^{-1} \mathbf{O}^{\textrm T} + 4\frac{t}{\tau} \mathbf{M}\mathbf{M}^{\textrm T} \right) \mathbf{Q}\left(0\right)\right]^{-1} \nonumber \\ &amp; \quad \times \mathbf{Q}\left(0\right)^{\textrm T} \left[\mathbf{O}e^{{\boldsymbol{\Lambda}\frac{t}{\tau}}}\mathbf{O}^{\textrm T} + 2\mathbf{M}\mathbf{M}^{\textrm T}\right] \nonumber \\ &amp; = \left[\mathbf{O}e^{{\boldsymbol{\Lambda}\frac{t}{\tau}}}\mathbf{O}^{\textrm T} + 2\mathbf{M}\mathbf{M}^{\textrm T}\right] \mathbf{Q}\left(0\right) \nonumber \\ &amp; \quad \times \left[\mathbf{I} + \frac{1}{2}\mathbf{Q}\left(0\right)^{\textrm T}\left(\mathbf{O}e^{{\boldsymbol{\Lambda}\frac{t}{\tau}}} \boldsymbol{\Lambda}^{-1} e^{{\boldsymbol{\Lambda}\frac{t}{\tau}}}\mathbf{O}^{\textrm T} - \mathbf{O} \boldsymbol{\Lambda}^{-1} \mathbf{O}^{\textrm T} + 4\frac{t}{\tau} \mathbf{M}\mathbf{M}^{\textrm T} \right) \mathbf{Q}\left(0\right)\right]^{-1} \end{align*}

\begin{align*} &amp; \mathbf{Q}\left(0\right)^{\textrm T} \left[\mathbf{O}e^{{\boldsymbol{\Lambda}\frac{t}{\tau}}}\mathbf{O}^{\textrm T} + 2\mathbf{M}\mathbf{M}^{\textrm T}\right] \nonumber \\ &amp; = \left[\mathbf{O}e^{{\boldsymbol{\Lambda}\frac{t}{\tau}}}\mathbf{O}^{\textrm T} + 2\mathbf{M}\mathbf{M}^{\textrm T}\right] \mathbf{Q}\left(0\right) \nonumber \\ &amp; \quad \times \left[\mathbf{I} + \frac{1}{2}\mathbf{Q}\left(0\right)^{\textrm T} \left(\mathbf{O} \left(e^{2\boldsymbol{\Lambda}\frac{t}{\tau}} \boldsymbol{\Lambda}^{-1} - \boldsymbol{\Lambda}^{-1}\right) \mathbf{O}^{\textrm T} + 4\frac{t}{\tau} \mathbf{M}\mathbf{M}^{\textrm T}\right) \mathbf{Q}\left(0\right) \right]^{-1} \end{align*}

\begin{align*} &amp; \quad \times \mathbf{Q}\left(0\right)^{\textrm T} \left[\mathbf{O}e^{{\boldsymbol{\Lambda}\frac{t}{\tau}}}\mathbf{O}^{\textrm T} + 2\mathbf{M}\mathbf{M}^{\textrm T}\right] \nonumber \\ &amp; = \left[\mathbf{O}e^{{\boldsymbol{\Lambda}\frac{t}{\tau}}}\mathbf{O}^{\textrm T} + 2\mathbf{M}\mathbf{M}^{\textrm T}\right] \mathbf{Q}\left(0\right) \nonumber \\ &amp; \quad \times \left[\mathbf{I} + \frac{1}{2}\mathbf{Q}\left(0\right)^{\textrm T} \left(\mathbf{O} \left(e^{2\boldsymbol{\Lambda}\frac{t}{\tau}} - \mathbf{I}\right) \boldsymbol{\Lambda}^{-1} \mathbf{O}^{\textrm T} + 4\frac{t}{\tau} \mathbf{M}\mathbf{M}^{\textrm T}\right) \mathbf{Q}\left(0\right)\right]^{-1} \end{align*}

\begin{align*} &amp;\quad\times \mathbf{Q}\left(0\right)^{\textrm T} \left[\mathbf{O}e^{{\boldsymbol{\Lambda}\frac{t}{\tau}}}\mathbf{O}^{\textrm T} + 2\mathbf{M}\mathbf{M}^{\textrm T}\right] \nonumber. \end{align*}

Then, matrix multiplication on the left side of the equation yields

\begin{align*} \mathbf{O} e^{{\boldsymbol{\Lambda}\frac{t}{\tau}}} &amp; = \frac{1}{\sqrt{2}} \begin{bmatrix} {\tilde{\mathbf{V}}} &amp; {\tilde{\mathbf{V}}}\\ {\tilde{\mathbf{U}}} &amp; -{\tilde{\mathbf{U}}} \end{bmatrix} \begin{bmatrix} e^{{\tilde{\mathbf{S}}}\frac{t}{\tau}} &amp; 0\\ 0 &amp; e^{-{\tilde{\mathbf{S}}}\frac{t}{\tau}} \end{bmatrix} \end{align*}

\begin{align*} &amp; = \frac{1}{\sqrt{2}} \begin{bmatrix} {\tilde{\mathbf{V}}} e^{{\tilde{\mathbf{S}}}\frac{t}{\tau}} &amp; {\tilde{\mathbf{V}}} e^{-{\tilde{\mathbf{S}}}\frac{t}{\tau}}\\ {\tilde{\mathbf{U}}} e^{{\tilde{\mathbf{S}}}\frac{t}{\tau}} &amp; -{\tilde{\mathbf{U}}} e^{-{\tilde{\mathbf{S}}}\frac{t}{\tau}} \end{bmatrix} \end{align*}

and

\begin{align*} \mathbf{O}^{\textrm T} \mathbf{Q}\left(0\right) &amp; = \frac{1}{\sqrt{2}} \begin{bmatrix} {\tilde{\mathbf{V}}} &amp; {\tilde{\mathbf{V}}}\\ {\tilde{\mathbf{U}}} &amp; -{\tilde{\mathbf{U}}} \end{bmatrix}^{\textrm T} \begin{bmatrix} \mathbf{V} \sqrt{\mathbf{S}} \mathbf{R}^{\textrm T}\\ \mathbf{U} \sqrt{\mathbf{S}} \mathbf{R}^{\textrm T} \end{bmatrix} \end{align*}

\begin{align*} &amp; = \frac{1}{\sqrt{2}} \begin{bmatrix} {\tilde{\mathbf{V}}}^{\textrm T} \mathbf{V} \sqrt{\mathbf{S}} \mathbf{R}^{\textrm T} + {\tilde{\mathbf{U}}}^{\textrm T} \mathbf{U} \sqrt{\mathbf{S}} \mathbf{R}^{\textrm T}\\ {\tilde{\mathbf{V}}}^{\textrm T} \mathbf{V} \sqrt{\mathbf{S}} \mathbf{R}^{\textrm T} - {\tilde{\mathbf{U}}}^{\textrm T} \mathbf{U} \sqrt{\mathbf{S}} \mathbf{R}^{\textrm T}\\ \end{bmatrix} \end{align*}

\begin{align*} &amp; = \frac{1}{\sqrt{2}} \begin{bmatrix} \left({\tilde{\mathbf{V}}}^{\textrm T} \mathbf{V} + {\tilde{\mathbf{U}}}^{\textrm T} \mathbf{U} \right) \sqrt{\mathbf{S}} \mathbf{R}^{\textrm T}\\ \left({\tilde{\mathbf{V}}}^{\textrm T} \mathbf{V} - {\tilde{\mathbf{U}}}^{\textrm T} \mathbf{U} \right) \sqrt{\mathbf{S}} \mathbf{R}^{\textrm T}\\ \end{bmatrix}\text{,} \end{align*}

such that

\begin{align*} \mathbf{O} e^{{\boldsymbol{\Lambda}\frac{t}{\tau}}} \mathbf{O}^{\textrm T} \mathbf{Q}\left(0\right) &amp; = \frac{1}{2} \begin{bmatrix} {\tilde{\mathbf{V}}} e^{{\tilde{\mathbf{S}}}\frac{t}{\tau}} &amp; {\tilde{\mathbf{V}}} e^{-{\tilde{\mathbf{S}}}\frac{t}{\tau}}\\ {\tilde{\mathbf{U}}} e^{{\tilde{\mathbf{S}}}\frac{t}{\tau}} &amp; -{\tilde{\mathbf{U}}} e^{-{\tilde{\mathbf{S}}}\frac{t}{\tau}} \end{bmatrix} \begin{bmatrix} {\tilde{\mathbf{V}}}^{\textrm T} \mathbf{V} \sqrt{\mathbf{S}} \mathbf{R}^{\textrm T} + {\tilde{\mathbf{U}}}^{\textrm T} \mathbf{U} \sqrt{\mathbf{S}} \mathbf{R}^{\textrm T}\\ {\tilde{\mathbf{V}}}^{\textrm T} \mathbf{V} \sqrt{\mathbf{S}} \mathbf{R}^{\textrm T} - {\tilde{\mathbf{U}}}^{\textrm T} \mathbf{U} \sqrt{\mathbf{S}} \mathbf{R}^{\textrm T}\\ \end{bmatrix} \end{align*}

\begin{align*} &amp; = \frac{1}{2} \begin{bmatrix} {\tilde{\mathbf{V}}} \left(e^{{\tilde{\mathbf{S}}}\frac{t}{\tau}} \left({\tilde{\mathbf{V}}}^{\textrm T}\mathbf{V} + {\tilde{\mathbf{U}}}^{\textrm T}\mathbf{U} \right) + e^{-{\tilde{\mathbf{S}}}\frac{t}{\tau}} \left({\tilde{\mathbf{V}}}^{\textrm T}\mathbf{V} - {\tilde{\mathbf{U}}}^{\textrm T}\mathbf{U} \right) \right)\sqrt{\mathbf{S}}\mathbf{R}^{\textrm T}\\ {\tilde{\mathbf{U}}} \left(e^{{\tilde{\mathbf{S}}}\frac{t}{\tau}} \left({\tilde{\mathbf{V}}}^{\textrm T}\mathbf{V} + {\tilde{\mathbf{U}}}^{\textrm T}\mathbf{U} \right) - e^{-{\tilde{\mathbf{S}}}\frac{t}{\tau}} \left({\tilde{\mathbf{V}}}^{\textrm T}\mathbf{V} - {\tilde{\mathbf{U}}}^{\textrm T}\mathbf{U} \right) \right)\sqrt{\mathbf{S}}\mathbf{R}^{\textrm T}\\ \end{bmatrix}\text{.} \end{align*}

We continue by calculating

\begin{align*} 4\mathbf{M}\mathbf{M}^{\textrm T} \mathbf{Q}\left(0\right) &amp; = 4\frac{1}{\sqrt{2}}\begin{bmatrix} {\tilde{\mathbf{V}}_\perp}\\ {\tilde{\mathbf{U}}_\perp}\\ \end{bmatrix} \frac{1}{\sqrt{2}}\begin{bmatrix} {\tilde{\mathbf{V}}_\perp}\\ {\tilde{\mathbf{U}}_\perp}\\ \end{bmatrix}^{\textrm T} \begin{bmatrix} \mathbf{V} \sqrt{\mathbf{S}}\mathbf{R}^{\textrm T}\\\ \mathbf{U} \sqrt{\mathbf{S}}\mathbf{R}^{\textrm T}\\ \end{bmatrix} \end{align*}

\begin{align*} &amp; = 2\begin{bmatrix} {\tilde{\mathbf{V}}_\perp} {\tilde{\mathbf{V}}_\perp}^{\textrm T} &amp; {\tilde{\mathbf{V}}_\perp} {\tilde{\mathbf{U}}_\perp}^{\textrm T} \\ {\tilde{\mathbf{U}}_\perp} {\tilde{\mathbf{V}}_\perp}^{\textrm T} &amp; {\tilde{\mathbf{U}}_\perp} {\tilde{\mathbf{U}}_\perp}^{\textrm T} \\ \end{bmatrix} \begin{bmatrix} \mathbf{V} \sqrt{\mathbf{S}}\mathbf{R}^{\textrm T}\\\ \mathbf{U} \sqrt{\mathbf{S}}\mathbf{R}^{\textrm T}\\ \end{bmatrix} \end{align*}

\begin{align*} &amp; = 2\begin{bmatrix} {\tilde{\mathbf{V}}_\perp} {\tilde{\mathbf{V}}_\perp}^{\textrm T} &amp; 0 \\ 0 &amp; {\tilde{\mathbf{U}}_\perp} {\tilde{\mathbf{U}}_\perp}^{\textrm T} \\ \end{bmatrix} \begin{bmatrix} \mathbf{V} \sqrt{\mathbf{S}}\mathbf{R}^{\textrm T}\\\ \mathbf{U} \sqrt{\mathbf{S}}\mathbf{R}^{\textrm T}\\ \end{bmatrix} \end{align*}

\begin{align*} &amp; = 2\begin{bmatrix} {\tilde{\mathbf{V}}_\perp} {\tilde{\mathbf{V}}_\perp}^{\textrm T} \mathbf{V} \sqrt{\mathbf{S}}\mathbf{R}^{\textrm T}\\ {\tilde{\mathbf{U}}_\perp} {\tilde{\mathbf{U}}_\perp}^{\textrm T} \mathbf{U} \sqrt{\mathbf{S}}\mathbf{R}^{\textrm T} \\ \end{bmatrix} \end{align*}

and

\begin{align*} \frac{1}{2}\mathbf{Q}\left(0\right)^{\textrm T} 4 \frac{t}{\tau} \mathbf{M}\mathbf{M}^{\textrm T} \mathbf{Q}\left(0\right) &amp; = \frac{t}{\tau} \begin{bmatrix} \mathbf{R}\sqrt{\mathbf{S}}\mathbf{V}^{\textrm T} \mathbf{R}\sqrt{\mathbf{S}}\mathbf{U}^{\textrm T} \end{bmatrix} \begin{bmatrix} {\tilde{\mathbf{V}}_\perp} {\tilde{\mathbf{V}}_\perp}^{\textrm T} \mathbf{V} \sqrt{\mathbf{S}}\mathbf{R}^{\textrm T}\\ {\tilde{\mathbf{U}}_\perp} {\tilde{\mathbf{U}}_\perp}^{\textrm T} \mathbf{U} \sqrt{\mathbf{S}}\mathbf{R}^{\textrm T} \\ \end{bmatrix} \end{align*}

\begin{align*} &amp; = \frac{t}{\tau} \begin{bmatrix} \mathbf{R}\sqrt{\mathbf{S}} \left(\mathbf{V}^{\textrm T}{\tilde{\mathbf{V}}_\perp} {\tilde{\mathbf{V}}_\perp}^{\textrm T} \mathbf{V} + \mathbf{U}^{\textrm T} {\tilde{\mathbf{U}}_\perp} {\tilde{\mathbf{U}}_\perp}^{\textrm T} \mathbf{U} \right) \sqrt{\mathbf{S}}\mathbf{R}^{\textrm T} \end{bmatrix} \end{align*}

Next, we define $\mathbf{B} = \mathbf{U}^{T}{\tilde{\mathbf{U}}} +\mathbf{V}^{T}\mathbf{\tilde{V}}$ and $\mathbf{C} = \mathbf{U}^{T}{\tilde{\mathbf{U}}} -\mathbf{V}^{T}\mathbf{\tilde{V}}$ and rewrite the inverse as

\begin{align*} &amp;\left[\mathbf{I} + \frac{1}{2}\mathbf{Q}\left(0\right)^{\textrm T}\mathbf{O} \left(e^{2\boldsymbol{\Lambda}\frac{t}{\tau}} - \mathbf{I}\right) \boldsymbol{\Lambda}^{-1} \mathbf{O}^{\textrm T} \mathbf{Q}\left(0\right) + 2\frac{t}{\tau} \mathbf{Q}\left(0\right)^{\textrm T} \mathbf{M}\mathbf{M}^{\textrm T} \mathbf{Q}\left(0\right) \right]^{-1} \end{align*}

\begin{align*} &amp; \quad = \ \Bigg[\mathbf{I} + \frac{1}{4}\mathbf{R}\sqrt{\mathbf{S}} \Bigg(\begin{bmatrix}\mathbf{B} &amp; -\mathbf{C}\end{bmatrix} \left(e^{2\boldsymbol{\Lambda}\frac{t}{\tau}} - \mathbf{I}\right) \boldsymbol{\Lambda}^{-1} \begin{bmatrix}\mathbf{B}^{\textrm T} \\ -\mathbf{C}^{\textrm T}\end{bmatrix} \nonumber \\ &amp; \qquad + 4\frac{t}{\tau} \left(\mathbf{V}^{\textrm T}{\tilde{\mathbf{V}}_\perp} {\tilde{\mathbf{V}}_\perp}^{\textrm T} \mathbf{V} + \mathbf{U}^{\textrm T} {\tilde{\mathbf{U}}_\perp} {\tilde{\mathbf{U}}_\perp}^{\textrm T} \mathbf{U} \right) \Bigg)\sqrt{\mathbf{S}}\mathbf{R}^{\textrm T}\Bigg]^{-1}\text{.} \end{align*}

Working from the centre out, we have

\begin{align*} \begin{bmatrix}\mathbf{B} &amp; -\mathbf{C}\end{bmatrix} \boldsymbol{\Lambda}^{-1} \begin{bmatrix}\mathbf{B}^{\textrm T} \\ -\mathbf{C}^{\textrm T}\end{bmatrix} &amp; = \begin{bmatrix}\mathbf{B} &amp; -\mathbf{C}\end{bmatrix} \begin{bmatrix}{\tilde{\mathbf{S}}}^{-1} &amp; 0\\ 0 &amp; -{\tilde{\mathbf{S}}}^{-1}\end{bmatrix} \begin{bmatrix}\mathbf{B}^{\textrm T} \\ -\mathbf{C}^{\textrm T}\end{bmatrix} \end{align*}

\begin{align*} &amp; = \begin{bmatrix}\mathbf{B} &amp; -\mathbf{C}\end{bmatrix} \begin{bmatrix}{\tilde{\mathbf{S}}}^{-1}\mathbf{B}^{\textrm T} \\ {\tilde{\mathbf{S}}}^{-1}\mathbf{C}^{\textrm T}\end{bmatrix} \end{align*}

\begin{align*} &amp; = \mathbf{B}{\tilde{\mathbf{S}}}^{-1}\mathbf{B}^{\textrm T} - \mathbf{C}{\tilde{\mathbf{S}}}^{-1}\mathbf{C}^{\textrm T} \end{align*}

and

\begin{align*} \begin{bmatrix}\mathbf{B} &amp; -\mathbf{C}\end{bmatrix} e^{2\boldsymbol{\Lambda}\frac{t}{\tau}} \boldsymbol{\Lambda}^{-1} \begin{bmatrix}\mathbf{B}^{\textrm T} \\ -\mathbf{C}^{\textrm T}\end{bmatrix} &amp; = \begin{bmatrix}\mathbf{B} &amp; -\mathbf{C}\end{bmatrix} \begin{bmatrix}e^{2{\tilde{\mathbf{S}}}\frac{t}{\tau}}{\tilde{\mathbf{S}}}^{-1} &amp; 0\\ 0 &amp; -e^{-2{\tilde{\mathbf{S}}}\frac{t}{\tau}}{\tilde{\mathbf{S}}}^{-1}\end{bmatrix} \begin{bmatrix}\mathbf{B}^{\textrm T} \\ -\mathbf{C}^{\textrm T}\end{bmatrix} \end{align*}

\begin{align*} &amp; = \begin{bmatrix}\mathbf{B} &amp; -\mathbf{C}\end{bmatrix} \begin{bmatrix}e^{2{\tilde{\mathbf{S}}}\frac{t}{\tau}}{\tilde{\mathbf{S}}}^{-1}\mathbf{B}^{\textrm T} \\ e^{-2{\tilde{\mathbf{S}}}\frac{t}{\tau}}{\tilde{\mathbf{S}}}^{-1}\mathbf{C}^{\textrm T}\end{bmatrix} \end{align*}

\begin{align*} &amp; = \mathbf{B}e^{2{\tilde{\mathbf{S}}}\frac{t}{\tau}}{\tilde{\mathbf{S}}}^{-1}\mathbf{B}^{\textrm T} - \mathbf{C}e^{-2{\tilde{\mathbf{S}}}\frac{t}{\tau}}{\tilde{\mathbf{S}}}^{-1}\mathbf{C}^{\textrm T}\text{.} \end{align*}

Finally, using $AB^{-1} = (BA^{-1})^{-1}$ (and $A^{-1}B = (B^{-1}A)^{-1}$) to move terms into the inverse, we rewrite

\begin{align*} \mathbf{Q}\mathbf{Q}^{\textrm T}(t) &amp; = \frac{1}{2} \begin{bmatrix} \left({\tilde{\mathbf{V}}} \left(e^{{\tilde{\mathbf{S}}}\frac{t}{\tau}} \mathbf{B}^{\textrm T} - e^{-{\tilde{\mathbf{S}}}\frac{t}{\tau}} \mathbf{C}^{\textrm T} \right) + 2{\tilde{\mathbf{V}}_\perp} {\tilde{\mathbf{V}}_\perp}^{\textrm T} \mathbf{V}\right) \sqrt{\mathbf{S}}\mathbf{R}^{\textrm T}\\ \left({\tilde{\mathbf{U}}} \left(e^{{\tilde{\mathbf{S}}}\frac{t}{\tau}} \mathbf{B}^{\textrm T} + e^{-{\tilde{\mathbf{S}}}\frac{t}{\tau}} \mathbf{C}^{\textrm T} \right) + 2{\tilde{\mathbf{U}}_\perp} {\tilde{\mathbf{U}}_\perp}^{\textrm T} \mathbf{U}\right) \sqrt{\mathbf{S}}\mathbf{R}^{\textrm T}\\ \end{bmatrix} \nonumber\\ &amp; \quad \times \bigg[\mathbf{I} + \mathbf{R}\sqrt{\mathbf{S}} \bigg(\frac{1}{4}\mathbf{B}\left(e^{2{\tilde{\mathbf{S}}}\frac{t}{\tau}} - \mathbf{I}\right){\tilde{\mathbf{S}}}^{-1}\mathbf{B}^{\textrm T} - \frac{1}{4}\mathbf{C}\left(e^{-2{\tilde{\mathbf{S}}}\frac{t}{\tau}} - \mathbf{I}\right){\tilde{\mathbf{S}}}^{-1}\mathbf{C}^{\textrm T}\nonumber\\ &amp; \quad + \frac{t}{\tau} \left(\mathbf{V}^{\textrm T}{\tilde{\mathbf{V}}_\perp} {\tilde{\mathbf{V}}_\perp}^{\textrm T} \mathbf{V} + \mathbf{U}^{\textrm T} {\tilde{\mathbf{U}}_\perp} {\tilde{\mathbf{U}}_\perp}^{\textrm T} \mathbf{U} \right) \bigg)\sqrt{\mathbf{S}}\mathbf{R}^{\textrm T}\bigg]^{-1} \end{align*}

\begin{align*} &amp;\frac{1}{2} \begin{bmatrix} \left({\tilde{\mathbf{V}}} \left(e^{{\tilde{\mathbf{S}}}\frac{t}{\tau}} \mathbf{B}^{\textrm T} - e^{-{\tilde{\mathbf{S}}}\frac{t}{\tau}} \mathbf{C}^{\textrm T} \right) + 2{\tilde{\mathbf{V}}_\perp} {\tilde{\mathbf{V}}_\perp}^{\textrm T} \mathbf{V}\right) \sqrt{\mathbf{S}}\mathbf{R}^{\textrm T}\\ \left({\tilde{\mathbf{U}}} \left(e^{{\tilde{\mathbf{S}}}\frac{t}{\tau}} \mathbf{B}^{\textrm T} + e^{-{\tilde{\mathbf{S}}}\frac{t}{\tau}} \mathbf{C}^{\textrm T} \right) + 2{\tilde{\mathbf{U}}_\perp} {\tilde{\mathbf{U}}_\perp}^{\textrm T} \mathbf{U}\right) \sqrt{\mathbf{S}}\mathbf{R}^{\textrm T}\\ \end{bmatrix}^{\textrm T} \nonumber\\ &amp;\quad = \frac{1}{2} \begin{bmatrix} {\tilde{\mathbf{V}}} \left(e^{{\tilde{\mathbf{S}}}\frac{t}{\tau}} \mathbf{B}^{\textrm T} - e^{-{\tilde{\mathbf{S}}}\frac{t}{\tau}} \mathbf{C}^{\textrm T} \right) + 2{\tilde{\mathbf{V}}_\perp} {\tilde{\mathbf{V}}_\perp}^{\textrm T} \mathbf{V}\\ {\tilde{\mathbf{U}}} \left(e^{{\tilde{\mathbf{S}}}\frac{t}{\tau}} \mathbf{B}^{\textrm T} + e^{-{\tilde{\mathbf{S}}}\frac{t}{\tau}} \mathbf{C}^{\textrm T} \right) + 2{\tilde{\mathbf{U}}_\perp} {\tilde{\mathbf{U}}_\perp}^{\textrm T} \mathbf{U}\\ \end{bmatrix} \nonumber\\ &amp; \qquad \times \left[\mathbf{S}^{-1} + \frac{1}{4} \mathbf{B}\left(e^{2{\tilde{\mathbf{S}}}\frac{t}{\tau}} - \mathbf{I}\right){\tilde{\mathbf{S}}}^{-1}\mathbf{B}^{\textrm T} - \frac{1}{4} \mathbf{C}\left(e^{-2{\tilde{\mathbf{S}}}\frac{t}{\tau}} - \mathbf{I}\right){\tilde{\mathbf{S}}}^{-1}\mathbf{C}^{\textrm T}\right.\nonumber\\ &amp; \qquad \left. + \frac{t}{\tau} \left(\mathbf{V}^{\textrm T}{\tilde{\mathbf{V}}_\perp} {\tilde{\mathbf{V}}_\perp}^{\textrm T} \mathbf{V} + \mathbf{U}^{\textrm T} {\tilde{\mathbf{U}}_\perp} {\tilde{\mathbf{U}}_\perp}^{\textrm T} \mathbf{U} \right) \right]^{-1} \end{align*}

\begin{align*} &amp;\frac{1}{2} \begin{bmatrix} {\tilde{\mathbf{V}}} \left(e^{{\tilde{\mathbf{S}}}\frac{t}{\tau}} \mathbf{B}^{\textrm T} - e^{-{\tilde{\mathbf{S}}}\frac{t}{\tau}} \mathbf{C}^{\textrm T} \right) + 2{\tilde{\mathbf{V}}_\perp} {\tilde{\mathbf{V}}_\perp}^{\textrm T} \mathbf{V}\\ {\tilde{\mathbf{U}}} \left(e^{{\tilde{\mathbf{S}}}\frac{t}{\tau}} \mathbf{B}^{\textrm T} + e^{-{\tilde{\mathbf{S}}}\frac{t}{\tau}} \mathbf{C}^{\textrm T} \right) + 2{\tilde{\mathbf{U}}_\perp} {\tilde{\mathbf{U}}_\perp}^{\textrm T} \mathbf{U}\\ \end{bmatrix}^{\textrm T} \nonumber\\ &amp;\quad = \begin{bmatrix} {\tilde{\mathbf{V}}} \left(\mathbf{I} - e^{-{\tilde{\mathbf{S}}}\frac{t}{\tau}} \mathbf{C}^{\textrm T} \left(\mathbf{B}^{T}\right)^{-1} e^{-{\tilde{\mathbf{S}}}\frac{t}{\tau}} \right) + 2{\tilde{\mathbf{V}}_\perp} {\tilde{\mathbf{V}}_\perp}^{\textrm T} \mathbf{V} \left(\mathbf{B}^{T}\right)^{-1} e^{-{\tilde{\mathbf{S}}}\frac{t}{\tau}}\\ {\tilde{\mathbf{U}}} \left(\mathbf{I} + e^{-{\tilde{\mathbf{S}}}\frac{t}{\tau}} \mathbf{C}^{\textrm T} \left(\mathbf{B}^{T}\right)^{-1} e^{-{\tilde{\mathbf{S}}}\frac{t}{\tau}} \right) + 2{\tilde{\mathbf{U}}_\perp} {\tilde{\mathbf{U}}_\perp}^{\textrm T} \mathbf{U} \left(\mathbf{B}^{T}\right)^{-1} e^{-{\tilde{\mathbf{S}}}\frac{t}{\tau}}\\ \end{bmatrix} \nonumber\\ &amp; \qquad \times \bigg[4 e^{-{\tilde{\mathbf{S}}}\frac{t}{\tau}} \mathbf{B}^{-1}\mathbf{S}^{-1} \left(\mathbf{B}^{T}\right)^{-1} e^{-{\tilde{\mathbf{S}}}\frac{t}{\tau}} + \left(\mathbf{I} - e^{-2{\tilde{\mathbf{S}}}\frac{t}{\tau}}\right){\tilde{\mathbf{S}}}^{-1} \nonumber\\ &amp; \qquad - e^{-{\tilde{\mathbf{S}}}\frac{t}{\tau}} \mathbf{B}^{-1}\mathbf{C}\left(e^{-2{\tilde{\mathbf{S}}}\frac{t}{\tau}} - \mathbf{I}\right){\tilde{\mathbf{S}}}^{-1}\mathbf{C}^{\textrm T} \left(\mathbf{B}^{T}\right)^{-1} e^{-{\tilde{\mathbf{S}}}\frac{t}{\tau}}\nonumber\\ &amp; \qquad + 4\frac{t}{\tau} e^{-{\tilde{\mathbf{S}}}\frac{t}{\tau}} \mathbf{B}^{-1} \left(\mathbf{V}^{\textrm T}{\tilde{\mathbf{V}}_\perp} {\tilde{\mathbf{V}}_\perp}^{\textrm T} \mathbf{V} + \mathbf{U}^{\textrm T} {\tilde{\mathbf{U}}_\perp} {\tilde{\mathbf{U}}_\perp}^{\textrm T} \mathbf{U} \right) \left(\mathbf{B}^{T}\right)^{-1} e^{-{\tilde{\mathbf{S}}}\frac{t}{\tau}} \bigg]^{-1} \nonumber \\ &amp;\qquad \times \begin{bmatrix} {\tilde{\mathbf{V}}} \left(\mathbf{I} - e^{-{\tilde{\mathbf{S}}}\frac{t}{\tau}} \mathbf{C}^{\textrm T} \left(\mathbf{B}^{T}\right)^{-1} e^{-{\tilde{\mathbf{S}}}\frac{t}{\tau}} \right) + 2{\tilde{\mathbf{V}}_\perp} {\tilde{\mathbf{V}}_\perp}^{\textrm T} \mathbf{V} B^{-T} e^{-{\tilde{\mathbf{S}}}\frac{t}{\tau}}\\ {\tilde{\mathbf{U}}} \left(\mathbf{I} + e^{-{\tilde{\mathbf{S}}}\frac{t}{\tau}} \mathbf{C}^{\textrm T} \left(\mathbf{B}^{T}\right)^{-1} e^{-{\tilde{\mathbf{S}}}\frac{t}{\tau}} \right) + 2{\tilde{\mathbf{U}}_\perp} {\tilde{\mathbf{U}}_\perp}^{\textrm T} \mathbf{U} B^{-T} e^{-{\tilde{\mathbf{S}}}\frac{t}{\tau}}\\ \end{bmatrix}^{\textrm T}. \end{align*}

### C.3. Proof of theorem 3.2: Limiting behaviour

As training time increases, all terms including a matrix exponential with negative exponent in equation (11) vanish to zero, as ${\tilde{\mathbf{S}}}$ is a diagonal matrix with entries larger zero

\begin{equation*} \lim_{t\to\infty} e^{-{\tilde{\mathbf{S}}}\frac{t}{\tau}} = \mathbf{0}\text{.} \end{equation*}

Therefore, in the temporal limit, equation (11) reduces to

\begin{align*} \lim_{t\to\infty}\mathbf{Q}\mathbf{Q}^{\textrm T}\left(t\right) &amp; = \lim_{t\to\infty} \begin{bmatrix} \mathbf{W}_1^{\textrm T} \mathbf{W}_1\left(t\right) &amp; \mathbf{W}_1^{\textrm T}\mathbf{W}_2^{\textrm T}\left(t\right) \\ \mathbf{W}_2^{} \mathbf{W}_1\left(t\right) &amp; \mathbf{W}_2^{\textrm T} \mathbf{W}_2\left(t\right) \end{bmatrix} \end{align*}

\begin{align*} &amp; = \begin{bmatrix} {\tilde{\mathbf{V}}} \\ {\tilde{\mathbf{U}}} \end{bmatrix} \begin{bmatrix} {\tilde{\mathbf{S}}}^{-1} \end{bmatrix}^{-1} \begin{bmatrix} {\tilde{\mathbf{V}}}^{\textrm T} &amp; {\tilde{\mathbf{U}}}^{\textrm T} \end{bmatrix} \end{align*}

\begin{align*} &amp; = \begin{bmatrix} {\tilde{\mathbf{V}}} {\tilde{\mathbf{S}}} {\tilde{\mathbf{V}}}^{\textrm T} &amp; {\tilde{\mathbf{V}}} {\tilde{\mathbf{S}}} {\tilde{\mathbf{U}}}^{\textrm T} \\ {\tilde{\mathbf{U}}} {\tilde{\mathbf{S}}} {\tilde{\mathbf{V}}}^{\textrm T} &amp; {\tilde{\mathbf{U}}} {\tilde{\mathbf{S}}} {\tilde{\mathbf{U}}}^{\textrm T} \end{bmatrix}. \end{align*}

$\square$

### C.4. Dynamics of $\mathbf{Q} {\mathbf{(t)}}$

The solution for the weights $\mathbf{W}_1(t)$ and $\mathbf{W}_2(t)$ can be derived up to a time varying orthogonal transformation as demonstrated by Yan *et al* (1994).

Under the assumptions of whitened inputs 2.2, zero-balanced weights 2.3, full rank 2.4, and equal input-output dimension, the temporal dynamics of $\mathbf{Q} (t)$ is given as

\begin{align*} \mathbf{Q} \left(t\right) = e^{\mathbf{F}\frac{t}{\tau}}\mathbf{Q}\left(0\right)\left[\mathbf{I} + \frac{1}{2}\mathbf{Q}\left(0\right)^{\textrm T}\left(e^{\mathbf{F}\frac{t}{\tau}}\mathbf{F}^{-1}e^{\mathbf{F}\frac{t}{\tau}} - \mathbf{F}^{-1} \right) \mathbf{Q}\left(0\right)\right]^{-\frac{1}{2}} \mathbf{D}\left(t\right)\text{.} \end{align*}

where ${\mathbf{D}}(t)$ is an orthogonal matrix of size $N_\textrm h\times N_\textrm h$. From this definition, computing $\mathbf{Q} (t)\mathbf{Q} (t)^{\textrm T}$, we recover equation (45).

Equation (157) shows that the individual weight matrices are not directly described by parts of the $\mathbf{Q} (t)\mathbf{Q} (t)^{\textrm T}$ solution. Instead, they are fixed only up to a time-dependent orthogonal transformation. To verify this, we numerically compute ${\mathbf{D}}(t)$ as ${\mathbf{D}}(t) = {\mathbf{q}}(t)^{+}{\mathbf{Q}}_{\textrm{sim}}(t)$ where ${\mathbf{Q}}_{\textrm{sim}}(t)$ denotes weights obtained from numerical simulations of gradient descent, + denotes the pseudoinverse ( ${\mathbf{q}}^{+}(t) = ({\mathbf{q}}^{\textrm T}(t) {\mathbf{q}}(t))^{-1}\mathbf{{q}(t)}^{\textrm T}$ where $\mathbf{q}(t)$ is rectangular) and

\begin{align*} \mathbf{q}\left(t\right) = e^{\mathbf{F}\frac{t}{\tau}}\mathbf{Q}\left(0\right)\left[\mathbf{I} + \frac{1}{2}\mathbf{Q}\left(0\right)^{\textrm T}\left(e^{\mathbf{F}\frac{t}{\tau}}\mathbf{F}^{-1}e^{\mathbf{F}\frac{t}{\tau}} - \mathbf{F}^{-1} \right) \mathbf{Q}\left(0\right)\right]^{-\frac{1}{2}}\text{.} \end{align*}

We numerically show in figure 7(D) right panel that $\mathbf{{D}}(t)$ generally changes over time. Letting $\mathbf{Q}_d (t)$ denote the estimated $\mathbf{Q} (t)$ using the numerically recovered $\mathbf{{D}}(t)$, figure 7(D) left and centre panels show that both the dynamics of $\mathbf{Q}_d (t)$ and $\mathbf{Q}_d (t)\mathbf{Q}_d (t)^{\textrm T}$ match the temporal dynamics of the simulation. The small derivation between the simulation and the analytical solution for later time points, is due to the imprecision of the pseudoinverse.

In figure 7(C), we report the implementation of equation (158). As expected, the analytical solution does not match the numerical temporal dynamics. However, the solution for $\mathbf{q}(t)\mathbf{q}(t)^{\textrm T}$ recovers the correct dynamics.

## Appendix D Rich and lazy learning regimes and generalisation

Under the assumptions of theorem 3.1, the network function acquires a rich task-specific internal representation at convergence, that is $\mathbf{W}_1^{\textrm T}\mathbf{W}_1 = \mathbf{\tilde{V}}{\tilde{\mathbf{S}}}\mathbf{\tilde{V}}^{\textrm T}$ and $\mathbf{W}_2\mathbf{W}_2^{\textrm T} = {\tilde{\mathbf{U}}}{\tilde{\mathbf{S}}}{\tilde{\mathbf{U}}}^{\textrm T}$. Therefore, there exist initial states with large zero-balanced weights that lead to rich solutions.

We more quantitatively capture this phenomena in figure 8. We define the error on the internal representation as figure 3 $|| \mathbf{W}_1^{\textrm T}\mathbf{W}_1 - \mathbf{\tilde{V}}{\tilde{\mathbf{S}}}\mathbf{\tilde{V}}^{\textrm T}||_F^2$ and $||\mathbf{W}_2\mathbf{W}_2^{\textrm T} -{\tilde{\mathbf{U}}}{\tilde{\mathbf{S}}}{\tilde{\mathbf{U}}}^{\textrm T}|_F^2$ for **W**_1_ and **W**_2_ respectively. Effectively, we measure the richness of the representation and in turn it is generalisation ability. In figure 8, the error remains zero for increasing gain for any network initialised with zero-balanced weights. In other words, the representation at convergences is rich. In contrast, for random initialisation the error increase consequently with increasing gain. As the network is moving away from the small random weight initialisation, the network converges to lazier representation.

## Appendix E Decoupling dynamics

### E.1. Proof for theorem 5.1

Let the input and output dimension of a two-layer linear network (equation (1)) be equal, i.e. $N_\textrm i = N_\textrm o$, then equation (11) simplifies to

\begin{align*} \mathbf{Q}\mathbf{Q}^{\textrm T}\left(t\right) &amp;= \begin{bmatrix} {\tilde{\mathbf{V}}} \left(\mathbf{I} - e^{-{\tilde{\mathbf{S}}}\frac{t}{\tau}} \mathbf{C}^{\textrm T} \left(\mathbf{B}^{T}\right)^{-1} e^{-{\tilde{\mathbf{S}}}\frac{t}{\tau}} \right) \\ {\tilde{\mathbf{U}}} \left(\mathbf{I} + e^{-{\tilde{\mathbf{S}}}\frac{t}{\tau}} \mathbf{C}^{\textrm T} \left(\mathbf{B}^{T}\right)^{-1} e^{-{\tilde{\mathbf{S}}}\frac{t}{\tau}} \right) \\ \end{bmatrix} \nonumber\\ &amp; \quad \times \left[4 e^{-{\tilde{\mathbf{S}}}\frac{t}{\tau}} \mathbf{B}^{-1}\mathbf{S}^{-1} \left(\mathbf{B}^{T}\right)^{-1} e^{-{\tilde{\mathbf{S}}}\frac{t}{\tau}} + \left(\mathbf{I} - e^{-2{\tilde{\mathbf{S}}}\frac{t}{\tau}}\right){\tilde{\mathbf{S}}}^{-1}\right. \nonumber\\ &amp;\quad \left. - e^{-{\tilde{\mathbf{S}}}\frac{t}{\tau}} \mathbf{B}^{-1}\mathbf{C}\left(e^{-2{\tilde{\mathbf{S}}}\frac{t}{\tau}} - \mathbf{I}\right){\tilde{\mathbf{S}}}^{-1}\mathbf{C}^{\textrm T} \left(\mathbf{B}^{T}\right)^{-1} e^{-{\tilde{\mathbf{S}}}\frac{t}{\tau}}\right]^{-1} \nonumber\\ &amp;\quad \times \begin{bmatrix} {\tilde{\mathbf{V}}} \left(\mathbf{I} - e^{-{\tilde{\mathbf{S}}}\frac{t}{\tau}} \mathbf{C}^{\textrm T} \left(\mathbf{B}^{T}\right)^{-1} e^{-{\tilde{\mathbf{S}}}\frac{t}{\tau}} \right)\\ {\tilde{\mathbf{U}}} \left(\mathbf{I} + e^{-{\tilde{\mathbf{S}}}\frac{t}{\tau}} \mathbf{C}^{\textrm T} \left(\mathbf{B}^{T}\right)^{-1} e^{-{\tilde{\mathbf{S}}}\frac{t}{\tau}} \right)\\ \end{bmatrix}^{\textrm T}. \end{align*}

Further, let the singular value decomposition of the input-output correlation of the task be

\begin{equation*} \mathrm{SVD}\left(\boldsymbol{\tilde{\Sigma}}^{yx}\right) = {\tilde{\mathbf{U}}}{\tilde{\mathbf{S}}}{\tilde{\mathbf{V}}}^{\textrm T} \end{equation*}

and suppose that the initial state of the network can be written in the form

\begin{equation*} \mathrm{SVD}\left(\mathbf{W}_2\left(0\right)\mathbf{W}_1\left(0\right)\right) = \mathbf{U}\mathbf{S}\mathbf{V}^{\textrm T} = {\tilde{\mathbf{U}}}\mathbf{A}\left(0\right)^{\textrm T}\mathbf{A}\left(0\right){\tilde{\mathbf{V}}}^{\textrm T} \text{.} \end{equation*}

First, we note that the initial weights in this setting are not independent of the structure of the target task. In particular,

\begin{align*} \mathbf{U}\sqrt{\mathbf{S}} &amp; = {\tilde{\mathbf{U}}}\mathbf{A}\left(0\right)^{\textrm T} \end{align*}

\begin{align*} \Leftrightarrow {\tilde{\mathbf{U}}}^{\textrm T}\mathbf{U}\sqrt{\mathbf{S}} &amp; = \mathbf{A}\left(0\right)^{\textrm T} \end{align*}

\begin{align*} \Leftrightarrow \sqrt{\mathbf{S}}\mathbf{U}^{\textrm T}{\tilde{\mathbf{U}}} &amp; = \mathbf{A}\left(0\right) \end{align*}

and

\begin{align*} \sqrt{\mathbf{S}}\mathbf{V}^{\textrm T} &amp; = \mathbf{A}\left(0\right){\tilde{\mathbf{V}}}^{\textrm T} \end{align*}

\begin{align*} \Leftrightarrow \sqrt{\mathbf{S}}\mathbf{V}^{\textrm T}{\tilde{\mathbf{V}}} &amp; = \mathbf{A}\left(0\right) \end{align*}

and therefore

\begin{align*} \sqrt{\mathbf{S}}\mathbf{U}^{\textrm T}{\tilde{\mathbf{U}}} &amp; = \sqrt{\mathbf{S}}\mathbf{V}^{\textrm T}{\tilde{\mathbf{V}}} \end{align*}

\begin{align*} \Leftrightarrow \mathbf{U}\mathbf{V}^{\textrm T} &amp; = {\tilde{\mathbf{U}}}{\tilde{\mathbf{V}}}^{\textrm T}\text{.} \end{align*}

This further simplifies the equation, as

\begin{align*} \mathbf{U}\sqrt{\mathbf{S}} &amp; = {\tilde{\mathbf{U}}}\mathbf{A}\left(0\right)^{\textrm T} \end{align*}

\begin{align*} \Leftrightarrow \mathbf{U} &amp; = {\tilde{\mathbf{U}}}\mathbf{A}\left(0\right)^{\textrm T}\sqrt{\mathbf{S}}^{-1} \end{align*}

and

\begin{align*} \sqrt{\mathbf{S}}\mathbf{V}^{\textrm T} &amp; = \mathbf{A}\left(0\right){\tilde{\mathbf{V}}}^{\textrm T} \end{align*}

\begin{align*} \Leftrightarrow \mathbf{V}^{\textrm T} &amp; = \sqrt{\mathbf{S}}^{-1}\mathbf{A}\left(0\right){\tilde{\mathbf{V}}}^{\textrm T} \end{align*}

\begin{align*} \Leftrightarrow \mathbf{V} &amp; = {\tilde{\mathbf{V}}}\mathbf{A}\left(0\right)^{\textrm T}\sqrt{\mathbf{S}}^{-1} \text{,} \end{align*}

then recollecting the definition of **B** and **C** we get

\begin{align*} \mathbf{B}^{\textrm T} &amp; = {\tilde{\mathbf{U}}}^{\textrm T}\mathbf{U} + {\tilde{\mathbf{V}}}^{\textrm T}\mathbf{V} \end{align*}

\begin{align*} &amp; = {\tilde{\mathbf{U}}}^{\textrm T}{\tilde{\mathbf{U}}}\mathbf{A}\left(0\right)^{\textrm T}\sqrt{S}^{-1} + {\tilde{\mathbf{V}}}^{\textrm T}{\tilde{\mathbf{V}}}\mathbf{A}\left(0\right)^{\textrm T}\sqrt{S}^{-1} \end{align*}

\begin{align*} &amp; = \left({\tilde{\mathbf{U}}}^{\textrm T}{\tilde{\mathbf{U}}} + {\tilde{\mathbf{V}}}^{\textrm T}{\tilde{\mathbf{V}}}\right)\mathbf{A}\left(0\right)^{\textrm T}\sqrt{S}^{-1} \end{align*}

\begin{align*} &amp; = 2\mathbf{A}\left(0\right)^{\textrm T}\sqrt{S}^{-1} \end{align*}

and

\begin{align*} \mathbf{C}^{\textrm T} &amp; = {\tilde{\mathbf{U}}}^{\textrm T}\mathbf{U} - {\tilde{\mathbf{V}}}^{\textrm T}\mathbf{V} \end{align*}

\begin{align*} &amp; = \left({\tilde{\mathbf{U}}}^{\textrm T}{\tilde{\mathbf{U}}} - {\tilde{\mathbf{V}}}^{\textrm T}{\tilde{\mathbf{V}}}\right)\mathbf{A}\left(0\right)^{\textrm T}\sqrt{S}^{-1} \end{align*}

\begin{align*} &amp; = 0\text{.} \end{align*}

Substituting the new values of **B** and **C** into equation (159) then yields

\begin{align*} \mathbf{Q}\mathbf{Q}^{\textrm T}\left(t\right) &amp; = \begin{bmatrix} {\tilde{\mathbf{V}}} \\ {\tilde{\mathbf{U}}} \\ \end{bmatrix} \left[4 e^{-{\tilde{\mathbf{S}}}\frac{t}{\tau}} \frac{1}{4}\mathbf{A}\left(0\right)^{-1} \sqrt{\mathbf{S}} \mathbf{S}^{-1} \sqrt{\mathbf{S}} \mathbf{A}\left(0\right)^{-T} e^{-{\tilde{\mathbf{S}}}\frac{t}{\tau}} + \left(\mathbf{I} - e^{-2{\tilde{\mathbf{S}}}\frac{t}{\tau}}\right){\tilde{\mathbf{S}}}^{-1}\right]^{-1} \begin{bmatrix} {\tilde{\mathbf{V}}} \\ {\tilde{\mathbf{U}}} \\ \end{bmatrix}^{\textrm T} \end{align*}

\begin{align*} &amp; = \begin{bmatrix} {\tilde{\mathbf{V}}} \\ {\tilde{\mathbf{U}}} \\ \end{bmatrix} \left[e^{-{\tilde{\mathbf{S}}}\frac{t}{\tau}} \left(\mathbf{A}\left(0\right)^{T} \mathbf{A}\left(0\right)\right)^{-1} e^{-{\tilde{\mathbf{S}}}\frac{t}{\tau}} + \left(\mathbf{I} - e^{-2{\tilde{\mathbf{S}}}\frac{t}{\tau}}\right){\tilde{\mathbf{S}}}^{-1}\right]^{-1} \begin{bmatrix} {\tilde{\mathbf{V}}} \\ {\tilde{\mathbf{U}}} \\ \end{bmatrix}^{\textrm T}\text{.} \end{align*}

Finally, we note that the dynamics can thus be written as

\begin{equation*} \mathbf{Q}\mathbf{Q}^{\textrm T}\left(t\right) = \begin{bmatrix} {\tilde{\mathbf{V}}} \mathbf{A}^{\textrm T}\mathbf{A}\left(t\right) {\tilde{\mathbf{V}}}^{\textrm T} &amp; {\tilde{\mathbf{V}}} \mathbf{A}^{\textrm T}\mathbf{A}\left(t\right) {\tilde{\mathbf{U}}}^{\textrm T} \\ {\tilde{\mathbf{U}}} \mathbf{A}^{\textrm T}\mathbf{A}\left(t\right) {\tilde{\mathbf{V}}}^{\textrm T} &amp; {\tilde{\mathbf{U}}} \mathbf{A}^{\textrm T}\mathbf{A}\left(t\right) {\tilde{\mathbf{U}}}^{\textrm T} \end{bmatrix} \end{equation*}

where

\begin{equation*} \mathbf{A}^{\textrm T}\mathbf{A}\left(t\right) = \left[e^{-{\tilde{\mathbf{S}}}\frac{t}{\tau}} \left(\mathbf{A}\left(0\right)^{T} \mathbf{A}\left(0\right)\right)^{-1} e^{-{\tilde{\mathbf{S}}}\frac{t}{\tau}} + \left(\mathbf{I} - e^{-2{\tilde{\mathbf{S}}}\frac{t}{\tau}}\right){\tilde{\mathbf{S}}}^{-1}\right]^{-1} \text{.} \end{equation*}

$\square$

### E.2. Solution for $\boldsymbol{2\times 2}$ dynamics

We consider small networks with input and output dimension $N_\textrm i = 2$ and $N_\textrm o = 2$. In this setting, the structure of the weight initialisation and task are encoded in the matrices

\begin{equation*} \mathbf{A}\left(0\right)^{\textrm T}\mathbf{A}\left(0\right) = \begin{bmatrix} a_1\left(0\right) &amp; b\left(0\right) \\ b\left(0\right) &amp; a_2\left(0\right) \end{bmatrix} \text{and} \,{\tilde{\mathbf{S}}} = \begin{bmatrix} s_1 &amp; 0 \\ 0 &amp; s_2 \end{bmatrix}\text{,} \end{equation*}

where the parameters $a_1(0)$ and $a_2(0)$ represent coupling within a singular mode, and *b*(0) represents counterproductive cross-coupling between different singular modes.

From equation (13), we have

\begin{align*} \mathbf{A}^{\textrm T}\mathbf{A}\left(t\right) = &amp; \left[ \begin{bmatrix} e^{\frac{-s_1t}{\tau}} &amp; 0\\ 0 &amp; e^{\frac{-s_2t}{\tau}} \end{bmatrix} \begin{bmatrix} a_1\left(0\right) &amp; b\left(0\right) \\ b\left(0\right) &amp; a_2\left(0\right) \end{bmatrix}^{-1} \begin{bmatrix} e^{\frac{-s_1t}{\tau}} &amp; 0\\ 0 &amp; e^{\frac{-s_2t}{\tau}} \end{bmatrix} \right.\nonumber\\ &amp; \left.+ \left[ \begin{bmatrix} 1 &amp; 0 \\ 0 &amp; 1 \end{bmatrix}- \begin{bmatrix} e^{\frac{-2s_1t}{\tau}} &amp; 0\\ 0 &amp; e^{\frac{-2s_2t}{\tau}} \end{bmatrix} \right] \begin{bmatrix} s_1 &amp; 0 \\ 0 &amp; s_2 \end{bmatrix} ^{-1} \right]^{-1} \end{align*}

\begin{align*} = &amp;\left[ \frac{1}{a_1\left(0\right)a_2\left(0\right)-b\left(0\right)^2} \begin{bmatrix} e^{\frac{-s_1t}{\tau}} &amp; 0\\ 0 &amp; e^{\frac{-s_2t}{\tau}} \end{bmatrix} \begin{bmatrix} a_2\left(0\right) &amp; -b\left(0\right) \\ -b\left(0\right) &amp; a_1\left(0\right) \end{bmatrix} \begin{bmatrix} e^{\frac{-s_1t}{\tau}} &amp; 0\\ 0 &amp; e^{\frac{-s_2t}{\tau}} \end{bmatrix}\right. \nonumber\\ &amp;\left.+ \left[ \begin{bmatrix} 1 &amp; 0 \\ 0 &amp; 1 \end{bmatrix}- \begin{bmatrix} e^{\frac{-2s_1t}{\tau}} &amp; 0\\ 0 &amp; e^{\frac{-2s_2t}{\tau}} \end{bmatrix} \right] \begin{bmatrix} \frac{1}{s_1} &amp; 0 \\ 0 &amp; \frac{1}{s_2} \end{bmatrix} \right]^{-1}, \end{align*}

where we use

\begin{align*} \begin{bmatrix} a &amp; b \\ c &amp; d \\ \end{bmatrix}^{-1} = \frac{1}{ad - bc} \begin{bmatrix} \,\,\,d &amp; \!\!-b \\ -c &amp; \,a \\ \end{bmatrix} \text{.} \end{align*}

We continue with

\begin{align*} \begin{array}{l} {A^T}A\left( t \right) = \left[ {\frac{1}{{{a_1}\left( 0 \right){a_2}\left( 0 \right) - b{{\left( 0 \right)}^2}}}} \right.\left[ {\begin{array}{*{20}{c}} {{e^{\frac{{ - 2{s_1}t}}{\tau }}}}&amp;0\\ 0&amp;{{e^{\frac{{ - 2{s_2}t}}{\tau }}}} \end{array}} \right]\left[ {\begin{array}{*{20}{c}} {{a_2}\left( 0 \right)}&amp;{ - b\left( 0 \right)}\\ { - b\left( 0 \right)}&amp;{{a_1}\left( 0 \right)} \end{array}} \right]\left[ {\begin{array}{*{20}{c}} {{e^{\frac{{ - 2{s_1}t}}{\tau }}}}&amp;0\\ 0&amp;{{e^{\frac{{ - 2{s_2}t}}{\tau }}}} \end{array}} \right]\\ \,\,\,\,\,\,\,\,\,\,\,\,\,\,\,\,\,\,\,\,\,\,\,\,\,\ \ \ \quad + {\left. {\left[ {\begin{array}{*{20}{c}} {\frac{1}{{{s_1}}}}&amp;0\\ 0&amp;{\frac{1}{{{s_2}}}} \end{array}} \right] - \left[ {\begin{array}{*{20}{c}} {\frac{1}{{{s_1}}}{e^{\frac{{ - 2{s_1}t}}{\tau }}}}&amp;0\\ 0&amp;{\frac{1}{{{s_2}}}{e^{\frac{{ - 2{s_2}t}}{\tau }}}} \end{array}} \right]} \right]^{ - 1}} \end{array} \end{align*}

\begin{align*} \begin{array}{l} = \left[ {\frac{1}{{{a_1}\left( 0 \right){a_2}\left( 0 \right) - b{{\left( 0 \right)}^2}}}\left[ {\begin{array}{*{20}{c}} {{e^{\frac{{ - 2{s_1}t}}{\tau }}}{a_2}\left( 0 \right)}&amp;{ - {e^{\frac{{ - {s_1}t}}{\tau }}}b\left( 0 \right){e^{\frac{{ - {s_2}t}}{\tau }}}}\\ { - {e^{\frac{{ - {s_2}t}}{\tau }}}b\left( 0 \right){e^{\frac{{ - {s_1}t}}{\tau }}}}&amp;{{e^{\frac{{ - 2{s_2}t}}{\tau }}}{a_1}\left( 0 \right)} \end{array}} \right]} \right.\\ \,\,\,\,\,\ {\left. {\,\, + \left[ {\begin{array}{*{20}{c}} {\frac{1}{{{s_1}}}}&amp;0\\ 0&amp;{\frac{1}{{{s_2}}}} \end{array}} \right] - \left[ {\begin{array}{*{20}{c}} {\frac{1}{{{s_1}}}{e^{\frac{{ - 2{s_1}t}}{\tau }}}}&amp;0\\ 0&amp;{\frac{1}{{{s_2}}}{e^{\frac{{ - 2{s_2}t}}{\tau }}}} \end{array}} \right]} \right]^{ - 1}} \end{array} \end{align*}

\begin{align*} &amp;= \begin{bmatrix} \frac{e^{\frac{-2s_1t}{\tau}} a_2\left(0\right)}{a_1\left(0\right)a_2\left(0\right)-b\left(0\right)^2} + \frac{1}{s_1} - \frac{1}{s_1}e^{\frac{-2s_1t}{\tau}} &amp; -\frac{e^{\frac{-s_1t}{\tau}} b\left(0\right) e^{\frac{-s_2t}{\tau}} }{a_1\left(0\right)a_2\left(0\right)-b\left(0\right)^2} \\ - \frac{e^{\frac{-s_2t}{\tau}} b\left(0\right) e^{\frac{-s_1t}{\tau}}}{a_1\left(0\right)a_2\left(0\right)-b\left(0\right)^2} &amp; \frac{e^{\frac{-2s_2t}{\tau}} a_1\left(0\right)}{a_1\left(0\right)a_2\left(0\right)-b\left(0\right)^2} + \frac{1}{s_2} -\frac{1}{s_2}e^{\frac{-2s_2t}{\tau}} \end{bmatrix} ^{-1}. \end{align*}

We use equation (189) and simplify the denominator

\begin{align*} &amp;\mathbf{A}^{\textrm T}\mathbf{A}\left(t\right) \nonumber \\ &amp;\quad =\frac{1}{\left( \frac{e^{\frac{-2s_2t}{\tau}} a_1\left(0\right)}{a_1\left(0\right)a_2\left(0\right)-b\left(0\right)^2} + \frac{1}{s_2} -\frac{1}{s_2}e^{\frac{-2s_2t}{\tau}}\right)\left(\frac{e^{\frac{-2s_1t}{\tau}} a_2\left(0\right)}{a_1\left(0\right)a_2\left(0\right)-b\left(0\right)^2} + \frac{1}{s_1} - \frac{1}{s_1}e^{\frac{-2s_1t}{\tau}}\right)- \left(-\frac{e^{\frac{-s_2t}{\tau}} b\left(0\right) e^{\frac{-s_1t}{\tau}}}{a_1\left(0\right)a_2\left(0\right)-b\left(0\right)^2}\right)^2} \nonumber\\ &amp;\qquad \times \begin{bmatrix} \frac{e^{\frac{-2s_2t}{\tau}} a_1\left(0\right)}{a_1\left(0\right)a_2\left(0\right)-b\left(0\right)^2} + \frac{1}{s_2} -\frac{1}{s_2}e^{\frac{-2s_2t}{\tau}} &amp; \frac{e^{\frac{-s_1t}{\tau}} b\left(0\right) e^{\frac{-s_2t}{\tau}} }{a_1\left(0\right)a_2\left(0\right)-b\left(0\right)^2} \\[3pt] \frac{e^{\frac{-s_2t}{\tau}} b(0) e^{\frac{-s_1t}{\tau}}}{a_1(0)a_2(0)-b(0)^2} &amp; \frac{e^{\frac{-2s_1t}{\tau}} a_2(0)}{a_1(0)a_2(0)-b(0)^2} + \frac{1}{s_1} - \frac{1}{s_1}e^{\frac{-2s_1t}{\tau}} \end{bmatrix}. \end{align*}

The diagonal element $a_1(t)$ is given as

\begin{align*} &amp;a_1\left(t\right) \nonumber\\ &amp;\quad = \frac{ \frac{e^{\frac{-2s_2t}{\tau}} a_1\left(0\right)}{a_1\left(0\right)a_2\left(0\right)-b\left(0\right)^2} + \frac{1}{s_2} -\frac{1}{s_2}e^{\frac{-2s_2t}{\tau}}}{\left( \frac{e^{\frac{-2s_2t}{\tau}} a_1\left(0\right)}{a_1\left(0\right)a_2\left(0\right)-b\left(0\right)^2} + \frac{1}{s_2} -\frac{1}{s_2}e^{\frac{-2s_2t}{\tau}}\right)\left(\frac{e^{\frac{-2s_1t}{\tau}} a_2\left(0\right)}{a_1\left(0\right)a_2\left(0\right)-b\left(0\right)^2} + \frac{1}{s_1} - \frac{1}{s_1}e^{\frac{-2s_1t}{\tau}}\right)- \left(-\frac{e^{\frac{-s_2t}{\tau}} b\left(0\right) e^{\frac{-s_1t}{\tau}}}{a_1\left(0\right)a_2\left(0\right)-b\left(0\right)^2}\right)^2}, \end{align*}

and interchanging subscripts 1 and 2 yields $a_2(t)$. As a check on this result, by setting $b(0) = 0 $ we recover the expression

\begin{equation*} a_1\left(t\right) = \frac{a_1\left(0\right) }{e^{\frac{-2s_1t}{\tau}} + \frac{a_1\left(0\right)}{s_1}\left(1 -e^{\frac{-2s_1t}{\tau}}\right)}, \end{equation*}

from Saxe *et al* (2019).

We further simplify the denominator to

\begin{align*} &amp;\mathbf{A}^{\textrm T}\mathbf{A}\left(t\right) \nonumber \\ &amp;\quad =\frac{1}{\frac{1}{a_1\left(0\right)a_2\left(0\right)-b\left(0\right)^2}\left( e^{\frac{-2\left(s_1+s_2\right)t}{\tau}}\left(1-\frac{a_1\left(0\right)}{s_1}- \frac{a_2\left(0\right)}{s_2}\right)+ e^{\frac{-2s_2t}{\tau}}\frac{a_1\left(0\right)}{s_1} + e^{\frac{-2s_1t}{\tau}}\frac{a_2\left(0\right)}{s_2}\right) +\frac{1}{s_2s_1} } \nonumber\\ &amp;\qquad \times \begin{bmatrix} \frac{e^{\frac{-2s_2t}{\tau}} a_1\left(0\right)}{a_1\left(0\right)a_2\left(0\right)-b\left(0\right)^2} + \frac{1}{s_2} -\frac{1}{s_2}e^{\frac{-2s_2t}{\tau}} &amp; \frac{e^{\frac{-s_1t}{\tau}} b\left(0\right) e^{\frac{-s_2t}{\tau}} }{a_1\left(0\right)a_2\left(0\right)-b\left(0\right)^2} \\[3pt] \frac{e^{\frac{-s_2t}{\tau}} b\left(0\right) e^{\frac{-s_1t}{\tau}}}{a_1\left(0\right)a_2\left(0\right)-b\left(0\right)^2} &amp; \frac{e^{\frac{-2s_1t}{\tau}} a_2\left(0\right)}{a_1(0)a_2(0)-b(0)^2} + \frac{1}{s_1} - \frac{1}{s_1}e^{\frac{-2s_1t}{\tau}} \end{bmatrix}\nonumber \end{align*}

### E.3. Off-Diagonal decoupling dynamics

We track the decoupling by considering the dynamics of the off-diagonal element *b*(*t*).

\begin{equation*} b\left(t\right) = \frac{\frac{e^{\frac{-s_2t}{\tau}} b\left(0\right) e^{\frac{-s_1t}{\tau}}}{a_1\left(0\right)a_2\left(0\right)-b\left(0\right)^2} }{\frac{1}{a_1\left(0\right)a_2\left(0\right)-b\left(0\right)^2}\left( e^{\frac{-2\left(s_1+s_2\right)t}{\tau}}\left(1-\frac{a_1\left(0\right)}{s_1}- \frac{a_2\left(0\right)}{s_2}\right)+ e^{\frac{-2s_2t}{\tau}}\frac{a_1\left(0\right)}{s_1} + e^{\frac{-2s_1t}{\tau}}\frac{a_2\left(0\right)}{s_2}\right) +\frac{1}{s_2s_1} }. \end{equation*}

As *t* tends to infinity $ \lim_{t \to \infty} b(t) = 0$ the off-diagonal element shrinks to zero.

We can further simplify the off-diagonal to

\begin{equation*} b\left(t\right) = \frac{b\left(0\right)}{e^{\frac{-\left(s_1+s_2\right)t}{\tau}}\left(1-\frac{a_1\left(0\right)}{s_1}- \frac{a_2\left(0\right)}{s_2}\right)+ e^{\frac{\left(s_1-s_2\right)t}{\tau}}\frac{a_1\left(0\right)}{s_1} + e^{\frac{\left(s_2-s_1\right)t}{\tau}}\frac{a_2\left(0\right)}{s_2}+\frac{a_1\left(0\right)a_2\left(0\right)-b\left(0\right)^2}{s_2s_1}}. \end{equation*}

Equation (198) can exhibit non-monotonic trajectories with transient peaks as shown in figure 4. The qualitative observations for the $2\times2$ network hold for larger target matrices as shown in figure 9. For large initialisation, the dynamics are exponential. At intermediate and small initialisation, the maximum of the off-diagonal is reached before the singular mode is fully learned. In the small initialisation scheme, the peak is of negligible size. The respective target matrix for panel (A)–(D), (B)–(E) and (C)–(F) in figure 9 are

\begin{align*} \text{dense } \begin{bmatrix} 5&amp;6&amp;3&amp;0&amp;1 \\ 4,&amp;1&amp;0&amp;1&amp;2\\ 3&amp;0&amp;2&amp;4&amp;0 \\ 3&amp;4&amp;0&amp;3&amp;2 \\ 2&amp;0&amp;1&amp;3&amp;4\\ \end{bmatrix} \text{, diagonal } \begin{bmatrix} 5 &amp; 0 &amp; 0 &amp;0 &amp; 0 \\ 0 &amp; 1 &amp; 0 &amp;0 &amp; 0 \\ 0 &amp; 0 &amp; 2 &amp;0 &amp; 0 \\ 0 &amp; 0 &amp; 0 &amp; 3 &amp; 0 \\ 0 &amp; 0 &amp; 0 &amp;0 &amp; 4 \\ \end{bmatrix} \text{and equal diagonal } \begin{bmatrix} 5 &amp; 0 &amp; 0 &amp;0 &amp; 0 \\ 0 &amp; 5 &amp; 0 &amp;0 &amp; 0 \\ 0 &amp; 0 &amp; 5 &amp;0 &amp; 0 \\ 0 &amp; 0 &amp; 0 &amp; 5 &amp; 0 \\ 0 &amp; 0 &amp; 0 &amp;0 &amp; 5 \\ \end{bmatrix}. \end{align*}

We characterise these dynamics considering the case where $s_1 = s_2 = s$ for the two-by-two solution (i.e. equal diagonal target **y**) for which we can compute the time of the peak. In this particular case, we can further simplify the off-diagonal to

\begin{equation*} b\left(t\right) = \frac{b\left(0\right)}{e^{\frac{-2\left(s\right)t}{\tau}}\left(1-\frac{a_1\left(0\right)+a_2\left(0\right)}{s} \right)+\frac{a_1\left(0\right)+a_2\left(0\right)}{s} +\frac{a_1\left(0\right)a_2\left(0\right)-b\left(0\right)^2}{s^2}}. \end{equation*}

We find the time of the maximum of the off-diagonal elements to be $t_\textrm {peak} = \frac{\tau}{4s}\ln{\frac{s(s-a_1(0)- a_2(0))}{a_1(0)a_2(0)-b(0)^2}}$.

The presence of a peak in the off-diagonal values, indicates the decoupling, but as shown in figures 4(D)–(F), the peak size is negligible in comparison to the size of the on-diagonal values for small initial weights. This difference is reminiscent of the silent alignment effect described by Atanasov *et al* (2022). We further note, that the time scale of decoupling is on the same order as the one reported for the silent alignment effect $t_\textrm {sa} = \frac{1}{s}$.

### E.4. On-diagonal dynamics and the effect of initialisation variance

In this section we revisit the impact of initialisation scale for the on-diagonal dynamics. We now start with

\begin{align*} &amp;a_1\left(t\right) = \frac{ \frac{e^{\frac{-2s_2t}{\tau}} a_1\left(0\right)}{a_1\left(0\right)a_2\left(0\right)-b\left(0\right)^2} + \frac{1}{s_2} -\frac{1}{s_2}e^{\frac{-2s_2t}{\tau}}}{\frac{1}{a_1\left(0\right)a_2\left(0\right)-b\left(0\right)^2}\left( e^{\frac{-2\left(s_1+s_2\right)t}{\tau}}\left(1-\frac{a_1\left(0\right)}{s_1}- \frac{a_2\left(0\right)}{s_2}\right)+ e^{\frac{-2s_2t}{\tau}}\frac{a_1\left(0\right)}{s_1} + e^{\frac{-2s_1t}{\tau}}\frac{a_2\left(0\right)}{s_2}\right) +\frac{1}{s_2s_1} }. \end{align*}

The diagonal elements simplify in the cases where $s_1 = s_2 = s$ (i.e. target **Y** is diagonal),

\begin{align*} &amp;a_1\left(t\right) = \frac{ \frac{e^{\frac{-2st}{\tau}} a_1\left(0\right)}{a_1\left(0\right)a_2\left(0\right)-b\left(0\right)^2} + \frac{1}{s} -\frac{1}{s}e^{\frac{-2st}{\tau}}}{\frac{1}{a_1\left(0\right)a_2\left(0\right)-b\left(0\right)^2}\left( e^{\frac{-4st}{\tau}}\left(1-\frac{a_1\left(0\right)}{s}- \frac{a_2\left(0\right)}{s}\right)+ e^{\frac{-2st}{\tau}}\frac{a_1\left(0\right)}{s} + e^{\frac{-2st}{\tau}}\frac{a_2\left(0\right)}{s}\right) +\frac{1}{s^2} }. \end{align*}

We consider when $|a_1(0)|,|a_2(0)|,|b(0)|\ll 1$, and recover a sigmoidal trajectory,

\begin{equation*} a_1\left(t\right) = \frac{sa_1\left(0\right)}{e^{\frac{-2st}{\tau}}\left[s-a_1\left(0\right)-a_2\left(0\right)\right]+ a_1\left(0\right) +a_2\left(0\right)}. \end{equation*}

We can compute the time at which $a_1(t)$ rises to half its asymptotic value to be

\begin{equation*} t_{\textrm{half}} = \frac{\tau}{2s}\log\left(\frac{s-a_1\left(0\right) - a_2\left(0\right) }{a_1\left(0\right) - a_2\left(0\right)}\right). \end{equation*}

For $|a_1(0)|,|a_2(0)|,|b(0)| \gg 0$ the dynamics of the on-diagonal element *a*_1_ is close to exponential.

The observation for $2\times2$ network hold for larger target matrices as shown in figure 9. For large variance initialisations, the dynamics are exponential. At intermediate variance initialisations, we observe more complex behaviour. While at small variance initialisations, the on-diagonal element describes a sigmoidal trajectory.

## Appendix F Continual learning

We consider the case of training a two-layer deep linear network on a sequence of tasks $\mathcal{T}_a$, $\mathcal{T}_b$, $\mathcal{T}_c$, ... with corresponding correlation functions $\mathcal{T}_a = \mathbf{\tilde{\Sigma}}^{yx}_a$, $\mathcal{T}_b = \mathbf{\tilde{\Sigma}}^{yx}_b ...$. Then, the full batch loss of the *i*th task at any point in training time is

\begin{equation*} \mathcal{L}_i = \frac{1}{2P}\left|\left|\mathbf{W}_2\mathbf{W}_1\mathbf{X}_i - \mathbf{Y}_i\right|\right|_F^2. \end{equation*}

From theorem 3.2 it follows that after training the network to convergence on task $\mathcal{T}_j$, the network function is $\mathbf{W}_2\mathbf{W}_1 = {\tilde{\mathbf{U}}}{\tilde{\mathbf{S}}}\mathbf{\tilde{V}}^{\textrm T} = \mathbf{\tilde{\Sigma}}^{yx}_j$. Further, using the assumption of whitened inputs 2.2 and the identities $||A||_F^2 = \text{Tr}(AA^{\textrm T})$ and $\text{Tr}(A) + \text{Tr}(B) = \text{Tr}(A + B)$, the full batch loss of the *i*-th task is then

\begin{align*} \mathcal{L}_i\left(\mathcal{T}_j\right) &amp; = \frac{1}{2P}\left|\left|\boldsymbol{\tilde{\Sigma}}^{yx}_j\mathbf{X}_i - \mathbf{Y}_i\right|\right|_F^2 \end{align*}

\begin{align*} &amp; = \frac{1}{2P}\,\mathrm{Tr}\left(\left(\boldsymbol{\tilde{\Sigma}}^{yx}_j\mathbf{X}_i - \mathbf{Y}_i\right)\left(\boldsymbol{\tilde{\Sigma}}^{yx}_j\mathbf{X}_i - \mathbf{Y}_i\right)^{\textrm T}\right) \end{align*}

\begin{align*} &amp; = \frac{1}{2P}\,\mathrm{Tr}\left(\boldsymbol{\tilde{\Sigma}}^{yx}_j\mathbf{X}_i\mathbf{X}_i^{\textrm T}\boldsymbol{\tilde{\Sigma}}^{{yx}^{\textrm T}}_j\right) - \frac{1}{P}\,\mathrm{Tr}\left(\boldsymbol{\tilde{\Sigma}}^{yx}_j\mathbf{X}_i\mathbf{Y}_i^{\textrm T}\right) + \frac{1}{2P}\,\mathrm{Tr}\left(\mathbf{Y}_i\mathbf{Y}_i^{\textrm T}\right) \end{align*}

\begin{align*} &amp; = \frac{1}{2}\,\mathrm{Tr}\left(\boldsymbol{\tilde{\Sigma}}^{yx}_j\boldsymbol{\tilde{\Sigma}}^{{yx}^{\textrm T}}_j\right) - \,\mathrm{Tr}\left(\boldsymbol{\tilde{\Sigma}}^{yx}_j\boldsymbol{\tilde{\Sigma}}^{{yx}^{\textrm T}}_i\right) + \frac{1}{2}\,\mathrm{Tr}\left(\boldsymbol{\tilde{\Sigma}}^{yy}_i\right) \end{align*}

\begin{align*} &amp; = \frac{1}{2}\,\mathrm{Tr}\left(\left(\boldsymbol{\tilde{\Sigma}}^{yx}_j - \boldsymbol{\tilde{\Sigma}}^{yx}_i\right)\left(\boldsymbol{\tilde{\Sigma}}^{yx}_j - \boldsymbol{\tilde{\Sigma}}^{yx}_i\right)^{\textrm T} - \boldsymbol{\tilde{\Sigma}}^{yx}_i\boldsymbol{\tilde{\Sigma}}^{{yx}^{\textrm T}}_i\right) + \frac{1}{2}\left(\boldsymbol{\tilde{\Sigma}}^{yy}_i\right) \end{align*}

\begin{align*} &amp; = \frac{1}{2}\left|\left|\boldsymbol{\tilde{\Sigma}}^{yx}_j - \boldsymbol{\tilde{\Sigma}}^{yx}_i\right|\right|_F^2 \underbrace{- \frac{1}{2}\,\mathrm{Tr}\left( \boldsymbol{\tilde{\Sigma}}^{yx}_i\boldsymbol{\tilde{\Sigma}}^{{yx}^{\textrm T}}_i\right) + \frac{1}{2}\left(\boldsymbol{\tilde{\Sigma}}^{yy}_i\right)}_c. \end{align*}

Therefore, the amount of forgetting $\mathcal{F}$ on task $\mathcal{T}_i$ when training on task $\mathcal{T}_k$ after having trained the network on task $\mathcal{T}_j$, i.e. the relative change of loss, is fully determined by the similarity structure of the tasks

\begin{align*} \mathcal{F}_i\left(\mathcal{T}_j, \mathcal{T}_k\right) &amp; = \mathcal{L}_i\left(\mathcal{T}_k\right) - \mathcal{L}_i\left(\mathcal{T}_j\right) \end{align*}

\begin{align*} &amp; = \frac{1}{2}\left|\left|\boldsymbol{\tilde{\Sigma}}^{yx}_k - \boldsymbol{\tilde{\Sigma}}^{yx}_i\right|\right|_F^2 + c - \frac{1}{2}\left|\left|\boldsymbol{\tilde{\Sigma}}^{yx}_j - \boldsymbol{\tilde{\Sigma}}^{yx}_i\right|\right|_F^2 - c \end{align*}

\begin{align*} &amp; = \frac{1}{2}\left(\left|\left|\boldsymbol{\tilde{\Sigma}}^{yx}_k - \boldsymbol{\tilde{\Sigma}}^{yx}_i\right|\right|_F^2 - \left|\left|\boldsymbol{\tilde{\Sigma}}^{yx}_j - \boldsymbol{\tilde{\Sigma}}^{yx}_i\right|\right|_F^2\right). \end{align*}

## Appendix G Revising structured knowledge

### G.1. Reversal learning dynamics

In the following, we assume that the input dimension is equal to the output dimension. Further, we denote the *i*-th column of the left and right singular vectors as **u**_*i*_, $\tilde{\mathbf{u}}_i$ and **v**_*i*_, $\tilde{\mathbf{v}}_i$ respectively.

Reversal learning occurs when the task and the initial network function share the same left and right singular vectors, i.e. $\mathbf{U} = {\tilde{\mathbf{U}}}$ and $\mathbf{V} = \mathbf{\tilde{V}}$, except for one or multiple columns of the left singular vectors, for which the direction is reversed:

\begin{equation*} -\mathbf{u}_i = \tilde{\mathbf{u}}_i\text{.} \end{equation*}

We note that, if there is any reversal in the right singular vectors $-\mathbf{v}_i = \tilde{\mathbf{v}}_i$, this can be written as a reversal in the left singular vectors, as the signs of the right and left singular vectors are interchangeable. In the reversal learning setting, both $\mathbf{B} = \mathbf{U}^{T}{\tilde{\mathbf{U}}} +\mathbf{V}^{T}\mathbf{\tilde{V}}$ and $\mathbf{C} = \mathbf{U}^{T}{\tilde{\mathbf{U}}} -\mathbf{V}^{T}\mathbf{\tilde{V}}$ are diagonal matrices. The diagonal entries of **C** are zero if the singular vectors are aligned and 2 if they are reversed. Similarly, diagonal entries of **B** are 2 if the singular vectors are aligned and zero if they are reversed. Therefore, in the case of reversal learning, **B** is a diagonal matrix with 0 values and thus is not invertible. As a consequence, the learning dynamics cannot be described by equation (11). However, as **B** and **C** are diagonal matrices, the learning dynamics simplify. Let **b**_*i*_, **c**_*i*_, **s**_*i*_ and $\tilde{\mathbf{s}}_i$ denote the *i*-th diagonal entry of **B**, **C**, **S** and ${\tilde{\mathbf{S}}}$ respectively, then the network dynamics can be rewritten as

\begin{align*} \mathbf{W}_2\mathbf{W}_1\left(t\right) &amp;= \frac{1}{2}{\tilde{\mathbf{U}}}\left(e^{{\tilde{\mathbf{S}}}\frac{t}{\tau}}\mathbf{B}^{\textrm T} + e^{-{\tilde{\mathbf{S}}}\frac{t}{\tau}}\mathbf{C}^{\textrm T}\right) \nonumber \\ &amp; \quad \times \left[\mathbf{S}^{-1} + \frac{1}{4} \mathbf{B}\left(e^{2{\tilde{\mathbf{S}}}\frac{t}{\tau}} - \mathbf{I}\right){\tilde{\mathbf{S}}}^{-1}\mathbf{B}^{\textrm T} - \frac{1}{4} \mathbf{C}\left(e^{-2{\tilde{\mathbf{S}}}\frac{t}{\tau}} - \mathbf{I}\right){\tilde{\mathbf{S}}}^{-1}\mathbf{C}^{\textrm T} \right]^{-1} \end{align*}

\begin{align*} &amp; \frac{1}{2}\left(e^{{\tilde{\mathbf{S}}}\frac{t}{\tau}}\mathbf{B} - e^{-{\tilde{\mathbf{S}}}\frac{t}{\tau}}\mathbf{C}\right){\tilde{\mathbf{V}}}^{\textrm T} \nonumber\\ &amp; \quad = \sum_{i = 1}^{N_\textrm i} \frac{\mathbf{b}_i^2e^{2\tilde{\mathbf{s}}_i\frac{t}{\tau}} - \mathbf{c}_i^2e^{-2\tilde{\mathbf{s}}_i\frac{t}{\tau}}}{4\mathbf{s}_i^{-1} + \mathbf{b}_i^2e^{2\tilde{\mathbf{s}}_i\frac{t}{\tau}}\tilde{\mathbf{s}}_i^{-1} - \mathbf{b}_i^2\tilde{\mathbf{s}}_i^{-1} - \mathbf{c}_i^2e^{-2\tilde{\mathbf{s}}_i\frac{t}{\tau}}\tilde{\mathbf{s}}_i^{-1}+\mathbf{c}_i^2\tilde{\mathbf{s}}_i^{-1}}\ \tilde{\mathbf{u}}_i\tilde{\mathbf{v}}_i^{\textrm T} \end{align*}

\begin{align*} &amp;\quad = \sum_{i = 1}^{N_\textrm i} \frac{\mathbf{s}_i\mathbf{b}_i^2\tilde{\mathbf{s}}_i - \mathbf{s}_i\mathbf{c}_i^2\tilde{\mathbf{s}}_ie^{-4\tilde{\mathbf{s}}_i\frac{t}{\tau}}}{4\tilde{\mathbf{s}}_ie^{-2\tilde{\mathbf{s}}_i\frac{t}{\tau}} + \mathbf{s}_i\mathbf{b}_i^2\left(1 - e^{-2\tilde{\mathbf{s}}_i\frac{t}{\tau}}\right) + \mathbf{s}_i\mathbf{c}_i^2\left(e^{-2\tilde{\mathbf{s}}_i\frac{t}{\tau}} - e^{-4\tilde{\mathbf{s}}_i\frac{t}{\tau}}\right)} \tilde{\mathbf{u}}_i\tilde{\mathbf{v}}_i^{\textrm T}\text{.} \end{align*}

It follows, that in the reversal learning case, i.e. $\mathbf{b} = 0$, for each reversed singular vector, the dynamics vanish to zero

\begin{equation*} \lim_{t\to\infty}\frac{- \mathbf{s}_i\mathbf{c}_i^2\tilde{\mathbf{s}}_ie^{-4\tilde{\mathbf{s}}_i\frac{t}{\tau}}}{4\tilde{\mathbf{s}}_ie^{-2\tilde{\mathbf{s}}_i\frac{t}{\tau}} + \mathbf{s}_i\mathbf{c}_i^2\left(e^{-2\tilde{\mathbf{s}}_i\frac{t}{\tau}} - e^{-4\tilde{\mathbf{s}}_i\frac{t}{\tau}}\right)} \tilde{\mathbf{u}}_i\tilde{\mathbf{v}}_i^{\textrm T} = 0\text{.} \end{equation*}

Analytically, the learning dynamics are initialised and remain on the separatrix of a saddle point, until the corresponding singular value of the network function has vanished and remains zero, corresponding to convergence to the saddle point. When simulated numerically, the learning dynamics escape the saddle points due to imprecision of floating point arithmetic. However, numerical optimisation still suffers from catastrophic slowing (Lee *et al* 2022), as escaping the saddle point takes time (figure 6(A)). In contrast, in the case of aligned singular vectors ($\mathbf{c} = 0$), we recover the equation for the temporal dynamics as described in Saxe *et al* (2014). Training succeeds, as the singular value of the network function converges to its target value

\begin{align*} \lim_{t\to\infty}\sum_{i = 1}^{N_\textrm i} \frac{\mathbf{s}_i\mathbf{b}_i^2\tilde{\mathbf{s}}_i}{4\tilde{\mathbf{s}}_ie^{-2\tilde{\mathbf{s}}_i\frac{t}{\tau}} + \mathbf{s}_i\mathbf{b}_i^2\left(1 - e^{-2\tilde{\mathbf{s}}_i\frac{t}{\tau}}\right)} \tilde{\mathbf{u}}_i\tilde{\mathbf{v}}_i^{\textrm T} &amp; = \frac{\mathbf{s}_i\mathbf{b}_i^2\tilde{\mathbf{s}}_i}{\mathbf{s}_i\mathbf{b}_i^2}\tilde{\mathbf{u}}_i\tilde{\mathbf{v}}_i^{\textrm T} \end{align*}

\begin{align*} &amp; = \tilde{\mathbf{s}}_i\tilde{\mathbf{u}}_i\tilde{\mathbf{v}}_i^{\textrm T}\text{.} \end{align*}

In summary, in the case of aligned singular vectors, the learning dynamics can be described by the convergence of singular values. However in the case of reversal learning, analytically, training does not succeed. In simulations, the learning dynamics escape the saddle point due to numerical imprecision, but the learning dynamics are catastrophically slowed in the vicinity of the saddle point.

### G.2. Exact learning dynamics in shallow networks

To provide a point of comparison to our deep linear network results, here we derive a solution for the temporal dynamics of reversal learning in a shallow network.

The network’s weights are optimised using full batch gradient descent with learning rate *η* (or equivalently time constant $\tau = 1/\eta$) on the mean squared error loss given in equation (2), yielding the first task dynamics

\begin{align*} \tau{\frac{\textrm{d}}{d t}}{\mathbf{W}} = \boldsymbol{\tilde{\Sigma}}^{yx}-{\mathbf{W}}\boldsymbol{\tilde{\Sigma}}^{xx}, \end{align*}

where $\mathbf{\tilde{\Sigma}}^{xx}$ and $\mathbf{\tilde{\Sigma}}^{yx}$ is the input and input-output correlation matrices of the dataset. We define

\begin{align*} \mathrm{SVD}\left(\mathbf{W}\left(0\right)\right) = \mathbf{U}\mathbf{S}\mathbf{V}^{\textrm T} \text{and}\,\, \mathrm{SVD}\left(\boldsymbol{\tilde{\Sigma}}^{yx}\right) = {\tilde{\mathbf{U}}}{\tilde{\mathbf{S}}}{\tilde{\mathbf{V}}}^{\textrm T} \text{.} \end{align*}

motivating the change of variable $\mathbf{W} = \mathbf{U}\boldsymbol{\overline{W}}\mathbf{V}^{\textrm T}$. We project the weight into the basis of the initialisation

\begin{align*} \tau {\frac{\textrm{d}}{d t}}\mathbf{U}{\mathbf{\overline{W}}}\mathbf{V}^{\textrm T} &amp; = \boldsymbol{\tilde{\Sigma}}^{yx}-{\mathbf{U}{\mathbf{\overline{W}}}\mathbf{V}^{\textrm T}}\boldsymbol{\tilde{\Sigma}}^{xx} \end{align*}

\begin{align*} \tau {\frac{\textrm{d}}{d t}}\mathbf{U}{\mathbf{\overline{W}}}\mathbf{V}^{\textrm T} &amp; = \mathbf{U}\mathbf{U}^{\textrm T}\boldsymbol{\tilde{\Sigma}}^{yx}\mathbf{V}\mathbf{V}^{\textrm T}-{\mathbf{U}{\mathbf{\overline{W}}}\mathbf{V}^{\textrm T}}\boldsymbol{\tilde{\Sigma}}^{xx} \end{align*}

\begin{align*} \tau {\frac{\textrm{d}}{d t}}{\mathbf{\overline{W}}} &amp; = \mathbf{U}^{\textrm T}\boldsymbol{\tilde{\Sigma}}^{yx}\mathbf{V}-{{\mathbf{\overline{W}}}}\boldsymbol{\tilde{\Sigma}}^{xx}. \end{align*}

Under the assumption of whitened inputs 2.2, the dynamics yields

\begin{align*} \tau {\frac{\textrm{d}}{d t}}{\mathbf{\overline{W}}} &amp; = \mathbf{U}^{\textrm T}\boldsymbol{\tilde{\Sigma}}^{yx}\mathbf{V}-{{\mathbf{\overline{W}}}}. \end{align*}

Defining $\boldsymbol{\overline{W}}_{ii} = b_{i}$ the diagonal element of the matrix, encoding the strength of the mode *i* transmitted by the input-to-output weight. Similarly, we write ${(\mathbf{U}^{\textrm T}\mathbf{\tilde{\Sigma}}^{yx}\mathbf{V})}_{ii} = k_{i}$. Assuming decoupled initial conditions, we obtain the scalar dynamics

\begin{align*} \tau {\frac{\textrm{d}}{\textrm dt}}b_{i} = k_{i}-{b_{i}} \end{align*}

with solution

\begin{equation*} b_{i} = k_{i}\left(1-e^{\frac{-t}{\tau}}\right) + b_{i}^{0} e^{\frac{-t}{\tau}}. \end{equation*}

Reverting the change of variable, the weight trajectory yields

\begin{equation*} \mathbf{W} = \mathbf{U} \mathbf{B}\left(t\right)\mathbf{V}^{\textrm T}. \end{equation*}

This solution is very similar to the one proposed by Saxe *et al* (2019). However, the key here is that *k*_*i*_ can have negative values. *k*_*i*_ is negative whenever a vector is in the opposite direction to the initialisation (as in the reversal learning setting). We show in figure 6 that the analytical solution derived above matches the numerical temporal dynamics. From equation (228), we note that the shallow network cannot display catastrophic slowing.

## Appendix H Simulations

In the following, we describe the details of the simulation studies. Generally, $N_\textrm i$, $N_\textrm h$ and $N_\textrm o$ denote the dimension of the input, hidden layer and output (target) respectively. The number of training samples is *N* and the learning rate is denoted by $\eta = \frac{1}{\tau}$.

### H.1. Zero-balanced weight initialisation

The initial network weights are zero-balanced 2.3 when they satisfy

\begin{equation*} \mathbf{W}_1\left(0\right)\mathbf{W}_1\left(0\right)^{\textrm T} = \mathbf{W}_2\left(0\right)^{\textrm T}\mathbf{W}_2\left(0\right)\text{.} \end{equation*}

In practice, we use algorithm 1 to initialise the network weights, where *α* is a scaling factor which is used to control the variance of the weights, i.e. to vary between small and large weight initialisations.

**Table:**

**Algorithm 1.** Zero-balanced weight initialisation.
**Require**: $N_\textrm i, N_\textrm h, N_\textrm o, \sigma$
**W** $_{1} \sim \mathcal{N}(\mu = 0, \sigma) \in \mathbb{R}^{N_\textrm h\times N_\textrm i}$
**W** $_{2} \sim \mathcal{N}(\mu = 0, \sigma) \in \mathbb{R}^{N_\textrm o\times N_\textrm h}$
$\mathbf{U}, \mathbf{S}, \mathbf{V} \gets \mathrm{SVD}(\mathbf{W}_2\mathbf{W}_1)$
$\mathbf{S} \gets \sqrt{\mathbf{S}}$
$\mathbf{R} \sim \mathcal{N}(\mu = 0, \sigma = 1) \in \mathbb{R}^{N_\textrm h\times N_\textrm h}$
$\mathbf{R}, \_, \_ \gets \mathrm{SVD}(\mathbf{R})$
**if** $N_\textrm i \neq N_\textrm o$ **then**
$N_s \gets N_\textrm i\ \mathbf{if}\, N_\textrm i < N_\textrm o\, \mathbf{else}\, N_\textrm o$
$\mathbf{S}_1 \gets \begin{bmatrix} \mathbf{S}\\ \mathbf{0}_{N_\textrm h - N_s \times N_s} \end{bmatrix}$
$\mathbf{S}_2 \gets \begin{bmatrix} \mathbf{S} &amp; \mathbf{0}_{N_s \times N_\textrm h - N_s} \end{bmatrix}$
$\mathbf{W}_1 \gets \mathbf{R} \mathbf{S}_1 \mathbf{V}^{\textrm T}$
$\mathbf{W}_2 \gets \mathbf{U} \mathbf{S}_2 \mathbf{R}^{\textrm T}$
**else**
$\mathbf{W}_1 \gets \mathbf{R} \mathbf{S} \mathbf{V}^{\textrm T}$
$\mathbf{W}_2 \gets \mathbf{U} \mathbf{S} \mathbf{R}^{\textrm T}$
**end if**
**return** $\mathbf{W}_1 \mathbf{W}_2$

### H.2. Tasks

In the following, we describe the different tasks that are used throughout the simulation studies.

#### H.2.1. Random regression task.

In a random regression task the inputs $\mathbf{X} \in \mathbb{R}^{N_\textrm i, N}$ are sampled from a random normal distribution $\mathbf{X} \sim \mathcal{N}(\mu = 0, \sigma = 1)$. The input data **X** is then whitened, such that $\frac{1}{N}\mathbf{X}\mathbf{X}^{\textrm T} = \mathbf{I}$. The target values $\mathbf{Y} \in \mathbb{R}^{N_\textrm o, N}$ are also sampled from a random normal distribution, however, with variance adjusted to the number of output nodes $\mathbf{Y} \sim \mathcal{N}(\mu = 0, \alpha = \frac{1}{\sqrt{N_\textrm o}})$. Thus, network inputs and target values are uncorrelated Gaussian noise and therefore, a linear solution does not always exist.

#### H.2.2. Teacher-student task.

In order to guarantee that a linear solution exists, we use the teacher-student setup. First, inputs **X** are sampled as in the random regression task. Then, target values **Y** are generated by sampling a pair of random zero-balanced weights $\mathbf{W}_1 \in \mathbb{R}^{N_\textrm h \times N_\textrm i}$ and $\mathbf{W}_2 \in \mathbb{R}^{N_\textrm o \times N_\textrm h}$ and then calculating $\mathbf{Y} = \mathbf{W}_2\mathbf{W}_1\mathbf{X}$. Like this, it is ensured that a linear solution exists. The variance of the output is varied by changing the variation within the zero-balanced weights *σ*.

#### H.2.3. Semantic hierarchy.

Input items in the semantic hierarchy task are encoded as one-hot vectors, i.e. $\mathbf{X} = \mathbf{I}$. The corresponding target vectors *y_i_* encoded the position in the hierarchical tree. Where a 1 encoded being a left child of a node, a −1 encoded being a right child of a node and a 0 encoded that the item is not a child of that node. For example, the blue fish is a blue fish, it is a left child of the root node, a left child of the animal node, not part of the plant branch, a right child of the fish node, and not part of the bird, algae or flower branch, leading to the label $[1, 1, 1, 0, -1, 0, 0, 0]$. The labels for all objects in the semantic tree as depicted in figure 3(A) is then

\begin{align*} \mathbf{Y} = \begin{bmatrix} 1 &amp; 1 &amp; 1 &amp; 1 &amp; 1 &amp; 1 &amp; 1 &amp; 1 \\ 1 &amp; 1 &amp; 1 &amp; 1 &amp; -1 &amp; -1 &amp; -1 &amp; -1 \\ 1 &amp; 1 &amp; -1 &amp; -1 &amp; 0 &amp; 0 &amp; 0 &amp; 0\\ 0 &amp; 0 &amp; 0 &amp; 0 &amp; 1 &amp; 1 &amp; -1 &amp; -1 \\ 1 &amp; -1 &amp; 0 &amp; 0 &amp; 0 &amp; 0 &amp; 0 &amp; 0 \\ 0 &amp; 0 &amp; 1 &amp; -1 &amp; 0 &amp; 0 &amp; 0 &amp; 0 \\ 0 &amp; 0 &amp; 0 &amp; 0 &amp; 1 &amp; -1 &amp; 0 &amp; 0 \\ 0 &amp; 0 &amp; 0 &amp; 0 &amp; 0 &amp; 0 &amp; 1 &amp; -1 \\ \end{bmatrix}. \end{align*}

The singular value decomposition for the corresponding correlation matrix $\mathbf{\tilde{\Sigma}}^{yx}$ are not unique. The first two, the third and the fourth and the last four singular values are identical. In order to match the numerical and analytical solution, this permutation invariance is removed by adding a small constant perturbation to each column $\mathbf{y}_i, i \in {1, {\ldots}, N}$ of the labels

\begin{equation*} \mathbf{y}_i = \mathbf{y}_i * \left(1 + \frac{0.1}{i}\right), \end{equation*}

leading to almost but not exactly identical singular values.

#### H.2.4. Colour hierarchy.

Following the same procedure as described for the semantic hierarchy, the labels for the colour hierarchy as depicted in figure 6(C) are then

\begin{align*} \mathbf{Y} = \begin{bmatrix} 1 &amp; 1 &amp; 1 &amp; 1 &amp; 1 &amp; 1 &amp; 1 &amp; 1 \\ -1 &amp; 1 &amp; 1 &amp; -1 &amp; 1 &amp; 1 &amp; -1 &amp; -1 \\ 0 &amp; -1 &amp; 1 &amp; 0 &amp; -1 &amp; 1 &amp; 0 &amp; 0\\ 1 &amp; 0 &amp; 0 &amp; -1 &amp; 0 &amp; 0 &amp; -1 &amp; 1 \\ 0 &amp; 0 &amp; 1 &amp; 0 &amp; 0 &amp; -1 &amp; 0 &amp; 0 \\ 0 &amp; -1 &amp; 0 &amp; 0 &amp; 1 &amp; 0 &amp; 0 &amp; 0 \\ 1 &amp; 0 &amp; 0 &amp; 0 &amp; 0 &amp; 0 &amp; 0 &amp; -1 \\ 0 &amp; 0 &amp; 0 &amp; 1 &amp; 0 &amp; 0 &amp; -1 &amp; 0 \\ \end{bmatrix}. \end{align*}

### H.3. Figure 1

Figure 1 panels (B)–(D) show three simulations from varying initial weights on the same teacher-student task. The task was created with *σ* = 0.35. Farther, $N_\textrm i = 5$, $N_\textrm h = 10$, $N_\textrm o = 2$ and *N* = 10. The learning rate was *η* = 0.1 and the initial network weights were sampled with *σ* = 0.01, *σ* = 0.25 and *σ* = 0.25 in panels (B), (C) and (D) respectively.

### H.4. Figure 2

Figure 2 panels (A) and (B) show a simulation on the same teacher-student task (*σ* = 0.25), once from small initial weights (*σ* = 0.01) and once from large initial weights (*σ* = 0.15). Dimensions were $N_\textrm i = 4$, $N_\textrm h = 5$, $N_\textrm o = 3$ and *N* = 10 and the learning rate was *η* = 0.05. Panel (C) was generated by running 50 simulations, each with a different initial random seed. For each of the simulations, dimensions were sampled randomly, such that $N_\textrm i \in [2, 50]$, $N_\textrm o \in [2, 50]$, $N_\textrm h = [\min(N_\textrm i, N_\textrm o), 50]$ and $N \in [2\max(N_\textrm i, N_\textrm h, N_\textrm o), 3\max(N_\textrm i, N_\textrm h, N_\textrm o)]$. Then, a random regression task was generated. Subsequently, a linear network was initialised with $\sigma \sim \mathcal{U}[\frac{0.01}{\sqrt{\max(N_\textrm i, N_\textrm o, N_\textrm h)}}, \frac{0.5}{\sqrt{\max(N_\textrm i, N_\textrm o, N_\textrm h)}}]$. The network was then trained until convergence on the same task from the same initial weights for seven different learning rates $\eta \in \{0.05, 0.0232, 0.0107, 0.005, 0.0023, 0.0011, 0.0005\}$.

### H.5. Figure 3

Panels (C)–(F) in figure 3 were generated by training a linear network with $N_\textrm i = 8$, $N_\textrm h = 14$, $N_\textrm o = 8$ on the *N* = 8 items of the semantic hierarchy task. The learning rate was *η* = 0.05 and the initial weights in panels (C), (D) and (E) were sampled from a normal distribution with *σ* = 0.0001 and *σ* = 0.42 and zero-balanced weights with *σ* = 0.44 respectively.

### H.6. Figure 4

Figure 4 panel (A) was generated by training a linear network with $N_\textrm i = 5$, $N_\textrm h = 10$, $N_\textrm o = 5$ on the target **Y** as shown in equation (198) (equal diagonal). The network was initialised with *σ* = 0.1. The learning rate was *η* = 0.01.

Figure 4 panels (D)–(F) was generated by training a linear network with $N_\textrm i = 2$, $N_\textrm h = 10$, $N_\textrm o = 2$ on the target **Y** as shown in figure 4(C) and input $\mathbf{X} = bfi$. The network was initialised with small *σ* = 0.000 01, intermediate *σ* = 0.3 and large *σ* = 2 synaptic weights. The learning rate was *η* = 0.0001.

### H.7. Figure 5

Figure 5 panel (A) was generated by training a linear network with $N_\textrm i = 5$, $N_\textrm h = 10$, $N_\textrm o = 6$ subsequently on four different random regression tasks with *N* = 25. The learning rate was *η* = 0.05 and the initial weights were small (*σ* = 0.0001).

Panels (B) and (C) were generated by running 50 simulations on two subsequent random regression tasks, each with a different initial random seed. The simulation was repeated three times, the first time with a linear, the second time with a tanh and the last time with a ReLU activation function in the hidden layer. Dimension were randomly sampled such that $N_\textrm i \in [2, 30]$, $N_\textrm o \in [2, 30]$, $N_\textrm h = [\min(N_\textrm i, N_\textrm o), 30]$ and *N* = 100. The standard deviation of the initial weight was chosen such that $\sigma = \frac{0.5}{\sqrt{0.5(N_\textrm i + N_\textrm h)}}$. The learning rate was *η* = 0.075.

For panel (D) and (E) the same simulation was repeated for three times, the first time with a linear, the second time with a tanh and the last time with a ReLU activation function. Each time, five random regression tasks with dimensions $N_\textrm i = 15$, $N_\textrm h = 18$, $N_\textrm o = 21$ and *N* = 50 were generated. Then a network with initial weight scale *α* = 0.025 was sequentially trained with learning rate *η* = 0.1 on the five random regression tasks.

### H.8. Figure 6

Figure 6 panel (A) was generated by training a linear network with $N_\textrm i = 4$, $N_\textrm h = 6$, $N_\textrm o = 4$ on a reversal learning task (see section G.1), which was derived from a random regression task. The learning rate was *η* = 0.05 and initial weights had a standard deviation of *σ* = 0.25. Panel (B) was generated by training a shallow linear network (see section G.2) on the same reversal learning task, with identical hyperparameters as in panel (A).

For the top and bottom rows of panels (E) and (F) a linear network with $N_\textrm i = 8$, $N_\textrm h = 14$, $N_\textrm o = 8$ was trained on the semantic hierarchy task, followed by training the network on the adapted semantic hierarchy as depicted in figure 6(C) top, which is a reversal learning task and the colour hierarchy respectively. The learning rate was *η* = 0.05 and *σ* was set to 0.001 and 0.35 respectively.
